# HerbComb: An integrated database for the discovery of novel combinational therapies from herbal medicines

**DOI:** 10.1016/j.csbj.2025.10.065

**Published:** 2025-11-06

**Authors:** Yinyin Wang, Rui Liu, Xiang Luo, Jiaqi Yao, Hao Liu, Chengyuan Yue, Boon Seng Kho, Xiaochuang Xu, Shixing Lai, Fangcheng Yu, Yinnan Zhang, Lin Cao, Ninghua Tan, Yun Tang, Tao Yang, Jing Tang

**Affiliations:** a Department of TCMs Pharmaceuticals, School of Traditional Chinese Pharmacy, China Pharmaceutical University, Nanjing 211198, China; b Artificial Intelligence and Information Technology College, Nanjing University of Chinese Medicine, Nanjing 210023, China; c Shanghai Key Laboratory of New Drug Design, School of Pharmacy, East China University of Science and Technology, Shanghai 200237, China; d Wuhan Xinshidian Information Technology Co. Ltd, Wuhan 430023, China; e Jiangsu Province Engineering Research Center of TCM Intelligence Health Service, Nanjing University of Chinese Medicine, Nanjing 210023, China; f Research Program in Systems Oncology, Faculty of Medicine, University of Helsinki, FI-00014, Finland

**Keywords:** TCM database, Drug repositioning, Network proximity, Drug combination, Data integration

## Abstract

Drug combinations have gained increasing interest due to their potential for optimal efficacy and for overcoming drug resistance. Herbal medicine is a complex system with multiple ingredients and has illustrated apparent therapeutic effects through thousands of years of clinical experience, offering a rich resource for combinational therapies. However, the underlying mechanisms of their synergistic therapeutic effects in disease treatment remain obscure for most herbal medicines. To fill this gap, we developed an integrated database, HerbComb (https://herbcomb.com/), to discover combinatorial therapies in herbal medicine, featuring three unique functions: drug prepositioning, customized combinatorial analysis, and biological and pharmacological features. Additionally, transcriptional gene signatures and ADMET properties were also interrogated as interactive functionalities to facilitate combinational analysis. Using Tongxinluo for Stroke as a case study, we identified and validated Oleanolic acid and Ferulic acid as synergistic ingredients. In summary, HerbComb is a versatile data exploration platform designed to characterize synergistic interactions among herbal medicines, enhance understanding of synergistic mechanisms, and facilitate the discovery of effective drug combinations.

## 1 Introduction

In recent years, clinicians have increasingly adopted combination therapies to achieve optimal therapeutic effects [1]. Traditional Chinese Medicine (TCM) has demonstrated a unique paradigm of combination therapies, as many herbal formulae employ a multi-component, multi-target, and multi-pathway strategy to yield the intended therapeutic efficacy [2], [3]. Although herbal formulae have shown clinical efficacy, the underlying synergistic interactions among their components remain poorly understood [4], [5], [6], hindering their broader approval and applications.

To harness the therapeutic potential of herbal medicines, advanced computational methods are developed to elucidate their synergistic interactions at the molecular level [7]. In particular, network-based methods have gained increasing popularity for revealing deeper intrinsic connections within these complex relationships [8], [9]. Integration of herb–ingredient–target–disease helps uncover the underlying complex associations, especially for TCM. Many databases have been developed to support multifaceted network modeling, elucidating the system-level associations between formulas, herbs, ingredients, targets, and diseases. Earlier generations of TCM databases, such as TCM-ID [10], TCM Database@Taiwan [11], TCMSP [10], and TCMID [12], primarily offered herb-ingredient-target-disease associations with limited modelling capacities. Subsequent advancements led to the construction of more comprehensive network models for TCM, exemplified by TCM-Mesh and an update of TCMID. More recently, databases such as BATMAN-TCM [13], ETCM [14], YaTCM [15], ccTCM [16], and TCMAnalyzer [17] have enhanced mechanisms for action analysis. Furthermore, the SymMap database [18] and TCMM database [19] connected TCM symptoms to modern medical symptoms and diseases. Dai et al. [20] further employed a network pharmacology-based endophenotype network approach to identify disease-related active components. In addition, the MassKG database [21] integrates knowledge-driven mass spectrometry fragmentation models with deep-learning–based molecular generation methods, enabling automatic annotation and discovery of novel compound structures.

Despite the growing volume of data on TCM, current databases primarily focus on herb/ingredient-disease associations by aggregating target information for individual ingredients, which is subsequently linked to diseases for conventional pathway enrichment analysis [22]. However, the therapeutic associations between herbs and diseases are believed to arise from more intricate, systems-level interactions that go beyond the simple additive effects of individual components. Especially, the synergistic effect among ingredients/herbs is critical for their therapeutic effects, yet it is rarely considered in existing databases. To address this fundamental gap, we developed HerbComb, a platform whose core innovation is a network-proximity-based inference methodology. This approach has been rigorously validated through statistical analyses, including permutation tests and cross-disease benchmarking, demonstrating high accuracy in discriminating synergistic herb pairs [23]. The efficacy of this methodology is further supported by its successful application in elucidating the molecular basis of herbal treatments for various conditions [24], [25], [26], [27]. Building upon this validated foundation, we have extended the network paradigm to construct a comprehensive atlas of herbal combinations, integrating herb-herb, ingredient-ingredient, and herb-disease-ingredient interactions into a unified model. HerbComb thus serves as a sophisticated data portal that not only provides diverse pharmacological data but also actively supports combinatorial analysis and drug repurposing through its computational engine.

Distinct from existing TCM resources, HerbComb offers several unique, interactive functionalities: 1) Systematic drug repurposing: It enables large-scale modeling to identify potential therapeutic and synergistic ingredients/herbs for specific diseases, complemented by visual combinatorial networks to elucidate putative synergistic mechanism; 2) Customized combinatorial analysis: The platform allows users to perform tailored investigations of specific herb/ingredient combinations against diseases, effectively simulating the decision-making process in clinical herbal practice; 3) Integrated biological and pharmacological profiling: To contextualize combination predictions, HerbComb incorporates multiple layers of data, including experimentally derived gene expression signatures and predicted ADMET properties, as interactive features; 4) Efficient multi-dimensional search: Unlike conventional databases that require sequential queries, HerbComb enables simultaneous retrieval of all related herbs, ingredients, diseases, and prescriptions from a single search entry, dramatically improving analytical efficiency. Furthermore, the resource is underpinned by extensive manual curation of approximately 4000 publications, providing a foundational corpus of experimentally documented interactions. The integration of gene expression signatures from 693 herbal treatments addresses a critical data gap, allowing the identification of perturbation-driven molecular pathways and thereby facilitating a more profound, mechanism-based understanding of herbal pharmacology.

In summary, HerbComb represents a comprehensive, interactive platform that supports diverse research paradigms through its embedded network-based synergy inference and combination modeling engine.

## 2 Data collection and modelling

### 2.1 Data extraction and standardization

We extracted all available data from 15 TCM databases. Herb names were standardized to Pinyin, and ingredients from different databases were standardized using their InChIKey identifiers. Herbal formulae were collected from six databases, including TCM-ID [13], TCMID [28], YaTCM [15], ETCM [29], TCMIO [30], and TCMAnalyzer [17]. Compound-target profiles were extracted from public databases, including ChEMBL [31], BindingDB [32], GtopDB [33], DrugBank [34], DGiDB [35], and STITCH [36]. Additionally, we applied a wSDTNBI methodology to predict target proteins of herbal ingredients based on structural similarity between herbal ingredients and drugs [37]. Disease-target associations were extracted from a Multi-Scale Interactome (MSI) model [38], [39]. Diseases were standardized using the UMLS database [40], which maps disease terms from multiple data sources, such as ICD-10, MeSH, and SNOMED CT, to a unique CUI (Concept Unique Identifier). While all the targets were standardized via the UniProt API [41]. 119 ADMET properties were predicted by admetSAR 3.0 [42] for 49,285 herbal ingredients. We retrieved drug-combination records for herbal medicines from ∼4000 publications published between 2000 and 2023 that contained the keywords “TCM,” “herbal medicine,” “combination,” or “synergy.” Herbal perturbation data were retrieved from various resources, including the HERB [43] and ITCM [44] databases. Duplicated data of herb-ingredient, ingredient-target, and disease-target relationships were removed to ensure data quality for subsequent analysis.

### 2.2 Network-based inference of synergistic ingredients and herbs

Network proximity methods were employed to further explore the combinational relationships among prescriptions, calculating ingredient-ingredient, herb-herb, ingredient-disease, and herb-disease interactions. To explore synergistic ingredients in herbal medicines, the network proximity method [23], [45] was employed to determine an interaction score for an ingredient pair as follows:

#### 2.2.1 Ingredients-ingredient synergistic score

For all potential ingredient pairs within a single formula, we used a closest-distance algorithm based on their target networks to calculate their combined distance. The closest distance *d*^*C*^ is defined as follows:

(1) $$C\mathrm{losest}:\langle d_{\mathrm{AB}}^{C}\rangle =\frac{1}{\left|A\right|+|B|}\left(\sum_{a\in A}d\left(a,b\right)+\sum_{b\in B}d\left(a,b\right)\right)$$

In function 1, A and B are sets of targets separately. $d_{\left(a,b\right)}$ is the shortest path length between target a in network A and target b in network B. |A|and |B| denote the number of nodes in networks A and B separately. For each node a in network A, its shortest path length to each node in herb B is calculated and the minimum one will be kept. The exact process is applied to network B. Finally, the minimum values of each node in both networks A and B are summed and averaged, indicating how closely these two networks interact. Here, A represents the targets of one ingredient, and B represents the targets of another ingredient. Here, smaller $d\left(X,Y\right)$ indicates strong interactions among PPI networks.

#### 2.2.2 Herb-herb interaction score

The distance between herb-herb combinations was calculated based on their ingredient-ingredient network, with the shortest distance as the edge weight. The center distance *d*^*CC*^ between two herbs is the average shortest distance of their center ingredients:

(2) $$\mathrm{Center}:\;\langle d_{\mathrm{AB}}^{\mathrm{cc}}\rangle =d\left({\mathrm{centre}}_{A}+{\mathrm{centre}}_{B}\right)\;$$

(3) $${\mathrm{centre}}_{B}={\mathrm{argmin}}_{u\in B}\sum_{b\in B}d\left(b,u\right)$$

where B is the subnetwork covering all the ingredients in one herb. $d\left(b,u\right)$ represents the shortest path of each pairwise ingredient within a herb. The central ingredient is the one with the minimum sum distance to other ingredients. The shortest distance *d*^*S*^ between two ingredients is defined as follows:

(4) $$\mathrm{Shortset}:\langle d_{\mathrm{ab}}^{S}\rangle =\frac{1}{|a|+|b|}\sum_{a^{,}\in a,b^{,}\in b}d\left(a\prime ,b^{,}\right)\;$$

where $a^{,}$ and $b^{,}$ are the targets from ingredient a and b separately. The product of |a| and |b| represents the number of targets for ingredients a and b. Finally, the shortest 〈a,b〉 is the sum of all shortest path lengths between the two target sets, averaged to provide a measure of network proximity.

To determine the statistical significance, the null distribution was calculated using 1000 random ingredient pairs. Here, we performed a one-tailed Fisher's Z-test for each pair using the formula: $Z=(x-\mu_{n\mathrm{ull}})/\sigma_{\mathrm{null}}$, where x is the observed proximity score, $\mu_{\mathrm{null}}$ is the mean of the null distribution, and $\sigma_{\mathrm{null}}$ is the standard deviation of the null distribution. Pairs with *P* < 0.05 were considered statistically significant. It is essentially the proportion of the Z distribution cut off at the observed Z score. In notation, this is expressed as:

(5) $$p\left(x_{0}\right)=\mathrm{Pr}\left(d\left(X\right)>d\left(x_{0}\right);H_{0}\right)\;$$

where $x_{0}$ is the observed data ${(x}_{1,\;}x_{2},\;\ldots ,\;x_{n}$), d is a special function (statistic, e.g. a Z-score function), and X is a random sample ${(x}_{1,\;}x_{2},\;\ldots ,x_{n}$) from the sampling distribution of the null hypothesis. Therefore, in this study, we also used all co-occurring pairs in formulae as the null distribution for high-throughput screening of herb-herb and ingredient-ingredient combination recommendations in the browser function, comprising 61,757 herb-herb and 304,992 ingredient-ingredient pairs, due to computational constraints (exhaustive testing of 2.5 billion pairs was infeasible). For disease associations, we generated null distributions from 1000 randomly sampled pairs per test. Similarly, the interaction scores between an herb-herb pair are determined based on their ingredient-ingredient network.

### 2.3 Network-based inference of herb-disease indications

To further predict the disease indication of herbs, the network proximity method was employed to determine the association scores.

#### 2.3.1 Ingredient-disease association score

To measure the association between ingredients and diseases, we applied the z-score method. This network module method evaluates the relatedness between one drug and one disease. The shortest path length between drug *X* and disease *Y* is defined as:

(6) $$d\left(X,Y\right)=\frac{1}{\left|Y\right|}\sum_{y\in Y}\min_{x\in X}d\left(x,y\right)\;$$

$d\left(x,y\right)$ is the shortest path length between target node x in drug X and target node y in disease Y. The minimum values across all nodes in disease Y are summed and averaged, similar to the closest distance algorithm but in one direction from Y to X.

#### 2.3.2 Herb-disease association score

Similar to ingredient-disease associations, we combined all the targets of ingredients in one herb as the target set for one herb. Denoting that $C_{\mathrm{hA}}=(C_{1},C_{2},\;\ldots ,\mathrm{Ci})$ is a set ingredient in herb A. $C_{\mathrm{hB}}=(C_{1},C_{2},\;\ldots ,\mathrm{Ci})$ is a set ingredient in an herb B. $T_{C}=(T_{1},T_{2},\;\ldots ,\mathrm{Ti})$ is a set of targets in one compound. Then, the target for one herb can be denoted as:

(7) $$T_{H}=T_{C1}\cup T_{C\ldots }\cup T_{\mathrm{Ci}}\;$$

$T_{H}\;$is the union of targets of ingredients in this herb X. We extracted disease-related genes Y. The herb-disease proximity was also calculated.

For ingredient-disease associations**,** the shortest path length $d\left(X,Y\right)\;$between drug X and disease Y is defined:

(8) $$d\left(X,Y\right)=\frac{1}{\left|Y\right|}\sum_{y\in Y}\min_{x\in X}d(x,y)\;$$

Here, $d_{\left(x,y\right)}$ represents the shortest path length between a target node x in drug X and target node y in disease Y. The null distribution of $d\left(X,Y\right)$ was determined by 1000 random drug-disease pairs, based on which a *P* < 0.05 was considered significant. Similarly, for herb-disease associations, we combined all the targets of the ingredients within herbs to form a target set for that herb.

### 2.4 Technical implementation

HerbComb is implemented as a web-based platform with a client-server architecture. The frontend is built with Vue.js and Element UI, supporting both English and Chinese through Vue I18n. The backend utilizes Java and Spring Boot frameworks to deliver RESTful APIs and real-time services. Data is stored in MySQL (version 8.0) and cached in Redis (version 6.0). The web server runs on nginx (version 1.18) on TencentOS 5.4. Data processing pipelines were developed in Python (using Pandas and NetworkX) to integrate and clean data from multiple sources. Core entity data (diseases/conditions, herbs, ingredients, and targets) is available for download as CSV and JSON files from the website. In contrast, association data (herb-target, ingredient-target, herb-herb) can be exported per query through the interface. All data is released under a CC BY 4.0 license, permitting free download, reuse, modification, and redistribution, as well as commercial use.

Currently, HerbComb database is publicly accessible at https://herbcomb.com. The underlying data is updated quarterly (every three months), with versioned releases documented on the website. For data interoperability, the database supports cross-referencing with major biomedical resources through standardized identifiers, including OMIM for diseases, HPO for phenotypes, MeSH for medical headings, and ICD-10-CM for clinical diagnoses.

### 2.5 Experimental validation

#### 2.5.1 Materials

The mouse hippocampal neuron cell line HT22 cells were obtained from the Type Culture Collection of the Chinese Academy of Sciences (Shanghai, China). DMEM medium, Cell Counting Kit-8 (Yeasen, China), Oleanolic acid (OA), and Ferulic acid (FA) were purchased from Shanghai Baihua Biotechnology Co., Ltd. Fetal bovine serum (FBS) was purchased from VivaCell Biosciences (Shanghai, China).

#### 2.5.2 Cell culture and establishment of the oxygen and glucose deprivation (OGD) cell model

HT22 cells were maintained in a culture medium containing 90 % DMEM, 10 % FBS, 100 U/mL penicillin, and 100 μg/mL streptomycin. The incubator was set to 37°C with an atmosphere of 95 % O2 and 5 % CO2. The medium was replaced daily, and the cells were subcultured three times a week at a split ratio of 1:3. For the OGD treatment, the cells were placed in glucose-free DMEM and transferred to a hypoxic chamber with 95 % N2 and 5 % CO2 for four hours.

#### 2.5.3 Experimental protocols and treatment

The cells were divided into the following groups: Control group (CN); OGD group; OGD + 19.42 μg/mL FA; OGD + 38.84 μg/mL FA; OGD + 2.28 μg/mL OA; OGD + 4.57 μg/mL OA; OGD + 19.42 μg/mL FA + 2.28 μg/mL OA; OGD + 38.84 μg/mL FA + 4.57 μg/mL OA. The concentrations of FA and OA were selected based on previous studies by Mei [46] et al., Salau [47] et al., and Wang [48] et al., and were used to treat HT22 cells. FA and OA were dissolved in dimethyl sulfoxide (DMSO), and subsequently added to the HT22 cells at the indicated concentrations 3 h before model construction. The final concentration of DMSO in the medium was kept below 0.1 %.

#### 2.5.4 Evaluation of cell viability

Cell viability was assessed using the CCK-8 kit (Yeasen, China). HT22 cells were seeded in 24-well plates, and after treatment, 10 μL of the CCK-8 solution was added to each well and incubated at 37 ℃ for one h. The optical density (OD) was measured at 450 nm using a microplate reader (Thermo Fisher Scientific). Cell viability in each treatment group was calculated by normalizing the OD values to the control group, which was set as 100 %.

## 3 Results

### 3.1 Overview of HerbComb database

**Fig. 1:**
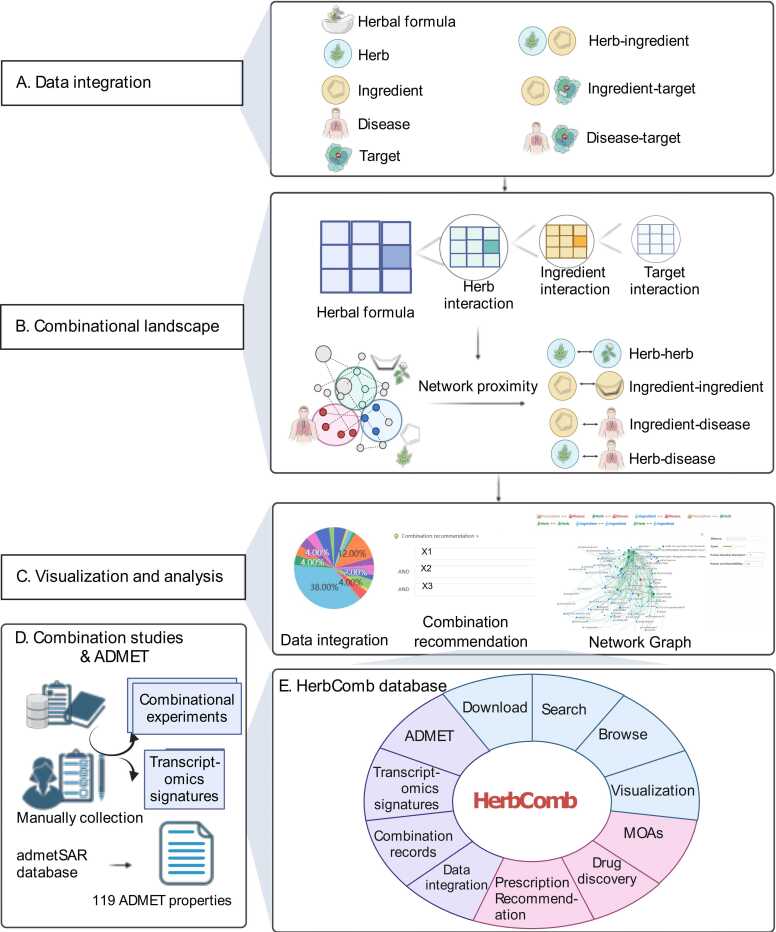
The overview of the HerbComb database. (A) HerbComb integrates formula-herb-ingredient-target associations, protein-protein interactions, and disease-gene associations, covering 851,409 ingredient-disease pairs (839 diseases and 1016 ingredients), 3053,258 herb-disease pairs (838 diseases and 3644 herbs), 304,992 ingredient-ingredient pairs and 61,757 herb-herb pairs that co-occur in 46,929 TCM formulae. (B) A network proximity model was constructed to characterize the interactions between herbs, diseases, ingredients, and their combinations. Downstream analysis of the synergistic interactions was provided, effectively capturing the unique combinatorial characteristics of herbal medicines. (C) Customized combinational analysis: HerbComb supports tailored analyses between ingredients, herbs, TCM prescriptions, and diseases through the "Combination Recommendation" button. This function mimics the prescription process in TCM clinical practice. (D) Additionally, experimental data about herbal combinations and transcriptomics signatures were manually curated from the literature, and ADMET properties were systematically calculated. (E) HerbComb is a versatile data exploration platform designed to characterize synergistic interactions among herbal medicines, enhancing the understanding of synergistic mechanisms and facilitating the discovery of effective drug combinations for disease treatment.

**Fig. 2:**
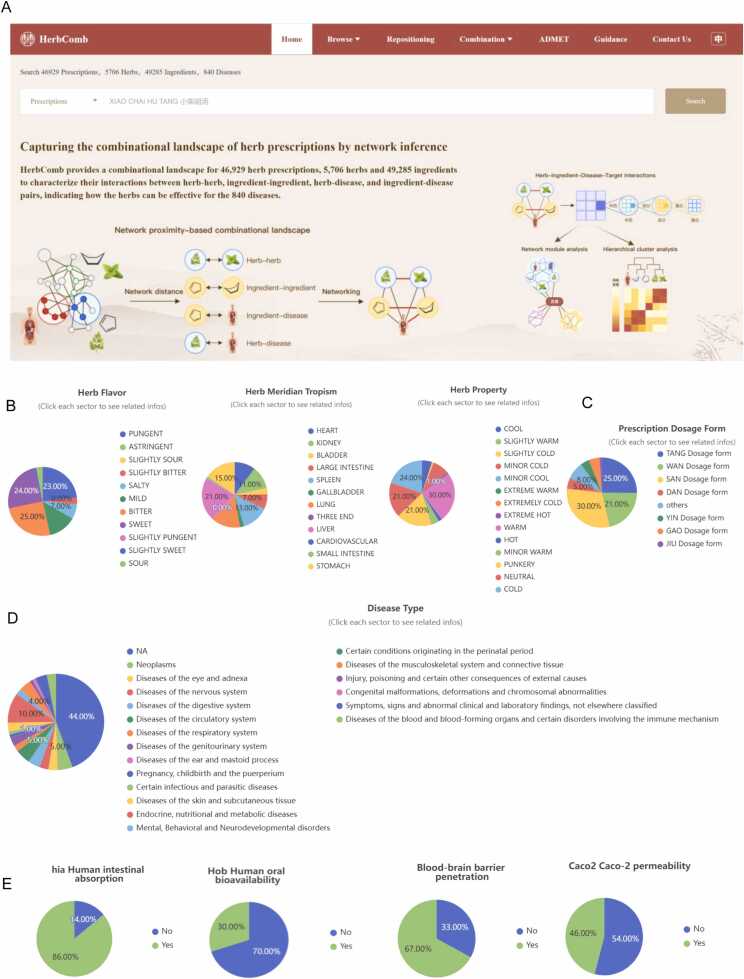
Data statistics of HerbComb. (A) Home page of HerbComb. (B) Distribution of flavors, meridians, and properties for herbs in HerbComb. (C) Distribution of dosage forms of prescriptions in HerbComb. (D) Distribution of disease types contained in HerbComb. (E) Distribution of ADMET properties for ingredients in HerbComb.

HerbComb was designed to facilitate the identification of synergistic interactions among herbal components. To achieve this goal, various available information on TCM was integrated into the HerbComb database, encompassing 46,929 formulas, 5706 herbs, and 49,285 ingredients. Additionally, to provide a comprehensive understanding of the mechanisms of action and disease indications, the database includes 25,212 disease-associated genes, 162,299 herb-ingredient pairs, 348,543 ingredient-target pairs, and 325,212 disease-target pairs, all organized into a multi-level heterogeneous network (Fig. 1 and Fig. 2B-D). Based on these prior acknowledgments, we employed network-based methodologies to systematically characterize interactions among ingredients, thereby constructing a comprehensive combinatorial landscape for herbal medicine (Fig. 1 and Fig. 2A).

HerbComb offers the following four innovative functionalities **(**Fig. 1**)**, including: (i) the identification of potential therapeutic and synergistic herbal components for a wide range of diseases; (ii) interactive analysis of the underlying mechanisms of action for specific herbal formulations; (iii) a decision-support tool to predict the therapeutic effects of a given herbal formula, emulating the prescription process of TCM; and (iv) the integration of pharmacotranscriptomics, pharmacokinetics, and toxicity profiles, facilitating a systems-level characterization of herbal medicine.

### 3.2 Screen therapeutic herbs and ingredients with synergistic effects

HerbComb employs systematic modeling to identify potential therapeutic and synergistic ingredients or herbs for specific diseases [49]. By clicking the "Repositioning" button, users can browse all potential therapeutic herbs and ingredients for 840 diseases (Figure S1).

**Fig. 3:**
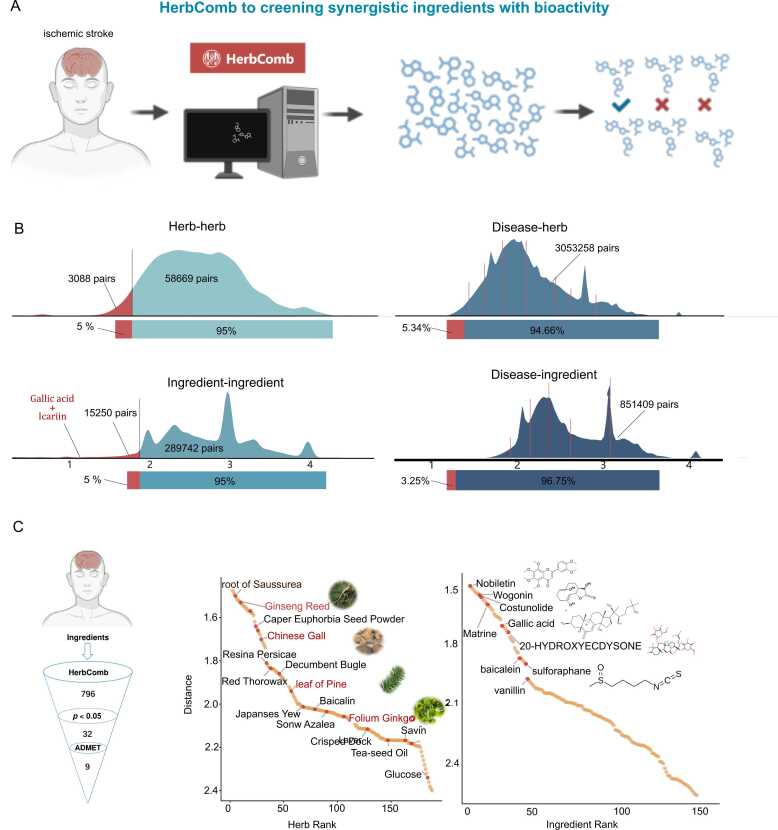
Large-Scale screening results of therapeutic herbs or ingredients for 840 Diseases. (A) Concepts of drug repurposing function: HerbComb introduces a drug repurposing feature to identify potential therapeutic applications of herbs and ingredients across a wide range of diseases. (B) Statistical analysis of significant associations: The HerbComb platform offers statistical insights into the significant relationships between herb-herb, herb-disease, ingredient-ingredient, and ingredient-disease pairs, facilitating a deeper understanding of their interactions. (C) Drug screening for ischemic stroke: Using ischemic stroke as an example, HerbComb screens and prioritizes top therapeutic herbs and ingredients, which are then visualized for further analysis.

Specifically, we constructed a substantial combinational atlas, covering 851,409 ingredient-disease pairs (839 diseases and 1016 ingredients), 3053,258 herb-disease pairs (838 diseases and 3644 herbs), 304,992 ingredient-ingredient pairs, and 61,757 herb-herb pairs that co-occur in 46,929 TCM formulae. Consequently, HerbComb identified 2999 high-confidence synergistic herb-herb pairs, 7748 ingredient-ingredient interactions, 179,461 therapeutic disease-herb associations, and 27,673 disease-ingredient associations across 840 diseases. This extensive resource significantly complements existing databases (Fig. 3A).

On average, each disease is associated with four herbs and one ingredient (Fig. 3B), highlighting the potential of herbal medicines as a valuable resource for drug discovery. Additionally, HerbComb serves as a comprehensive data portal for uncovering therapeutic and synergistic ingredients within TCM formulas. On average, six herbs and 199 ingredients in each herbal formula are significantly associated with specific diseases.

Neurological disorder Stroke is an area where herbal medicines have demonstrated promise [50], [51]. Using the "Repositioning" function in HerbComb, we screened potential herbal drugs and ingredients for stroke as an example. Consequently, 32 ingredients were prioritized as statistically significant (*P* < 0.05) for ischemic stroke **(**Fig. 3**C)**. Furthermore, when searching for a particular herb or ingredient, the top-scoring diseases will be provided as potential novel indications for which that herb/ingredient may have therapeutic efficiency. Among these repurposed herbs, *Ginseng Reed*, *Chinese Gall*, *leaf of Pine,* and *Folium Ginkgo* have been reported to be associated with ischemic stroke, underscoring the potential of HerbComb for drug discovery. Similarly, prioritized ingredients, such as Ginsenoside RG1 [52], Ginsenoside Rg3, ferulic acid, and Cryptotansl have also been reported for ischemic stroke treatment.

### 3.3 HerbComb offers a comprehensive platform for combinatorial analysis

**Fig. 4:**
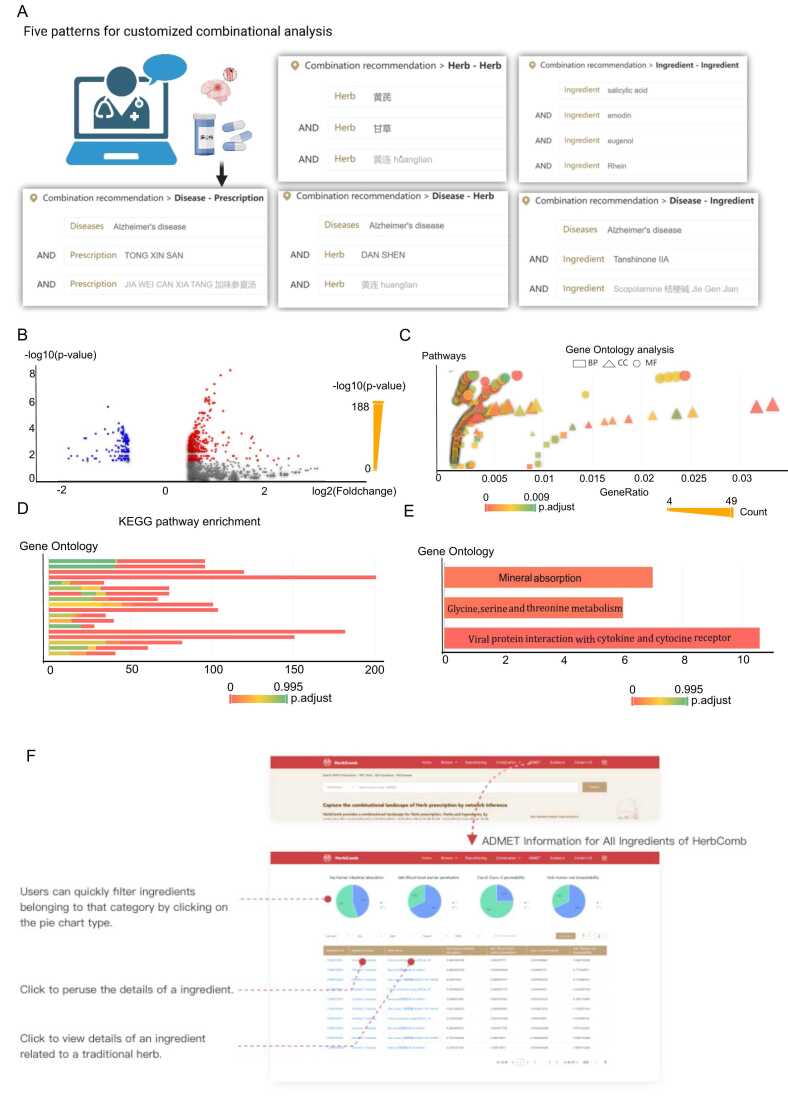
Additional functions of HerbComb to enhance combinational analysis. (A) HerbComb enables tailored analyses between ingredients, herbs, prescriptions, and diseases through the "Combination Recommendation" feature. This function supports five combination patterns: a) “herb + herb + disease”, b) “ingredient + ingredient + disease”, c) “prescription + prescription + disease”, d) “herb + herb”, and e) “ingredient + ingredient”. (B-D) Transcriptomics signature analysis for determining synergistic mechanisms. HerbComb integrates transcriptomics data to uncover the molecular mechanisms underlying synergistic interactions, providing deeper insights into how herb and ingredient combinations exert their effects. (E) Enriched pathways of Ginsenoside Rg1 in the treatment of stroke by transcriptomics signature analysis. (F) HerbComb enables the systematic evaluation of ADMET (Absorption, Distribution, Metabolism, Excretion, and Toxicity) properties for ingredients in herbal medicines, facilitating more efficient drug screening processes.

HerbComb offers a comprehensive platform for combinatorial analysis, providing interaction scores for millions of herb-ingredient pairings (Fig. 1B). The platform features six main functions designed to facilitate in-depth exploration and analysis:

1) Herb-specific synergistic interactions: When users search for a specific herb or a prescription, HerbComb returns significant synergistic ingredient-ingredient interactions within that herb or prescription.
2) Herb-Herb combinations and disease associations: The HerbComb platform provides all potential herb-herb combinations and diseases associated with the searched herb.
3) Ingredient-Ingredient combinations and disease associations: HerbComb provides all potential ingredient-ingredient combinations and diseases associated with the searched ingredient.
4) Visualization of synergistic pairs: These significantly associated pairs can be visualized in a comprehensive combination network, enabling downstream analyses such as modularity and ADMET filtering.
5) Customized combinational analysis: HerbComb supports tailored analyses between ingredients, herbs, TCM prescriptions, and diseases through the "Combinational Recommendation" button. This function mimics the prescription process in TCM clinical practice. In total, five combination patterns are supported in the "Combinational Recommendation" function (Fig. 4A): “herb + herb + disease,” “ingredient + ingredient + disease,” “prescription + prescription + disease,” “herb + herb,” and “ingredient + ingredient.” For example, users can query the therapeutic potential of a specific herb combination for treating a particular disease (i.e., the “herb + herb + disease” query pattern). After inputting the herbs and a disease, a combinatorial landscape is visualized, highlighting interactions among herb-herb, ingredient-ingredient, herb-disease, and ingredient-disease pairs (Fig. 1C).

In addition to predicted combinations from network modeling, HerbComb provides curated data on experimentally validated herbal or ingredient combinations. Systematically curating around 4000 publications offers comprehensive information on 79 herb-drug, 98 ingredient-drug, 84 herb-herb, 76 ingredient-ingredient, and 14 ingredient-herb interactions, filling a critical gap and serving as a foundational resource for computational models. With these data, HerbComb is particularly valuable for experimentally validated combinations whose molecular synergistic mechanisms remain unclear, as it supports in-depth combinatorial landscape analysis, enhancing our understanding of known synergistic pairs. For example, Icariin and Gallic acid have been reported to exhibit synergistic effects in Alzheimer's disease [53]. Using the "Combinational Recommendation" function, HerbComb revealed that these two compounds exhibit stronger interactions and are significantly associated with Alzheimer's disease (Fig. 3B). As shown in Fig. 3B-C, the combination of Icariin and Gallic acid ranks 2306 across all pairs. Additionally, the distance between Icariin and AD is 2.03 (*P* = 0.03), while the distance between Gallic acid and AD is 1.97 (*P* = 0.03). This suggests that Icariin and Gallic acid may have synergistic therapeutic effects. While our model identifies statistically significant compound-disease associations through network proximity, these represent potential mechanistic relationships rather than confirmed treatment effects. Clinical validation remains essential, particularly since network proximity may capture both therapeutic and pathophysiological associations. It was reported that *Epimedium* aqueous extract, which contains Icariin as a major active constituent, ameliorates cerebral ischemia/reperfusion injury through inhibiting ROS/NLRP3-mediated pyroptosis [54]. Moreover, Rosemary (*Rosmarinus officinalis* L.), which contains Gallic acid, exhibits neuroprotective effects by enhancing cerebral ischemic tolerance in experimental stroke [55]. Similarly, green tea, a natural product rich in Gallic acid, also shows potential therapeutic effects against ischemic stroke [56]. This finding highlights the potential of HerbComb to uncover and elucidate the mechanisms of action (MOA) underlying experimental combinatorial results.

### 3.4 Transcriptomics signature analysis for synergistic mechanisms determination

Typically, target information for most herbs is predicted using computational tools but often lacks experimental validation, which can introduce noise. Recently, transcriptomic changes induced by herbal medicines, referred to as gene expression signatures, have shown promise for elucidating the holistic effects of specific herbs or ingredients [57], [58]. Therefore, the HerbComb database provides gene expression changes induced by herb perturbations pre- and post-treatment, establishing differentially expressed genes (DEGs) as transcriptomic signatures (Fig. 1D). These signatures enable the identification of molecular pathways influenced by the treatment, facilitating a deeper understanding of the pharmacological mechanisms underlying herbal therapies.

HerbComb has now integrated data from various herbal perturbations to generate gene expression signatures for 693 herbal treatments. It also offers tools to analyze and visualize DEGs, pathway enrichment, and Gene Ontology (GO) terms (Fig. 4B-D), thereby enhancing the understanding of transcript-level effects of herb or ingredient perturbations. These gene signatures serve as a critical complementary dataset for analyzing herbal combinations, aiding in identifying synergistic mechanisms [59].

For instance, although Ginsenoside Rg1 has been prioritized for ischemic stroke by the repositioning function of HerbComb (Fig. 3C), the underlying therapeutic mechanism remains unknown. Therefore, transcriptomics signature analysis was also supported by HerbComb to facilitate further exploration of synergistic mechanisms. In total, 24 signature genes were associated with Ginsenoside Rg1 in HerbComb, which were mainly enriched mineral absorption, glycine, serine, and threonine metabolism, and viral protein interaction with cytokine and cytokine receptors (*P* < 0.05, Fig. 4E). Intriguingly, Ginsenoside Rg1 has been reported to regulate cytokines through the viral protein interaction with cytokine and cytokine receptor pathway [60], thereby underscoring HerbComb’s potential to provide mechanistic insights into herbal treatments.

### 3.5 ADMET properties to facilitate the identification of synergistic ingredient

Studying ADMET properties is crucial for understanding the pharmacokinetics and pharmacodynamics of herbal medicine. While existing databases provide some ADMET properties (e.g., TCMSP, YaTCM, ETCM, and TCMID), they often lack critical information, particularly on toxicity. HerbComb addresses this gap by integrating the most up-to-date set of 119 ADMET properties for 49,285 ingredients, encompassing 18 physicochemical properties, 43 human health toxicity endpoints, 16 environmental risk assessment endpoints, and nine cosmetic risk assessment endpoints (Figs. 1D and 4F). This comprehensive ADMET information is invaluable for identifying synergistic ingredients with high drug-likeness and low toxicity.

As shown in Fig. 2E, approximately 86 % of the 9783 ingredients exhibit optimal human intestinal absorption, while only 30 % demonstrate good oral bioavailability. Furthermore, around 67 % of the ingredients can pass the blood-brain barrier. HerbComb also provided a list of 18,314 ingredients (36.55 %) that may exhibit hepatotoxic effects for an alert.

HerbComb enables users to manually filter ingredients based on ADMET properties when conducting combinational analysis, thereby facilitating the process. For instance, among the 32 ingredients prioritized for ischemic stroke, only nine ingredients are significant for ischemic stroke and have good human intestinal absorption and human oral bioavailability > 30 % with permanent BBB (Fig. 3C).

### 3.6 Sensitivity analysis

To gain a deep understanding of the entire database, we conducted a distance distribution analysis to identify popular herbs and ingredients, as well as diseases that are overrepresented.

**Fig. 5:**
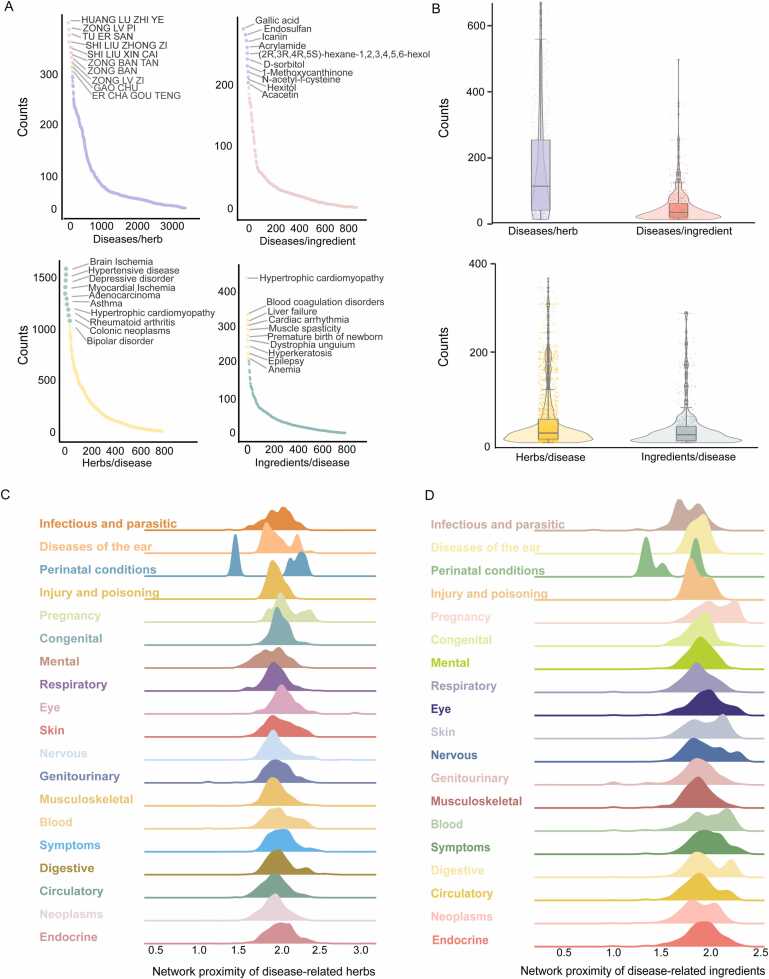
Distance distribution analysis. (A-B) Distribution of the number of associated diseases per herb, diseases per ingredient, herbs per disease, and ingredients per disease. (C-D) The density of network proximity values for significant herb-disease associations (C) and ingredient-disease associations (D) across 19 therapeutic classes of diseases.

We conducted a sensitivity analysis to investigate the distribution of ingredients, diseases, and herbs, identifying hub herbs with a significantly larger number of pairs than others. As shown in Fig. 5A-B below, the distribution of herbs, ingredients, diseases, and their associated pairs is normalized. On average, a herbal formula contains six herbs and 199 ingredients, many of which are significantly related to certain diseases. These results suggest that herbal medicines may be valuable resources for drug discovery. Notably, we found that the top 10 hub herbs and ingredients are associated with more than 300 and 200 diseases, respectively. Similarly, the top 10 hub diseases are associated with ∼1000 herbs and ∼200 ingredients separately (Fig. 5A). On average, each disease is associated with 208 and 32.63 herbs and ingredients, while each herb and each ingredient are associated with 47.30 and 35.39 diseases, respectively (Fig. 5B). Diseases (n = 840) were classified into 19 therapeutic categories. Fig. 5C-D shows the distribution of significant herb-disease pairs and significant ingredient-disease pairs across these categories. All pairs of disease-herbs and disease-ingredients are unique, with deduplicated pairs removed. The number of herb-disease pairs per category ranges from 197 to 26,948, with diseases in the Endocrine, Nutritional, and Metabolic classification having the highest representation, at 26,948 related disease-herb pairs. These findings suggest that HerbComb is a valuable data portal for revealing the therapeutic and synergistic ingredients in the TCM formula.

### 3.7 Stratified analysis

Additionally, we performed stratified analysis by dividing the dataset into meaningful subgroups (called strata), such as herb popularity, disease category, and formula complexity. Stratified analysis helps detect potential differences or biases that might have been masked in the aggregated data.

**Fig. 6:**
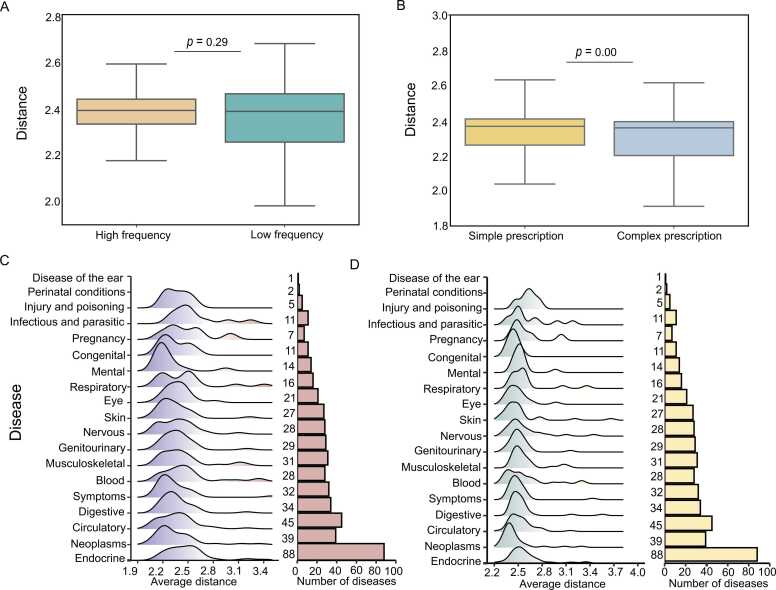
Stratified analysis of distance. (A) Distance comparison by classifying herbs into high and low frequency herbs by their median value in formulae. (B) Distance comparison by cutting the formula into two groups according to the herbs that comprise that formula: Simple prescription with $N\left(\mathrm{herbs}\right)<6$ and complex prescription with $N\left(\mathrm{herbs}\right)\geq 6$. (C-D) Network distance distributions between herbs/ingredients and diseases across 19 disease classifications. Ridge plots display distance distributions, while bar plots indicate the number of diseases in each category.

Firstly, we classified herbs into high- and low-frequency herbs based on their median value in the formulae. We found that higher-frequency herbs show a similar distance to those of lower-frequency herbs (Fig. 6A). Then, we divided the formula into two groups based on the herbs that comprise it: simple prescriptions with $N\left(\mathrm{herb}s\right)<6$ and complex prescriptions with $N\left(\mathrm{herb}s\right)\geq 6\;$. Notably, complex prescriptions show significantly closer distances than those simple ones (Fig. 6B). To characterize the relationship between herb/ingredient and different disease types, we analyzed the distribution of network distances across 19 disease classifications. The bar plot to the right illustrates the number of diseases in each category. We observed a similar distribution of distance, regardless of the number of diseases in the classification (Fig. 6C-D).

### 3.8 Case study

Using Stroke as a case study, we have identified a famous herbal formula, Tongxinluo, and found that Oleanolic acid and Ferulic acid are synergistic ingredients that have not been previously reported.

The TCM formula Tongxinluo Capsule (TXL) has been used clinically for the prevention and treatment of cardiovascular diseases, particularly ischemic stroke. However, existing research primarily focuses on its overall efficacy, with limited knowledge of the interactions between its ingredients. Using HerbComb, we determined the interaction distances between herbs and ingredients associated with ischemic stroke.

**Fig. 7:**
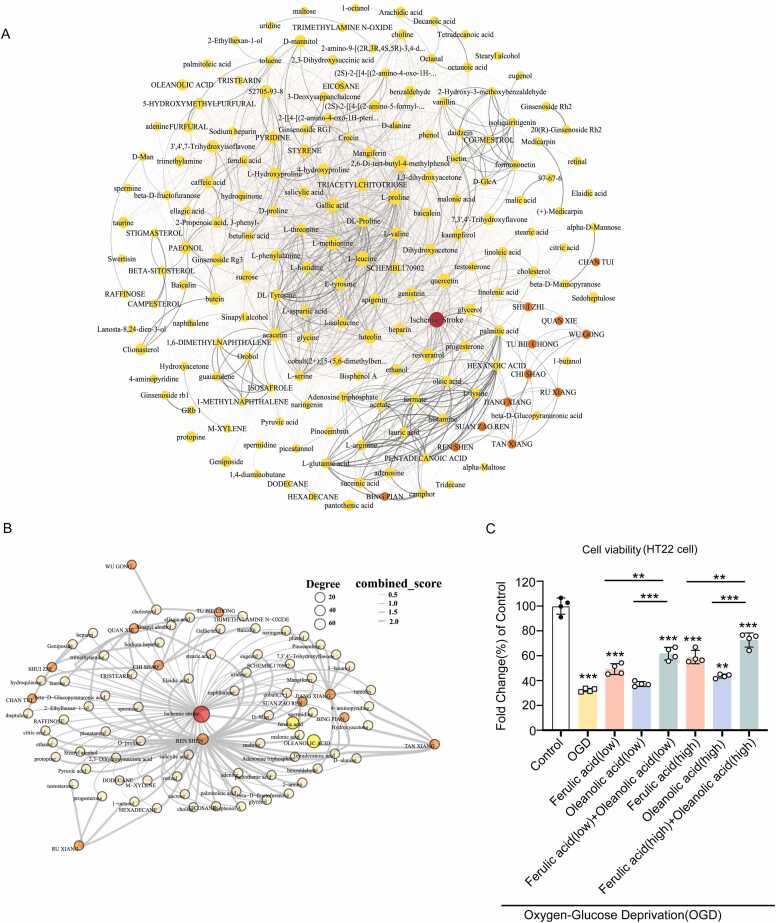
The combinatorial landscape of Tongxinluo Capsule. (A) Herb-Component-Disease Network. (B) Key disease-associated module via network community analysis. (C) The viability of HT22 cells in Oxygen and Glucose Deprivation (OGD) conditions of different concentrations of Oleanolic acid and Ferulic acid (T-test, * *P* < 0.05, *** *P* < 0.01, *** *P* < 0.001).

The combinational effects of TXL were further investigated by constructing a network to represent the interactions between ingredients and diseases (Fig. 7A). To identify key active components and potential synergistic ingredients, the Louvain algorithm was employed for community detection, assuming that ingredients clustered with the ischemic stroke node are more likely to exhibit synergistic effects. According to ingredient-disease and ingredient-ingredient distance analysis, Oleanolic acid and Ferulic acid were ultimately selected (Fig. 7B).

To validate the synergistic effects of Oleanolic acid and Ferulic acid, we conducted a cell viability assay on neural protection using the HT22 cell line (immortalized mouse hippocampal neuronal cells). We found that the combination of Oleanolic acid and Ferulic acid at both low and high concentrations shows significantly increased cell viability in the HT22 cell line (T-test, *P* < 0.01, Fig. 7**C**). These results suggest that the HerbComb database can be an effective platform for discovering synergistic ingredients and gaining deeper insights into the mechanisms of action of herbal medicines.

## 4 Discussion

Elucidating the synergistic mechanisms of herbal medicines presents a formidable scientific challenge, given their inherent multi-component nature and complex bio-molecular interactions. While traditional knowledge and empirical testing provide a foundational understanding, they are inherently limited in their ability to explore the vast combinatorial space of herbal ingredients systematically. Contemporary TCM databases have made strides in data aggregation, yet they predominantly remain confined to cataloguing ingredient targets and linking them to diseases for conventional pathway analysis. This paradigm often overlooks a critical pharmacological principle: herb-disease associations frequently arise from complex, system-level interactions that the simple additive effects of individual constituents cannot explain.

To bridge this gap, we developed HerbComb, a platform that addresses these limitations through high-throughput computational screening. By prioritizing the most promising herbal and ingredient pairs for experimental validation, our approach significantly reduces the reliance on traditional trial-and-error methods. Consequently, HerbComb serves not merely as a data portal but as an integrated analytical environment that supports sophisticated combinatorial analysis and drug repurposing. A core design principle is its function as a hypothesis-generating tool, offering a user-friendly interface that requires no programming expertise while enabling interactive visualization of complex, multidimensional pharmacological relationships.

To distinguish HerbComb from other existing TCM databases, we conducted a systematic comparison with 17 of the latest TCM databases published after 2017, which are presented in Table 1. Unlike existing TCM databases that primarily link ingredient targets to diseases for pathway enrichment and mechanism-of-action (MOA) analysis, the core methodology of HerbComb is a network-proximity-based inference model for drug combination, rigorously validated through permutation tests and cross-disease benchmarking [23]. This model quantifies interactions between herbs, ingredients, and diseases to predict synergy, moving beyond mere data aggregation. Recent studies have validated the efficacy of network-based synergy prediction [24], [25], [26], [27], supporting the validity of our strategy. To our knowledge, HerbComb is the first database to provide a comprehensive network of herb-herb, ingredient-ingredient, herb-disease, and ingredient-disease relationships. Importantly, it supports customized combinatorial analysis, which can aid in understanding the synergistic MOAs of herbal formulas in clinical practice. Furthermore, these complex landscapes can be further used for network module analysis. Therefore, HerbComb serves as a comprehensive interactive platform supporting diverse paradigms—such as drug repurposing, customized combinatorial analysis, and biological exploration—powered by its network-based synergy inference and combination modeling engine.

**Table 1:** Compare HerbComb with previous databases after 2017.

Compare HerbComb with previous databases after 2017								
NO.	TCM database	Published year (Ordered)	Source of Validated targets	Source of Predicted targets	Volume of pharmacological Data	Pharmacological analysis characteristics	Herb/ingedient-disease association	Combinational analysis
1	TCMKD	2024.09	/	Targets of ingredients from eight public databases and targets of diseases from publicly available resources, including MalaCards, DisGeNet, TTD, and CTD databases	262 syndromes, 48,644 formulas, 9953 Chinese patent drugs (CPDs), 3817 Chinese medicinal materials (CMMs), 36,403 ingredients, 18,665 targets, and 23,750 diseases.	1) The Jaccard similarity score was calculated to assess potential therapeutic parallels among various formulas 2) Association between ingredients and diseases: using the hypergeometric distribution test 3) Network localization and separation: identify the locations of drug-disease modules within the human protein interactome and predict drug-disease relationships by calculating the distance between drug-disease modules.	√	/
2	TCMM	2024.04	/	TCMM aggregates knowledge from six leading databases across the domains of TCM and modern medicine, which are PrimeKG, TCMBank, PharMeBINet, SymMap,and TCMID.	knowledge network, including 248,434 entities and 3447,023 relational triplets.	Rule based knowledge correction: TCM Knowledge Discovery Model is employed to represent relations between entities within predefined complex query paths. Prescription Repositioning: the complex query path 2) Symptom Related Target Prediction 3) Prescription Generation	√	/
3	HERB.20	2024.01	/	Fisher’s exact test followed by BH multiple test correction	/	Indirect relationship between ingredient and disease: gene targets as the middle component with reliable associations (FDR < 0.01) selected as the final statistical inference set. Herb–target and herb–disease relationships: using ingredients and targets as the middle components, respectively. 3) Formula–ingredient, formula–target and formula–disease relationships: using herbs, herbs, and gene targets as the middle components	/	
4	BATMAN-TCM2.0	2023.08	Known TTI datasets from multiple published databases, including the Kyoto Encyclopedia of Genes and Genomes (KEGG), DrugBank the Therapeutic Target Database (TTD), HIT and HERB text mining and manual curation following a keyword co-occurrence protocol	2 319 272 putative TTIs with high confidence, based on the similarity algorithm we developed in BATMAN-TCM 1.0	624 syndromes, 133,518 prescriptions, 8073 diseases (including 1843 TCM-specific diseases), 8259 Chinese herbal medicines, 43,413 ingredients, 17,602 targets, and 8182 drugs	1) Screening active TCM ingredients based on disease-specific signatures 2) A uniform confidence scoring system to rank predicted ingredient–target protein interaction	√	/
5	ETCM v2.0	2023.03	/	1) two-dimensional ligand similarity search module in our indoor D3CARP platform 2) the BindingDB database	48,442 TCM formulas recorded by ancient Chinese medical books, 9872 Chinese patent drugs, 2079 Chinese medicinal materials and 38,298 ingredients	1) Manually collected a total of 14 direct associations: “TCM Formula/Chinese Patent Drug-Herb”, “TCM Formula/Chinese Patent Drug-Disease”, etc. 2) Jaccard similarity scores: identify prescriptions/herbs with similar clinical efficacy, to summarize the rules of prescription use, and to find alternative herbs for endangered Chinese medicinal materials 3) Target identification and prediction 4) Drug similarity evaluation results in each detailed information page 5) Improved systematic analysis function： Examples of various networks constructed by the Systematic Analysis tool of ETCM v2.0. 6) Advanced Systematic Analysis tool: filter the components of the network to be constructed by various features in the Advanced Systematic Analysis tool.	√	/
6	TCMSID	2022.12	ChEMBL	metaTARFISHER: Assembling different target prediction tools including SEA, SwissTargetPrediction, HitpickV2, PPB, PPB2 and ChEMBL.	499 herbs registered in the Chinese pharmacopeia with 20,015 ingredients, 3270 targets	Ingredient structural reliability evaluation 2) Mechanism exploration of TCM herbs: key ingredients filtering and target identification, mechanism of action of herbal ingredients can be inferred according to the multilevel herb-ingredient-target-drug network 3) Ingredient-related drug information: therapeutic effects and known targets of the drugs being connected, to bridge the gap between TCM herbs and modern drugs through chemical similarity calculation.	√	/
7	TCMBank	2023.10	/	/	9192 herbs, 61,966 ingredients (unduplicated), 15,179 targets, 32,529 diseases, and their pairwise Brelationships	Ensemble learning-based drug discovery protocol: identifying potential leads and drug repurposing 2) Standard information on targets and diseases: intelligent recognition of published references and books 3) Intelligently identify newly published references and continuously provide the latest TCM-related information	/	/
8	ITCM	2023.01	/	/	high-through put experiments and constructed the largest data repository of 496 representative active ingredients,	six state-of-art signature search methods: screen active ingredients and determine the optimal signature size for all methods	√	/
9	TCMSSD	2023.01	/	/	624 syndromes, 133,518 prescriptions, 8073 diseases (including 1843 TCM-specific diseases), 8259 Chinese herbal medicines, 43,413 ingredients, 17,602 targets, and 8182 drugs	Syndrome identification tool: a deep learning approach to construct the knowledge graph and utilize the BM25 algorithm for syndrome prediction.	/	/
10	LTM-TCM	2022.03	information was integrated from the BATMAN-TCM (http://bionet.ncpsb.org/batman- tcm/), ChEMBL (https://www.ebi.ac.uk/chembl/) and STITCH (http://stitch.embl.de) databases.	Ultimate prediction score calculated by a supervised model with eight features (ATC-GO, FP2-close, STITCH-sequence, expression-close, ATC-sequence, functional group-sequence, functional group-GO, and side effect-sequence, and using the maximum of the likelihood ratios (LRs))	1928 symptoms, 48,126 prescriptions, 9122 plants, 34,967 ingredients, 13,109 targets and 1170,133 interactions	Personalized computational pipe­lines to explore the mechanism of TCM symptoms 1) Autoseed is used for symptom-related genes and clusterProfiler for potential pathways 2) Prescription similarity analysis: identify the most similar prescription associated with the searching prescription by constructing the composed herb matrix of all pairwise prescriptions and calculating their Pearson cor­relation coefficient. 3) Highly frequent herb-pair combination analysis for a specific symptom: summarizing the binary and triple herb-pairs in all symptom-related prescriptions, reflect treatment prevalence. 4) SwissADME: predict the ingredients’ ADME properties and Autofrequent herb-pair combination analysis for a specific symptom, by summarizing the binary and triple herb-pairs in all symptom-related prescriptions, reflecting the frequency of herb-pair combinations in the medication expe­rience and the Dock Vina for virtual screening of ingredient-related targets. 5) To discover new indications: use BioNLP to divide the prescriptions’ indications and functions into words and predict disease-related symp­toms. based on the NER approach (see in methods BioNLP), different types of re­lationships including “prescription-herb”, “herb-ingredient”, and “ingredient-target” is recognized and virtualized as a knowledge graph. 6) To suggest innovative formulas: use three pipelines- pre­criptions similarity analysis, herb pair analysis, and intelligent prescription suggestion.	√	/
11	SuperTCM	2021.01	human targets for herbal ingredients were taken from ChEMBL_27	/	6516 TCM drugs (or “herbs”) with 5372 botanical species, 55,772 active ingredients against 543 targets in 254 KEGG pathways associated with 8634 diseases.	Ingredient-target-pathway relationships: projected their ingredients and targets onto KEGG Global Maps which were redesigned in iPath3.0, an interactive explorer of pathways that allows users to easily navigate and explore the complex pathway maps Ingredient-target-disease relationships: The diseases are coded via the ICD−10-CM system and are linked to herbs and recipes according to their targets of herbs and recipes.	√	/
12	TCMIO	2020.04	ChEMBL	Balanced substructure-drug-target network-based inference (bSDTNBI）	/	Ontology function KEGG biological pathway	√	/
13	YaTCM	2018.11	/	multi-voting chemical similarity ensemble approach	/	KEGG pathway enrichment	√	/
14	TCMAnalyzer	2018.01	ChEMBL	/	/	Identify the potential compounds for the bioactivities for a TCM herb through scaffold−activity relation searches using structural search techniques, Investigate the molecular mechanism of action for a TCM herb at the systemic level: ingredient-target-disease linking Explore potentially targeted bioactive herbs.	√	/
15	TCM-Mesh	2017.06	STITCH	/		ingredient-target-disease linking		/
16	TCMID2.0	2017.10	/	/	18,203 herbal ingredients, 15 prescriptions, 82 related targets, 1356 drugs, 842 diseases	ingredient-target-disease linking	√	/
17	TCMSP	2014.04	HIT database	SysDT model	84,260 ingredient-target pairs, (7947 ingredients, 1079 targets)	ingredient-target-disease linking	√	/
18	HerbComb	/	/	/	46,929 formulas, 5706 herbs, and 49,285 ingredients, 25,212 disease-associated genes, 162,299 herb-ingredient pairs, 348,543 ingredient-target pairs, and 325,212 disease-target pairs, identified 2999 high-confidence synergistic herb-herb pairs, 7748 ingredient-ingredient interactions, 179,461 therapeutic-herb associations, and 27,673 disease-ingredient associations across 840 diseases. (unique)	1) Herb-specific synergistic interactions: When users search for a specific herb for or prescription, HerbComb returns significant synergistic ingredient-ingredient interactions within that herb or prescription. (unique) 2) Herb-Herb combinations and disease associations: The HerbComb platform provides all potential herb-herb combinations and diseases associated with the searched herb. (unique) 3) Ingredient-Ingredient combinations and disease associations: HerbComb provides all potential ingredient-ingredient combinations and diseases associated with the searched ingredient. (unique) 4) Visualization of synergistic pairs: These significantly associated pairs can be visualized in a comprehensive combination network, enabling downstream analyses such as modularity and ADMET filtering. (unique) 5) Customized combinational analysis: HerbComb supports tailored analyses between ingredients, herbs, TCM prescriptions, and diseases through the "Combinational Recommendation" button. A decision-support tool to predict the therapeutic effects of a given herbal formula, emulating the prescription process of TCM. (unique)	√	√(unique)

Specifically, for the drug discovery field, it allows researchers: 1) to identify novel synergistic pairs systematically; It can proactively suggest new herb/ingredient combinations for a given disease based on network topology, moving beyond the analysis of only historically documented pairs; 2) generate mechanistic hypotheses for known combinations: For experimentally validated combinations with unknown mechanisms. HerbComb can provide a mechanistic context by revealing their shared network proximity to the disease and to each other; 3) Prioritize repurposing candidates with pharmacokinetic filters: It uniquely combines synergy predictions with ADMET profiling, allowing for the prioritization of candidates that are not only potentially synergistic but also possess drug-like properties; 4) Leverage transcriptomic signatures for p mechanistic exploration: The integrated gene expression data enables users to move from predicted targets to observed pathway perturbations, offering a direct window into the transcriptional programs modulated by herbal treatments and thus a deeper layer of pharmacological insight not typically available in other databases.

**Fig. 8:**
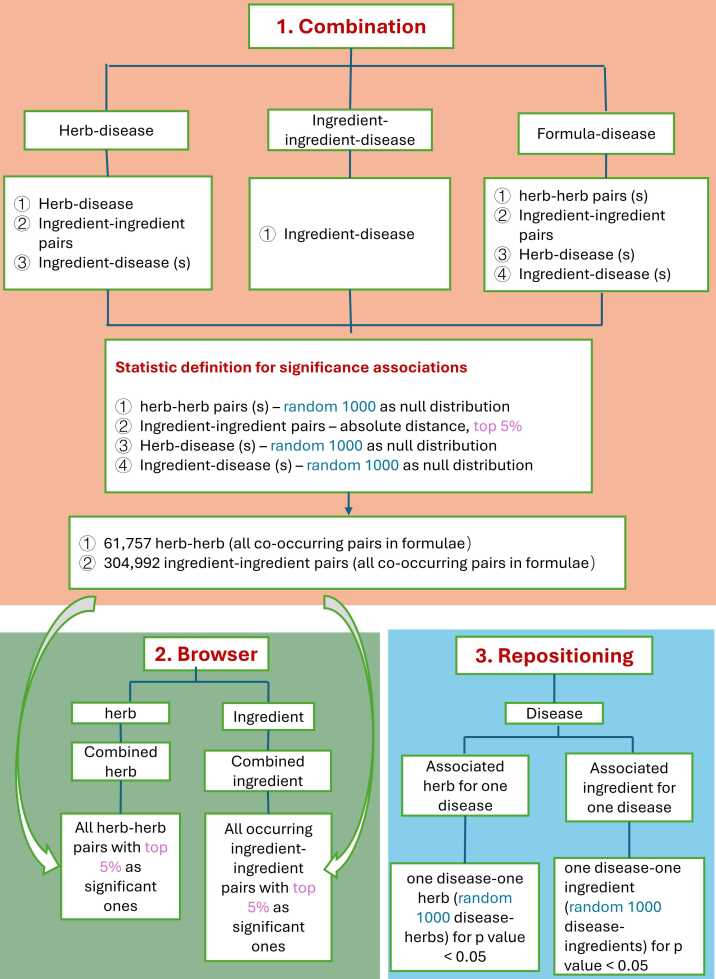
Varied statistical strategies tailored to different scenarios.

About the significance definition, we employed distinct statistical strategies tailored to different scenarios to ensure both computational feasibility and statistical robustness. As shown in Fig. 8, in combinational function, significant herb-herb, Ingredient-disease and herb-disease pairs within one formulae were selected by a one-tailed Fisher's Z-test for each herb pair using the formula: Z = (x - μ_null_)/σ_null_, where x is the observed proximity score, μ_null_ is the mean of the null distribution by 1000 random pairs, and σ_null_ is the standard deviation of the null distribution. Herb pairs with Z < 0.05 were considered statistically significant. It is essentially the proportion of the Z distribution cut off at the observed Z score. Secondly, for the Repositioning function, to recommend a significantly associated ingredient or herb for a disease, we also generated null distributions from 1000 randomly sampled pairs of herb-disease or ingredient-disease pairs per test. Thirdly, for browser functions, which recommend a combined ingredient for an ingredient and a combined herb for an herb, we adopted a different method from the ingredient pairs in herb and formula exploration. In this recommendation scenario, our null distribution (comprising 304,992 randomly sampled ingredient pairs) approximates a normal distribution (Fig. 3B). The 5 % significance threshold was determined empirically from this distribution. Here, we intentionally included similar ingredient pairs in our random sampling because they reflect real-world TCM practice, as outlined in the “JUN-CHEN-ZUO-SHI” theory, which holds that similar herbs are combined to enhance therapeutic effects. Excluding them would artificially bias the null distribution against clinically relevant combinations. However, with over 50,000 ingredients, exhaustive pairwise analysis (approximately 1.25 × 10^9 unique pairs) is computationally prohibitive. Instead, we constructed our null distribution here using all ingredient pairs that co-occur in existing formulae (n = 304,992). This strategy enables us to capture the proper distribution of target set proximities in our database, which reflects real TCM practice while maintaining statistical validity against an appropriate null model and reducing computational requirements from 2.5 × 10 ¹ ² to 10⁵ distance calculations. The extreme 5 % tail (lowest network distances) identifies pairs that are significantly closer than 95 % of random expectations. The network proximity approach identifies pairs with exceptionally close target interactions, a strong indicator of potential functional interplay (either synergistic or additive).

Several important limitations should be considered when interpreting our results. First, while the network proximity approach effectively identifies pairs with closely interacting targets—indicating potential functional interplay (synergistic or additive)—it cannot pharmacologically quantify combined effects relative to individual components, nor can it definitively distinguish synergy from additivity. Furthermore, the method primarily identifies potential positive interactions and does not specifically predict antagonistic interactions, which may involve opposing pathways not captured by simple distance minimization. Second, our approach inherently prioritizes well-studied herbs (e.g., Glycyrrhiza uralensis, Panax ginseng) with abundant target data, potentially overlooking under-investigated botanicals. This reflects systemic biases in the existing literature and compiled databases. Consequently, network predictions may favor frequently studied herbs, possibly missing synergistic potential in less-characterized resources. Third, although HerbComb introduces an ADMET-aware screening strategy—identifying, for instance, 9 of 32 ischemic stroke-targeted compounds with favorable bioavailability and blood-brain barrier permeability—this represents only an initial pharmacokinetic filter. True organism-level effects require consideration of additional biological complexities, including gut microbiome interactions (biotransformation, metabolic activation), tissue-specific distribution, and off-target signaling effects. Notwithstanding these limitations, HerbComb provides valuable insights, as demonstrated in our case study of the Tongxinluo formula for stroke, where we identified Oleanolic acid and Ferulic acid as previously unreported synergistic ingredients. Looking forward, we envision several directions for enhancement: incorporating transcriptomic data to determine regulatory directions for synergy or antagonism, integrating microbiome interaction predictions, developing dynamic PK/PD models, and incorporating clinical correlation data. Importantly, all computational predictions generated by HerbComb require experimental confirmation through in vitro, organoid, or in vivo studies, and traditional knowledge remains essential for properly contextualizing the results. Also, functional similarity or pathway coherence could be incorporated into our database to strengthen confidence in predicted synergy.

In summary, we developed HerbComb, a novel web-based platform for characterizing synergistic interactions among herbal medicines. This platform provides new insights into synergistic mechanisms and facilitates the discovery of effective drug combinations for disease treatment.

## Data and software availability

The datasets supporting this study are available upon request. For the review process, the data can be accessed via a restricted Zenodo private link: https://tinyurl.com/mwya586m. It includes ADMET properties of ingredients, as well as information on formulas, traditional Chinese medicines, ingredients, and related diseases.

## Author agreement

We the undersigned declare that this manuscript entitled “HerbComb: an integrated database for the discovery of novel combinational therapies from herbal medicines” is original, has not been published before and is not currently being considered for publication elsewhere. We confirm that the manuscript has been read and approved by all named authors and that there are not other persons who satisfied the criteria for authorship but are not listed. We further confirm that the order of authors listed in the manuscript has been approved by all of us. We understand that the corresponding author is the sole contact for the Editorial process. He is responsible for communicating with the other authors about progress, submissions of revisions and final approval of proofs.

## CRediT authorship contribution statement

**Boon Seng Kho:** Data curation. **Xiaochuang Xu:** Visualization, Data curation. **Jing Tang:** Writing – review & editing, Supervision, Project administration, Funding acquisition. **Hao Liu:** Formal analysis. **Chengyuan Yue:** Data curation. **Yun Tang:** Supervision. **Xiang Luo:** Visualization, Software, Data curation. **Tao Yang:** Writing – review & editing, Supervision. **Jiaqi Yao:** Methodology, Formal analysis. **Lin Cao:** Software. **wang yinyin:** Writing – review & editing, Writing – original draft, Methodology, Funding acquisition, Formal analysis, Conceptualization. **Ninghua Tan:** Supervision. **Rui Liu:** Writing – original draft, Methodology, Formal analysis, Data curation. **Fangcheng Yu:** Visualization. **Yinnan Zhang:** Visualization. **Shixing Lai:** Visualization.

## Code availability

The core computational pipelines for the network proximity analysis and statistical validation, implemented in Python/NetworkX, have been made publicly available on GitHub at: https://github.com/19900321/HerbComb.

## Declaration of Competing Interest

The author(s) declared no potential conﬂicts of interest with respect to the research, authorship, and/or publication of this article.
