# Supplementary material for: HerbComb: An integrated database for the discovery of novel combinational therapies from herbal medicines
Source: Comput Struct Biotechnol J. 2025 Nov 6;27:4915–35. doi: 10.1016/j.csbj.2025.10.065 (PMC12657374; doi:10.1016/j.csbj.2025.10.065)
Supplement: Supplementary file 1 — Supplementary material [file mmc1.docx]

# HerbComb Guidance

## Tutorial for browsing prescriptions in HerbComb

### Step 1: Users can browse the basic information of Herb prescriptions by clicking the prescription section in the home page of HerbComb.


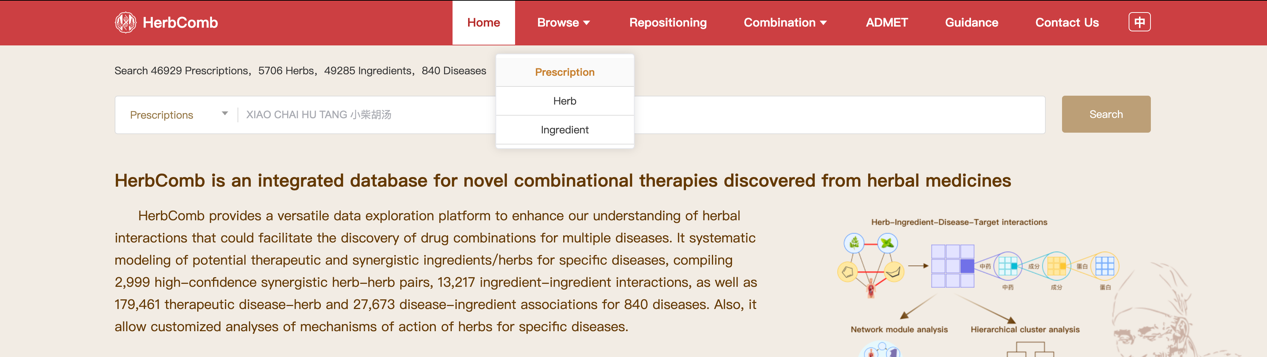


**Basic information of Herb prescriptions**


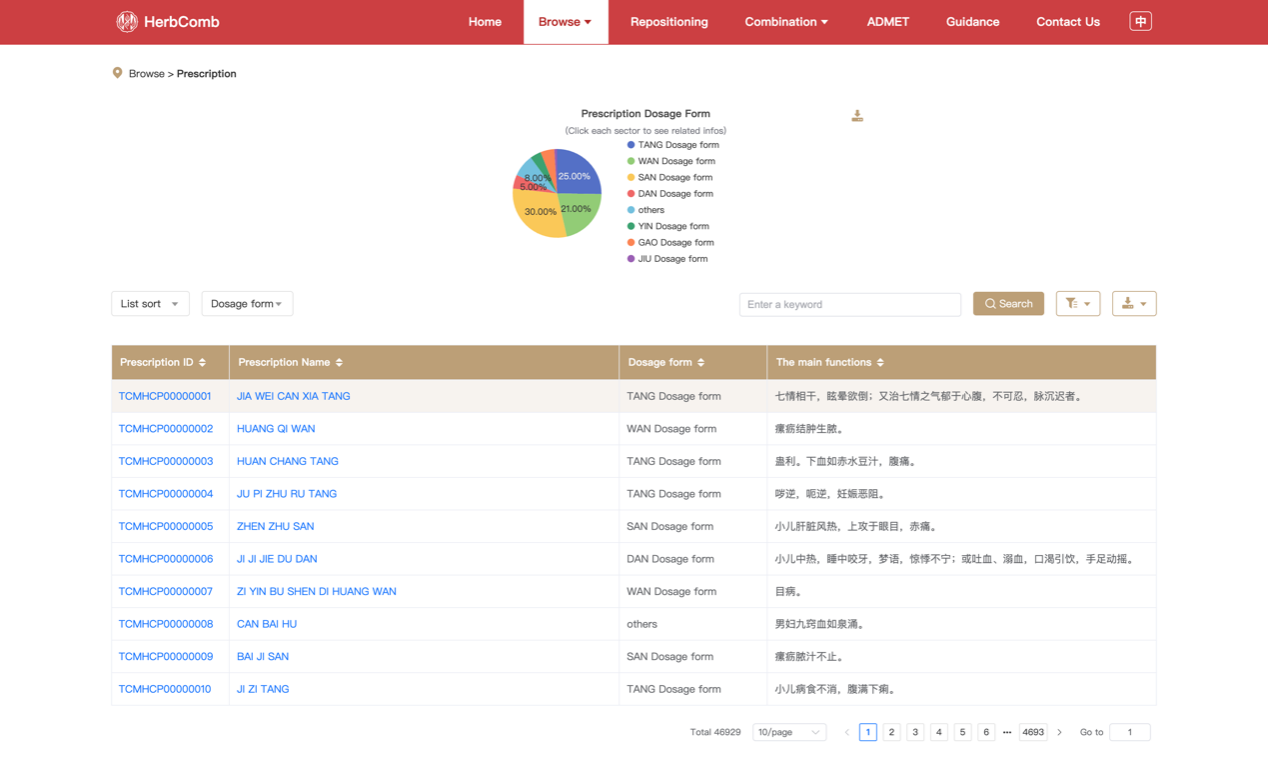


**Link to the detailed information of a**

**Herb prescription.**

**Users can select Herb prescriptions**

**according to their types.**

**Users can search a prescription by its name in Pinyin，as well as export and save the prescriptions list.**

### Step 2: Users can browse the detailed information of a Herb prescription by clicking the prescription name.


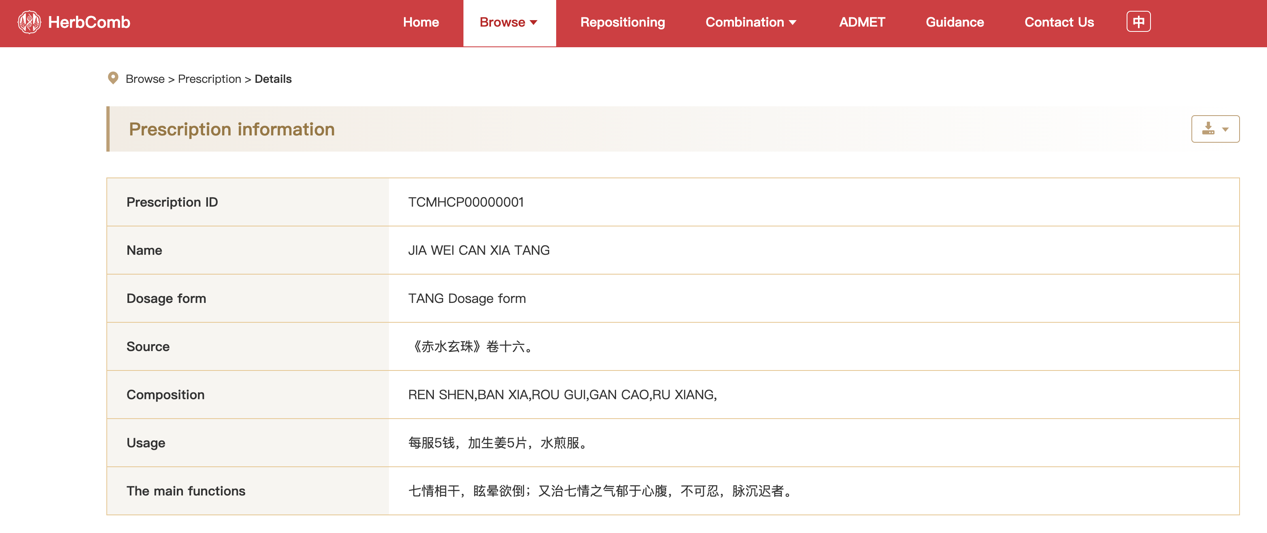


**Basic information and Herb features of a Herb prescription.**


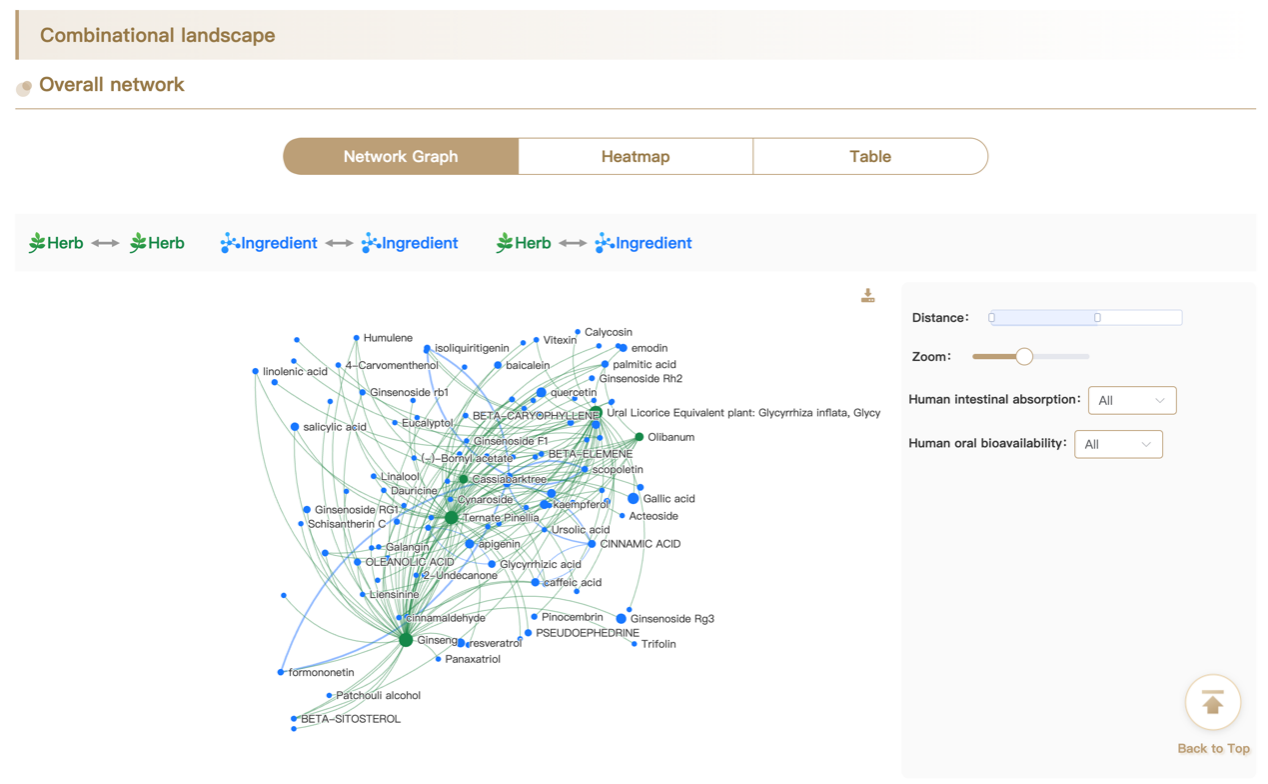


**Click on the relationship legend to combine and view the related network distance relationships.**

**Switch tabs to view the network diagram, heatmap, and information list related to the Herb expression.**


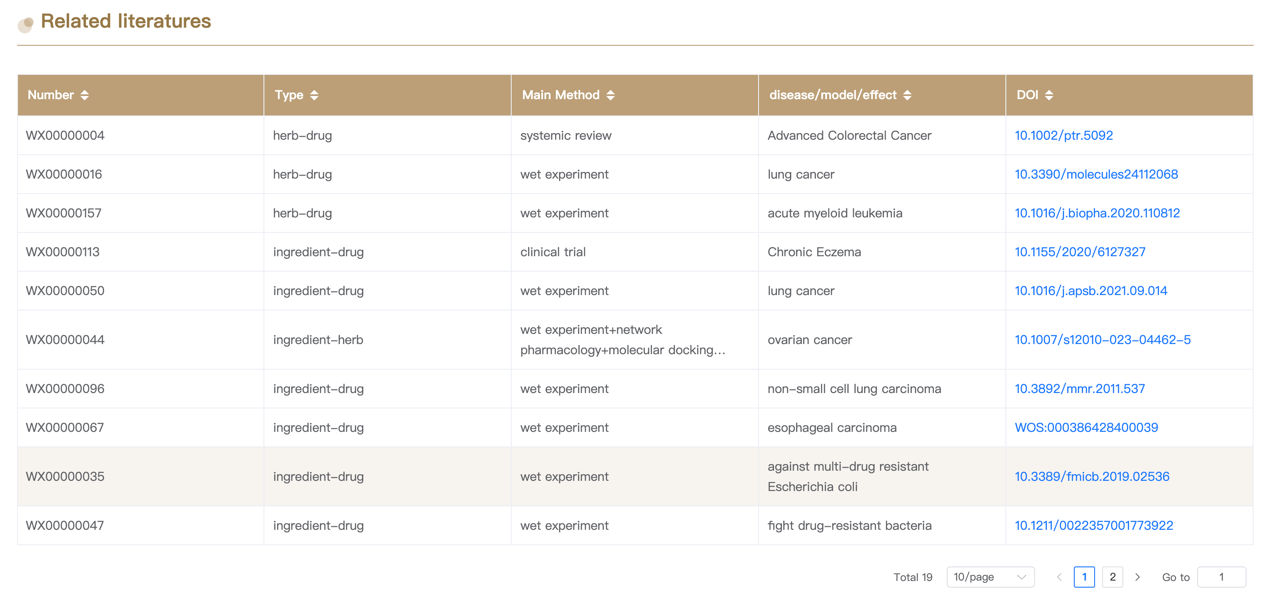


**Click to view the original details of Chinese and Western literature related to the Herb prescription.**

**1、Link to the detailed information of a herb containing this Herb prescription.**

**2、Link to the detailed information of ingredients containing this Herb prescription.**

**3、Link to the detailed information of targets containing this Herb prescription.**


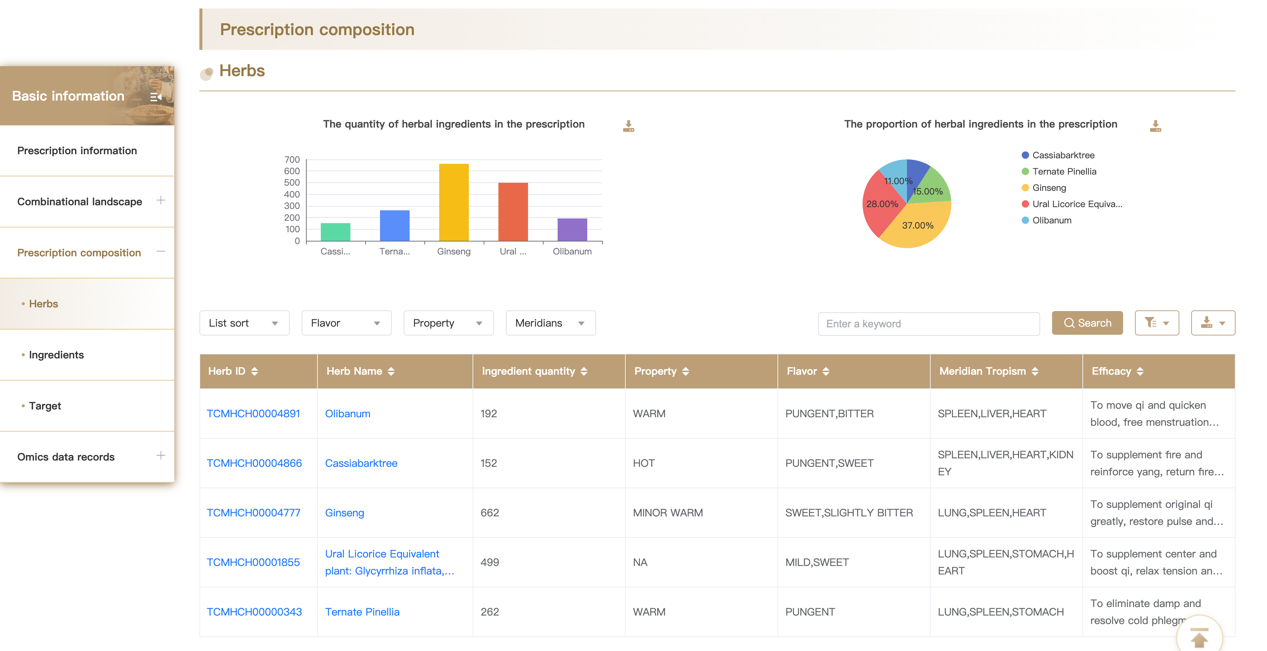


**Users can search a herb by its name，as well as  export and save the herbs list.**

**Users can select Herb prescriptions according to their types.**

**Link to the detailed information of a herb.**


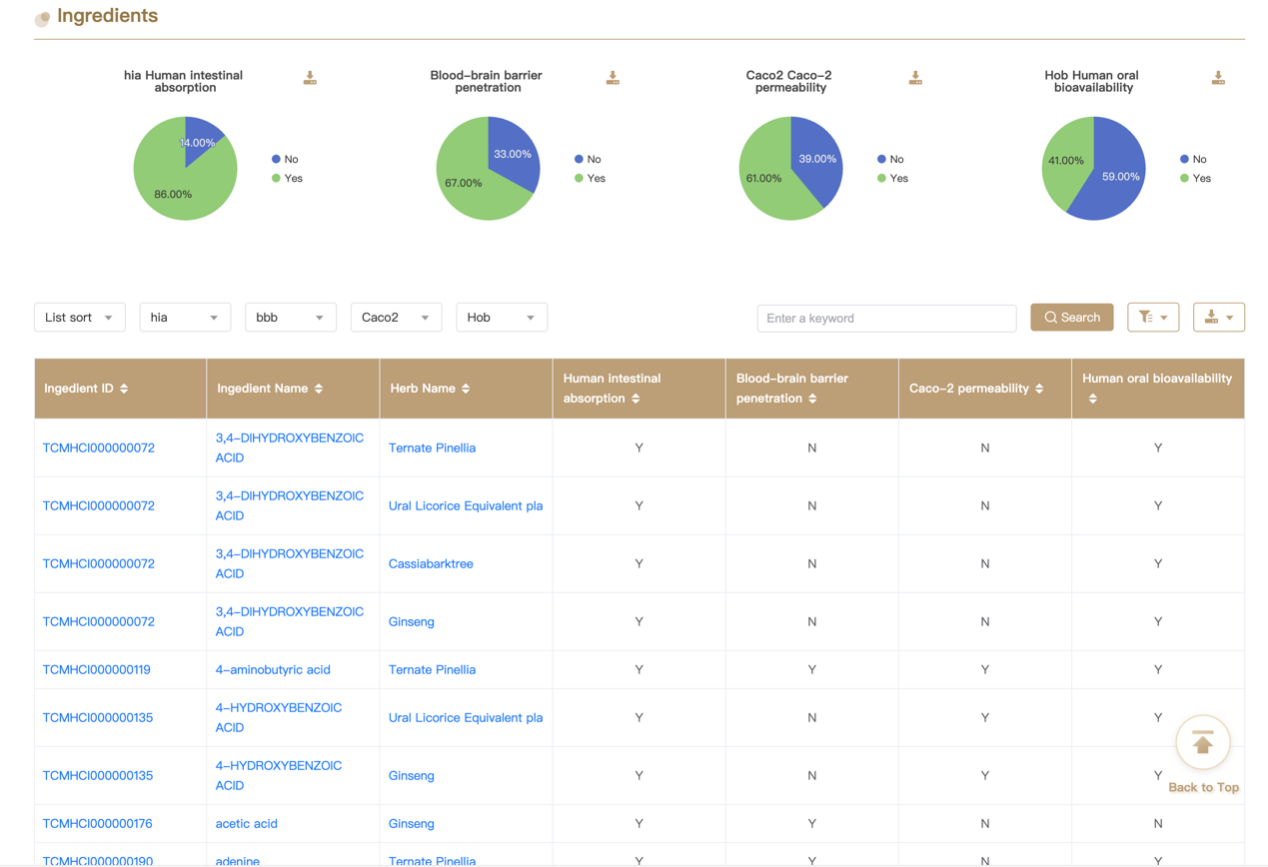


**Link to the detailed information of a herb.**

**Link to the detailed information of a ingredient.**

**Users can quickly select the components belonging to that type by clicking on the pie chart.**

**
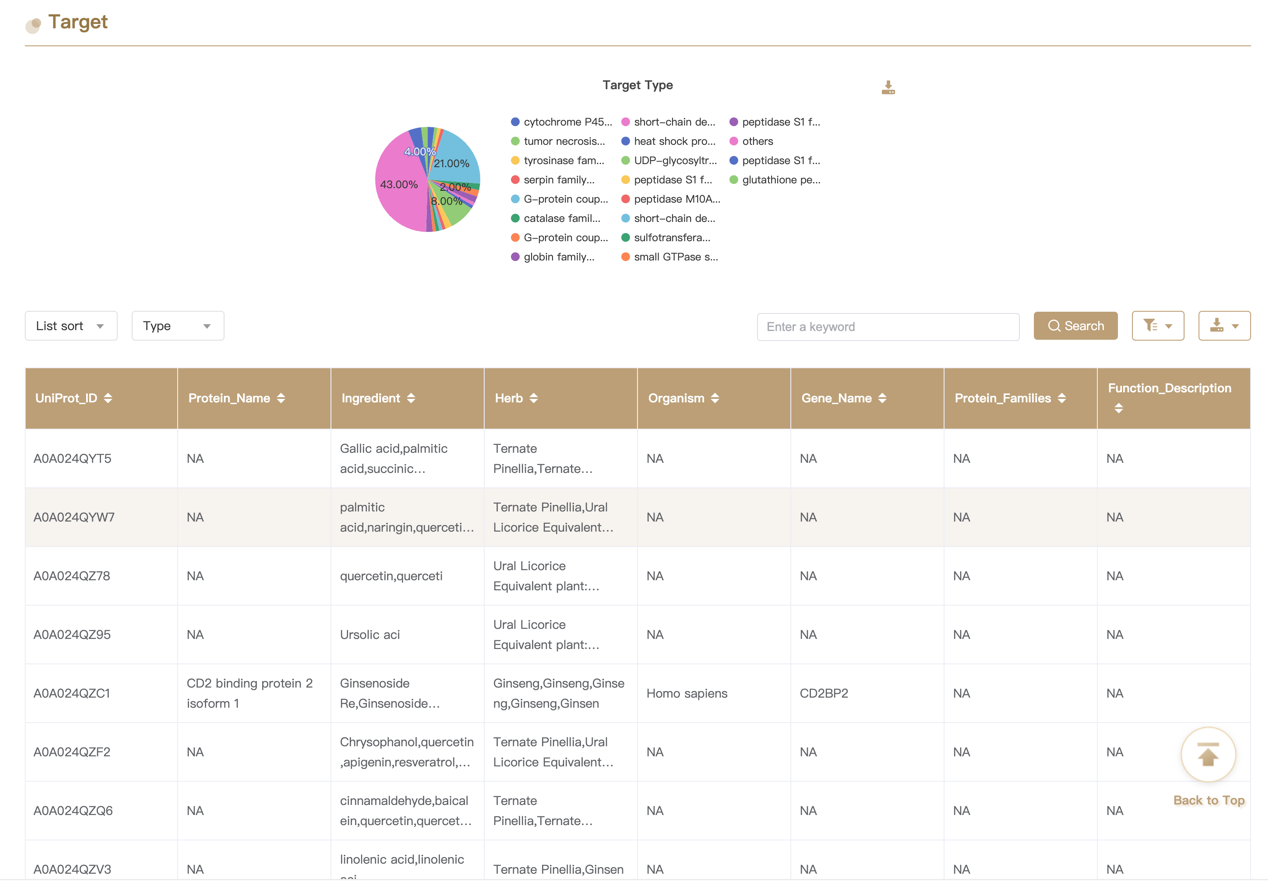
**

**Click to view the information of herbs related to a Target.**

**Click to view the information of ingredients related to a Target**.


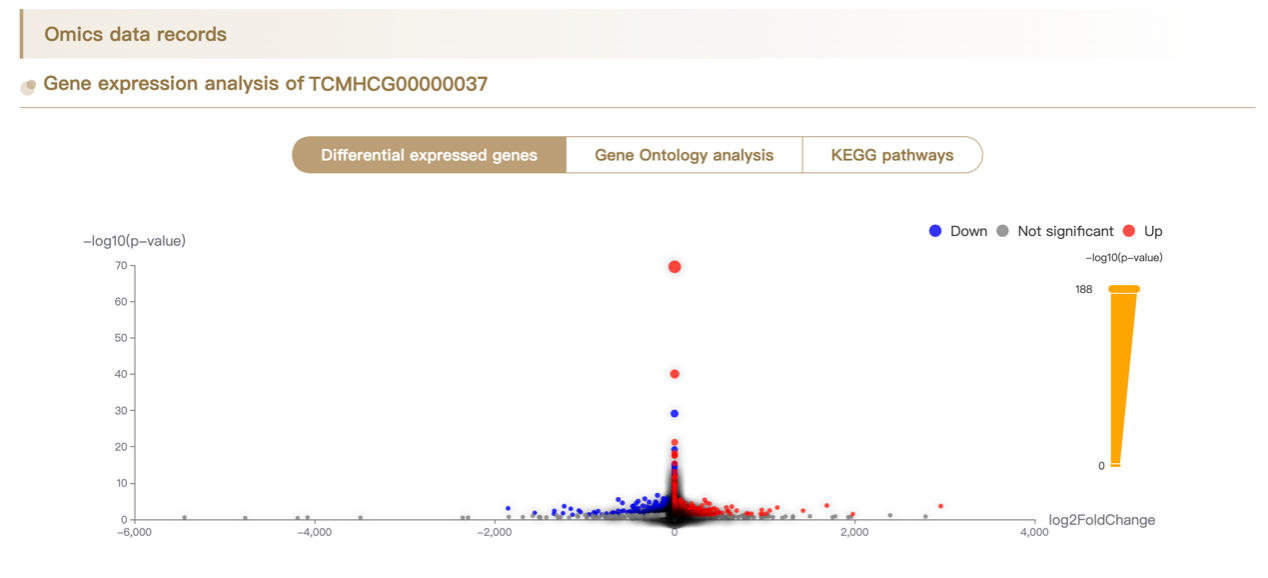


**Switch tabs to view a Gene expression analysis Information of Differential expressed genes、Gene Ontology analysis、KEGG pathways.**


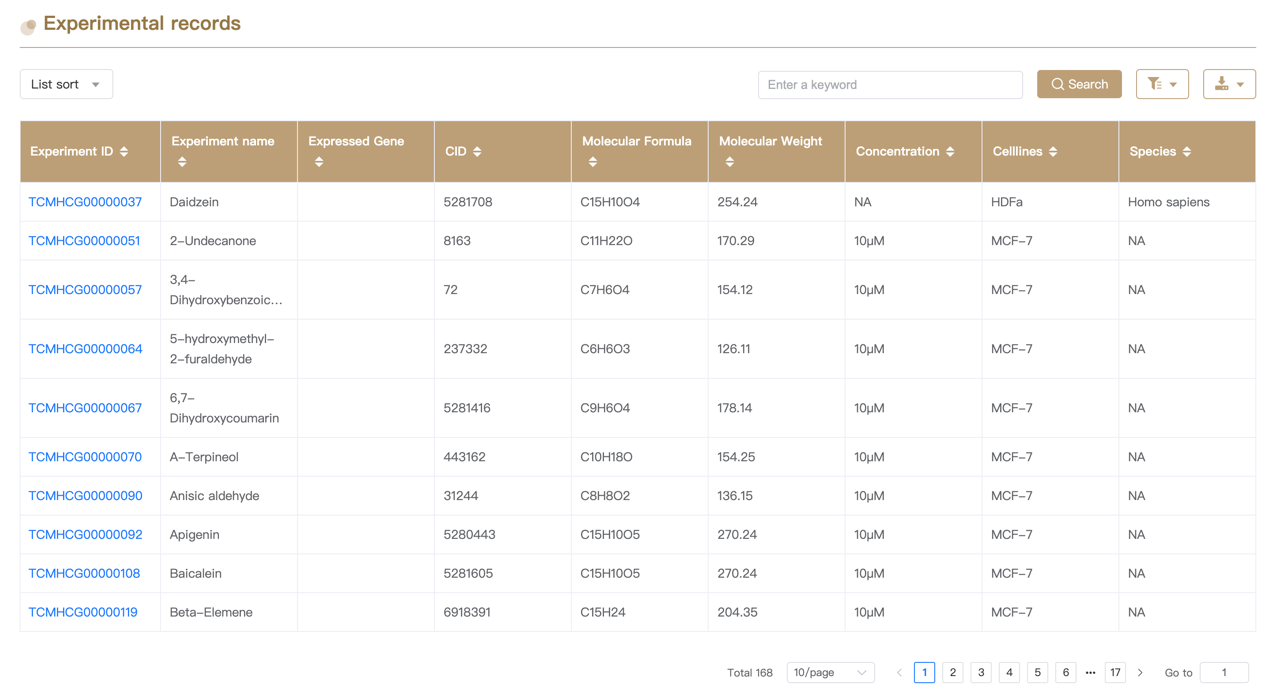


**Click to view charts of other gene expression analysis records.**

## Tutorial for browsing herbs in HerbComb

### Step 1: Users can browse the basic information of herbs by clicking the herb section in the home page of HerbComb.


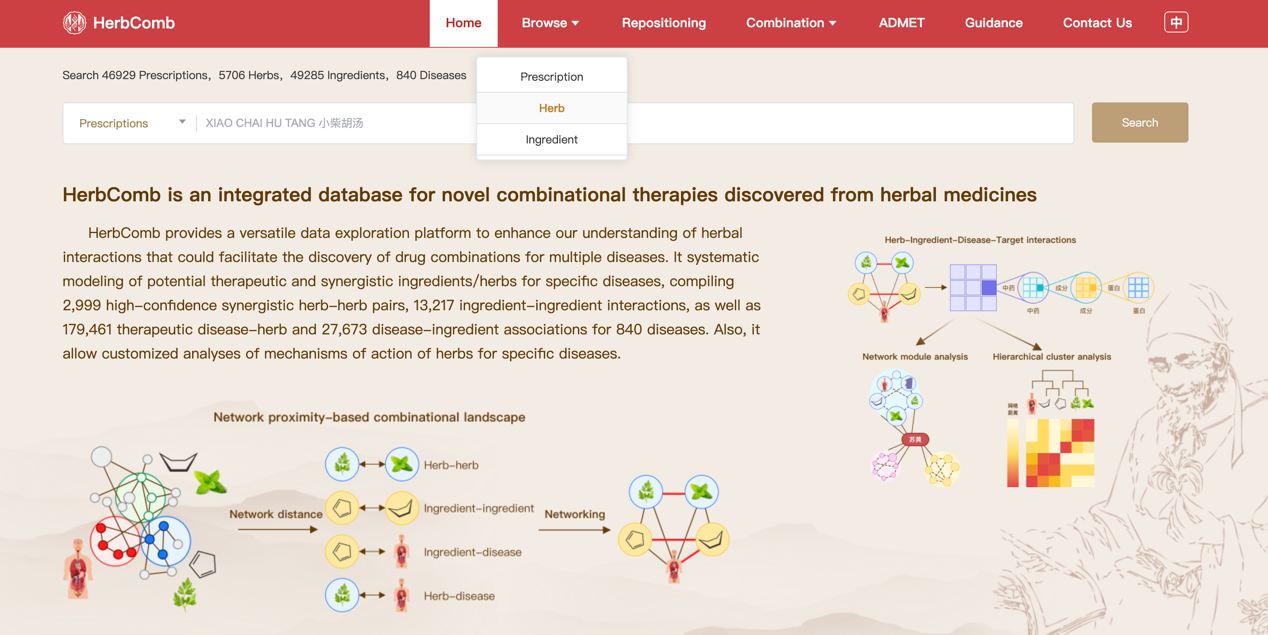


**Basic information of herbs**


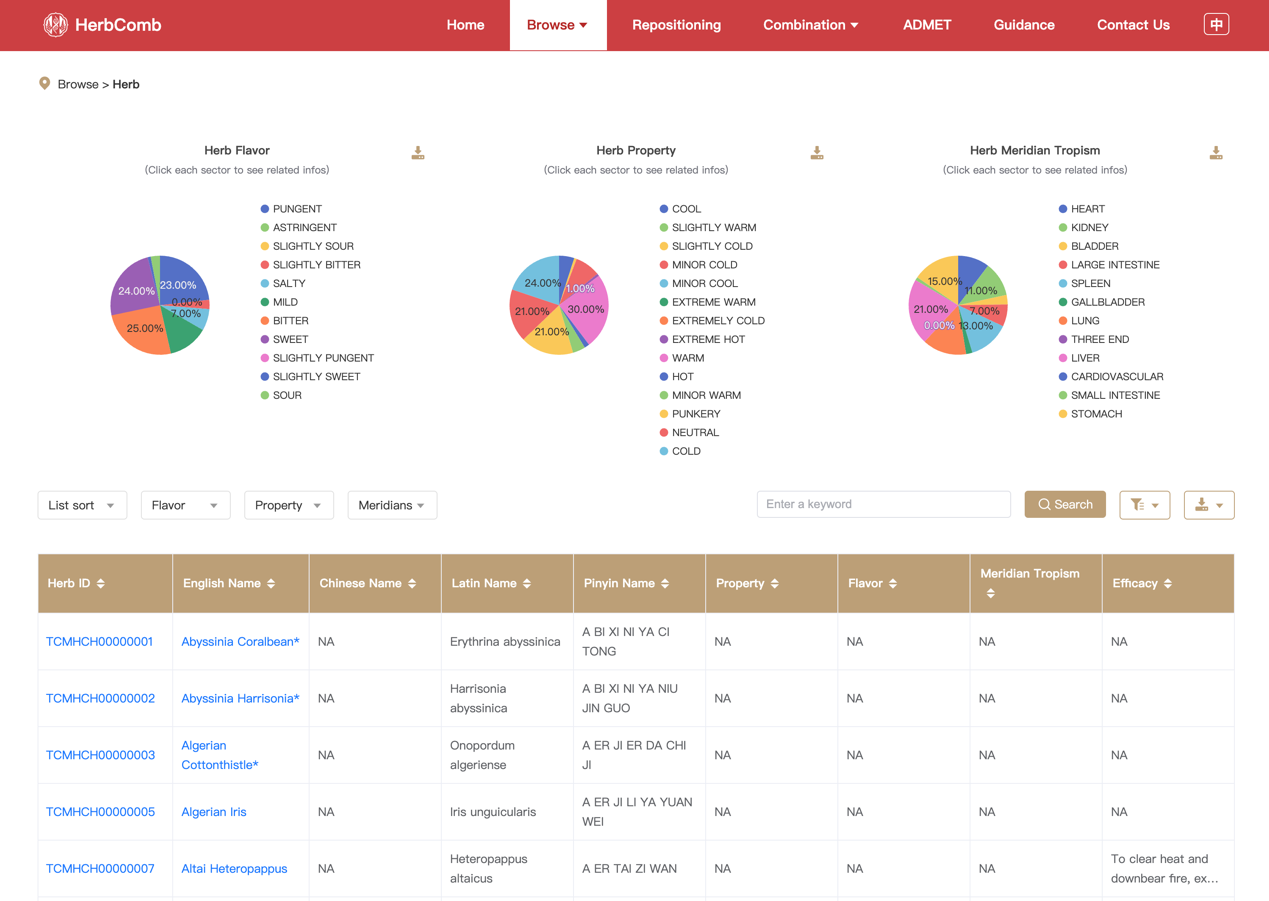


**Link to the detailed information of a herb.**

**Users can search a herb by its name，as well as  export and save the herbs list.**

**Users can select herbs according to their types.**

### Step 2: Users can browse the detailed information of a herb by clicking the herb name.


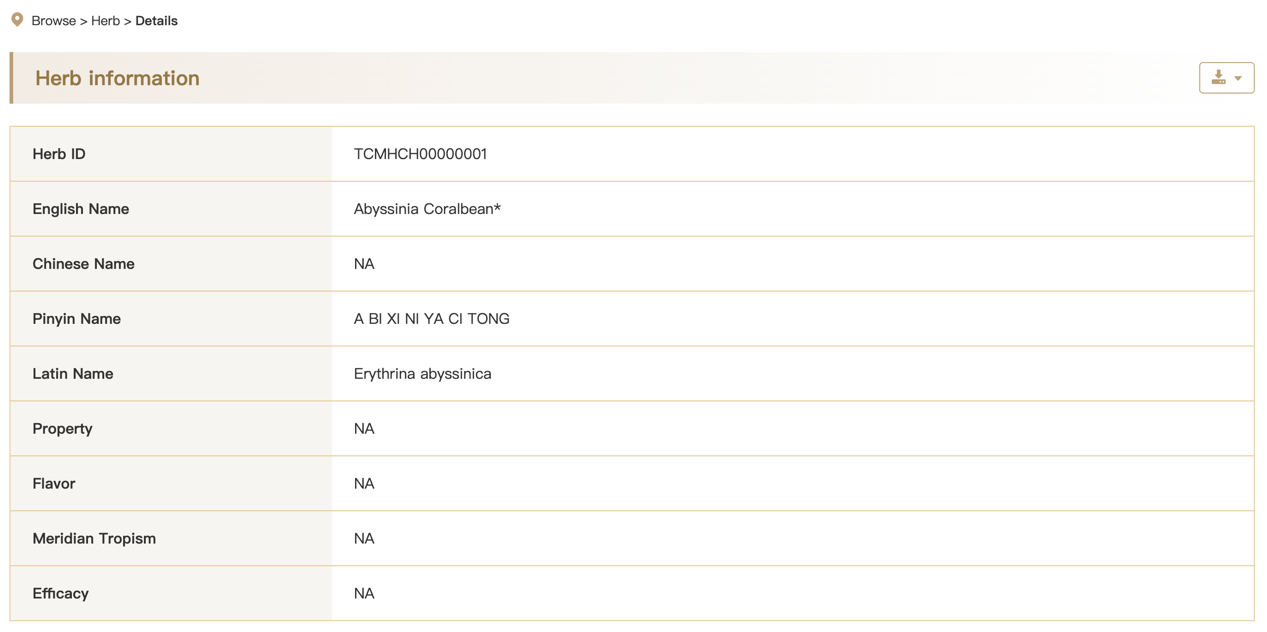


**Basic information and Herb features of a Herb prescription.**


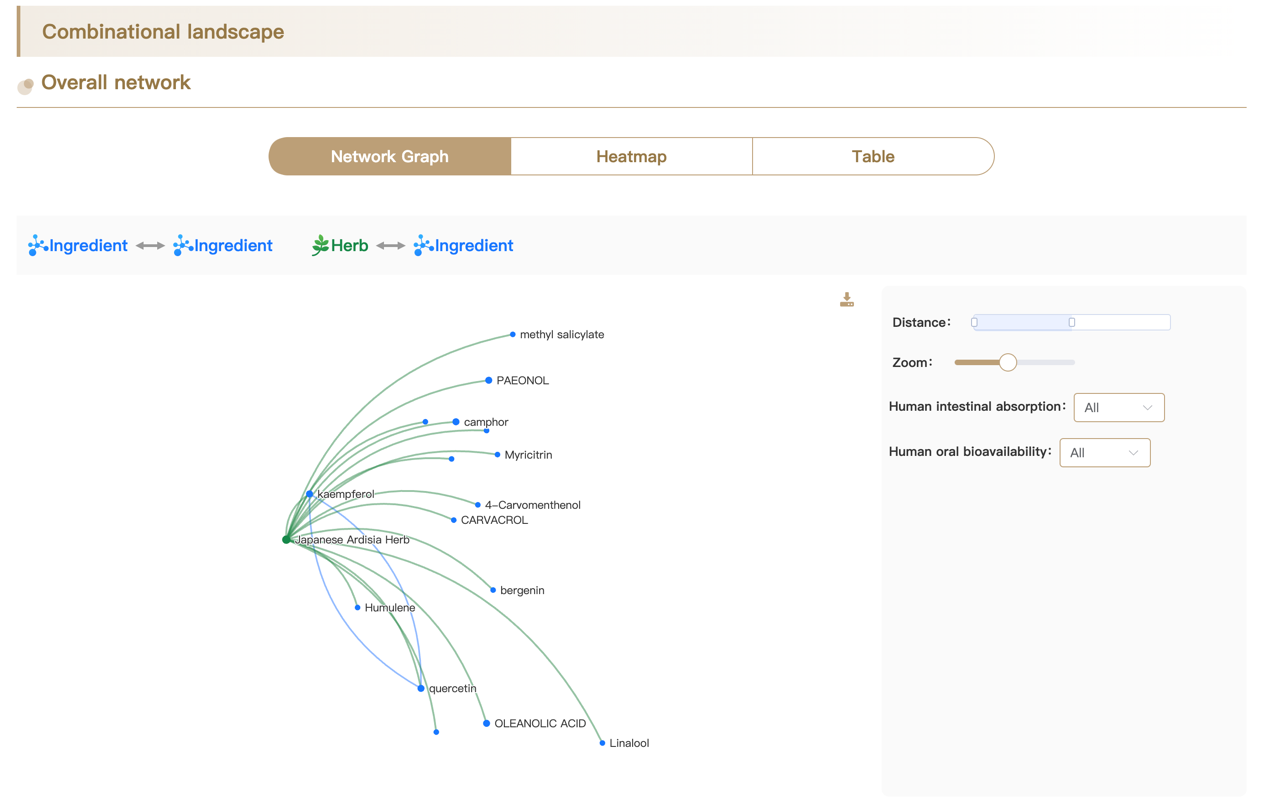


**Click to jump to the Combinatorial Analysis 1: Herb-Herb page to view the details of the network distance relationship chart between the two herbs.**

**Click on the relationship legend to combine and view the related network distance relationships.**

**Switch tabs to view the network diagram, heatmap, and information list related to the Herb herb.**


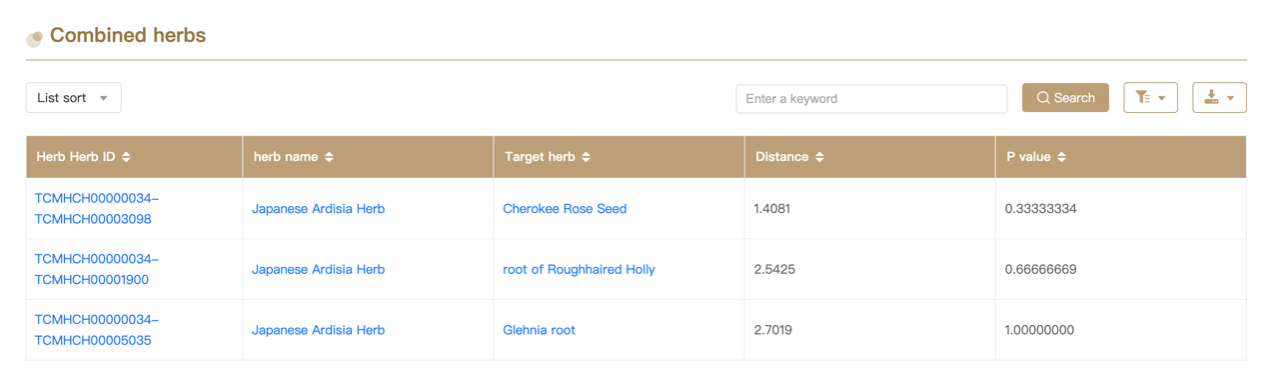


**Click to view the relevant herb details.**


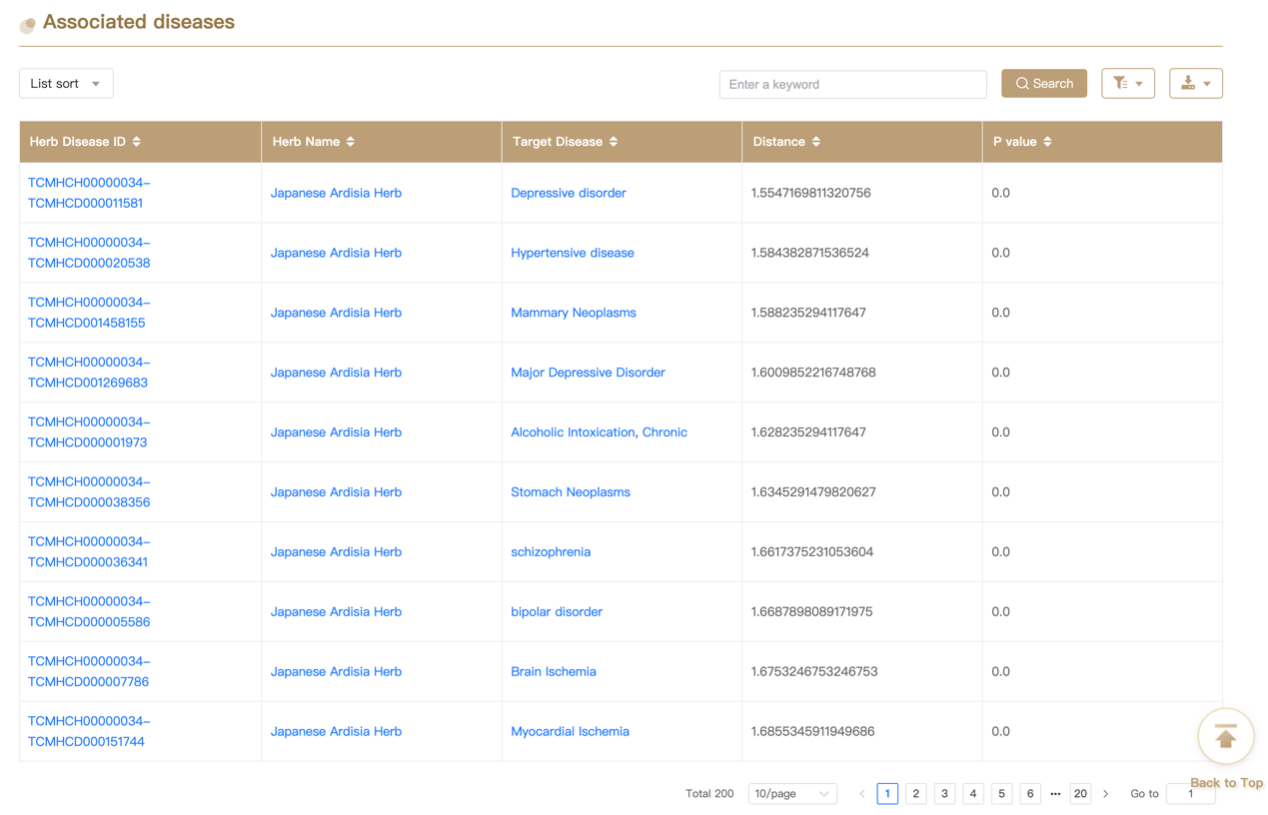


**Click to view the relevant disease details.**

**Click to jump to the Combinatorial Analysis4 : A Disease- herbs page to view the details of the network distance relationship chart between a disease and a herb.**


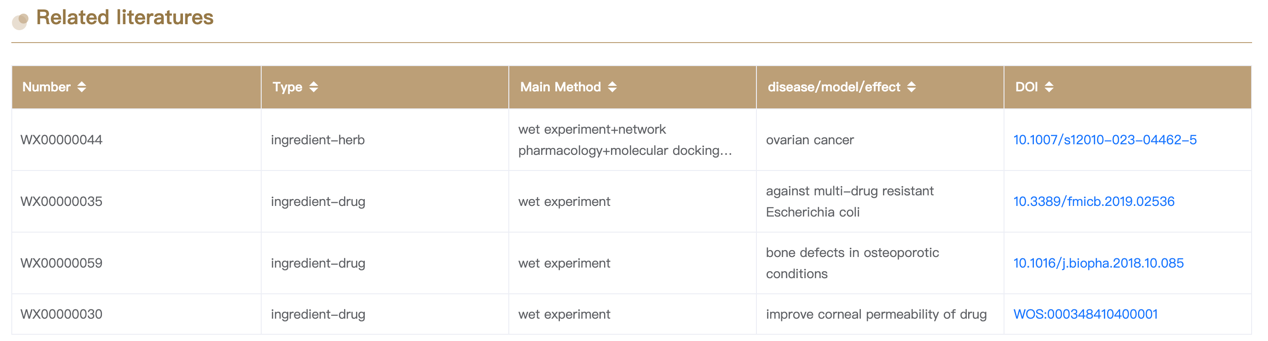


**Click to view the original text details of the Chinese and Western literature related to the herb.**


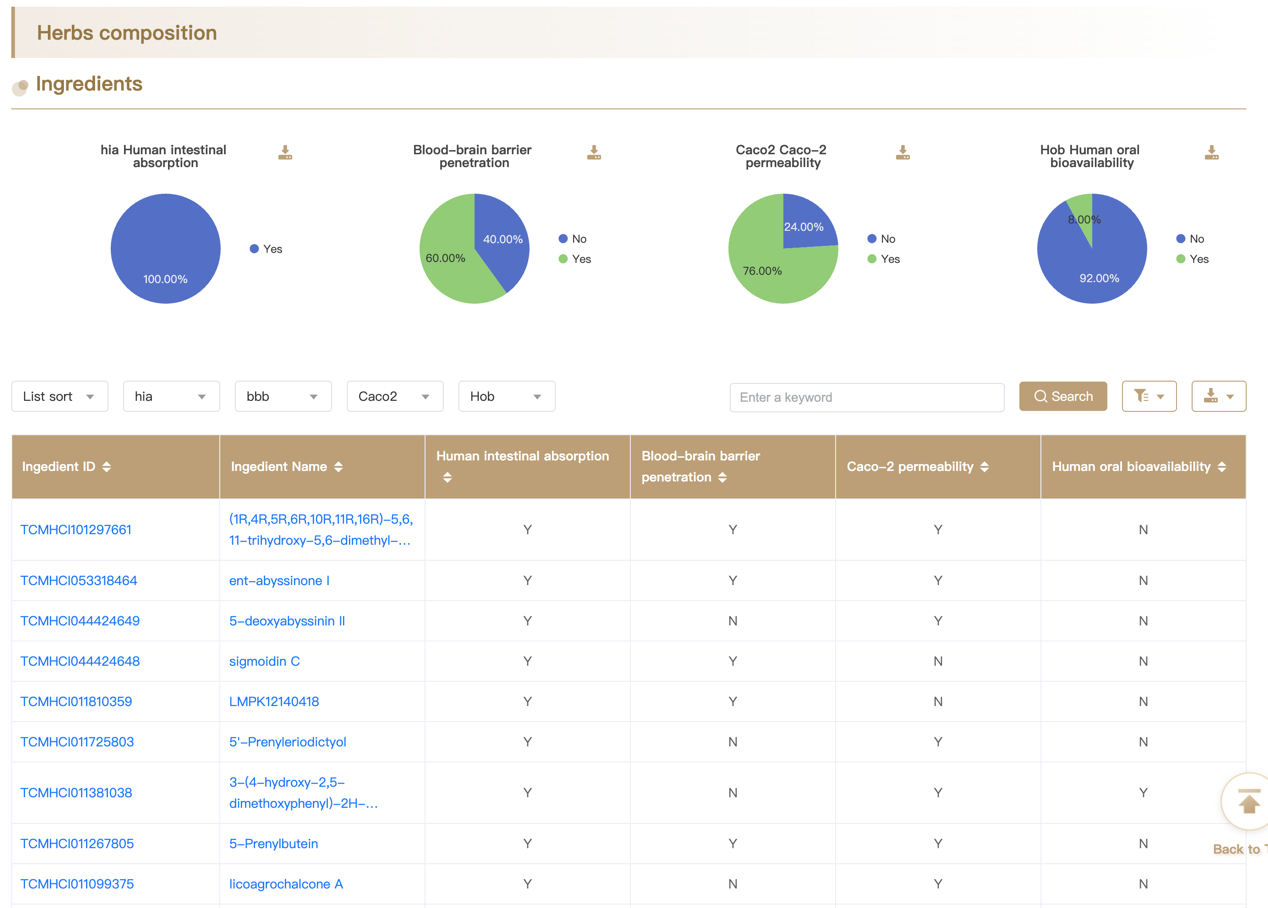


**Link to the detailed information of a ingredient.**

**Users can select Herb herbs according to their types.**


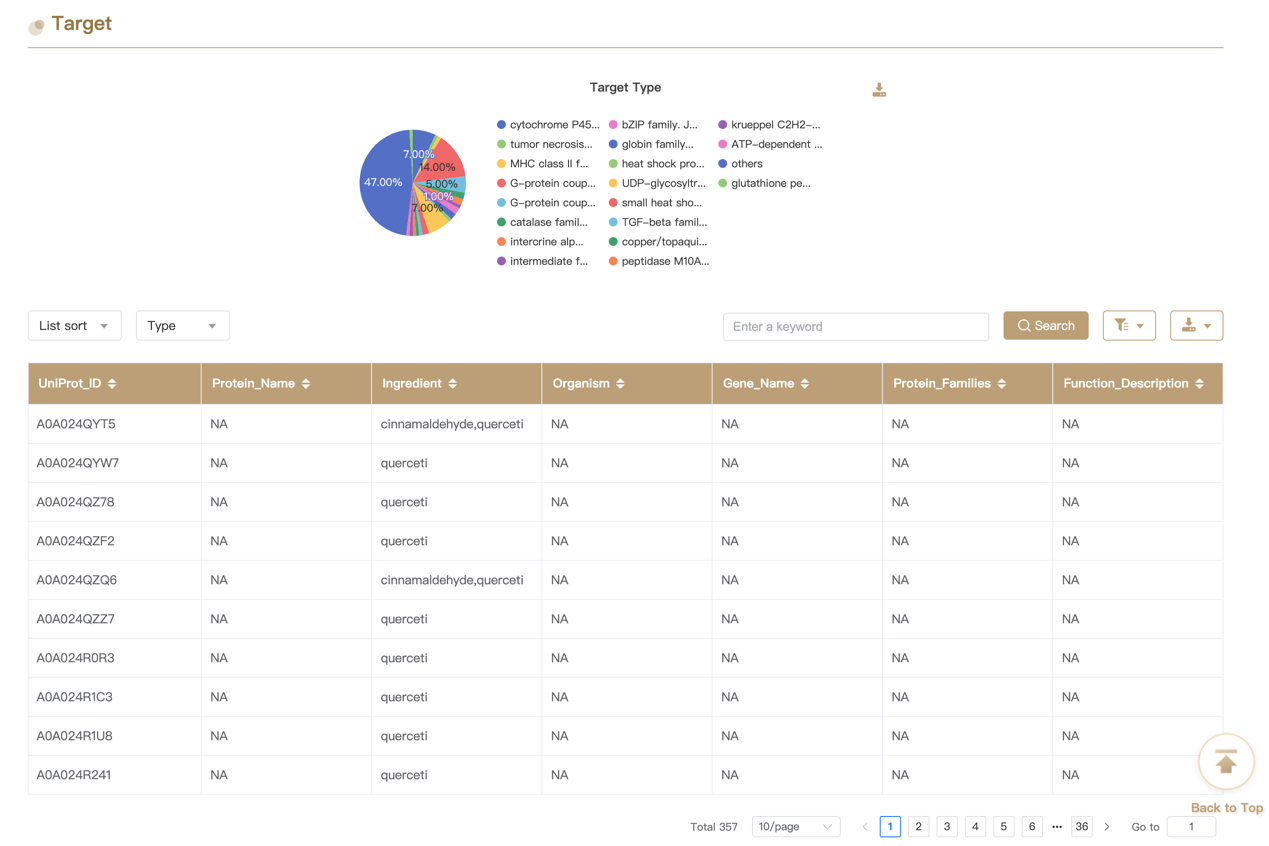


**Click to view the information of a ingredient related to a Target.**


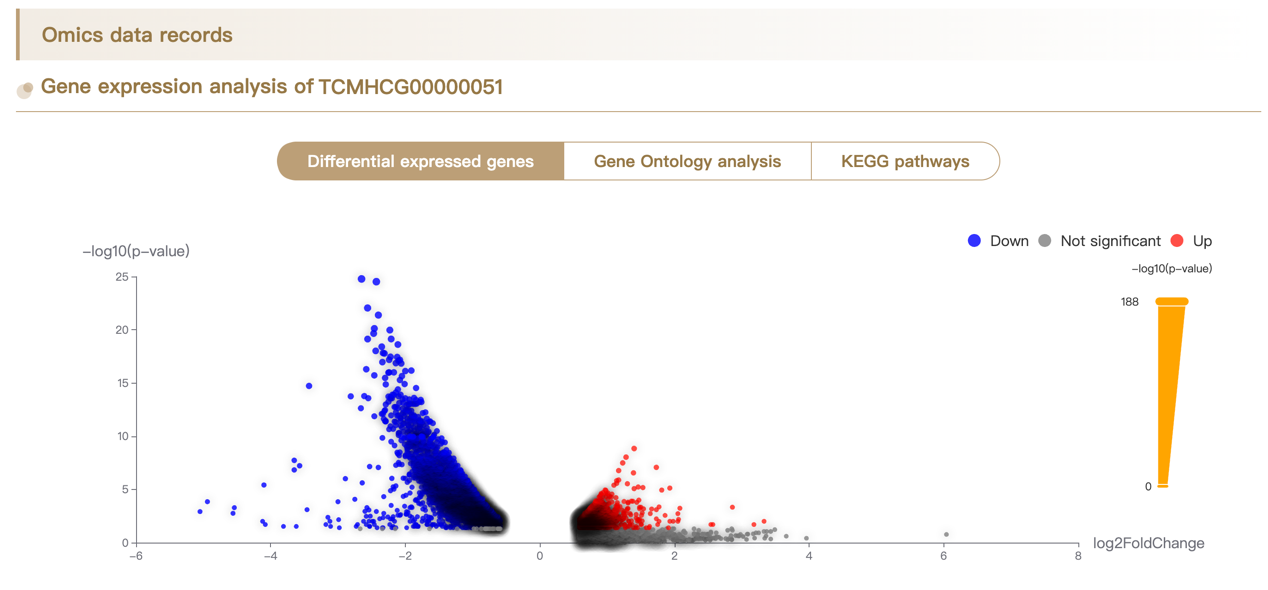


**Switch tabs to view a Gene expression analysis Information of Differential expressed genes、Gene Ontology analysis、KEGG pathways.**


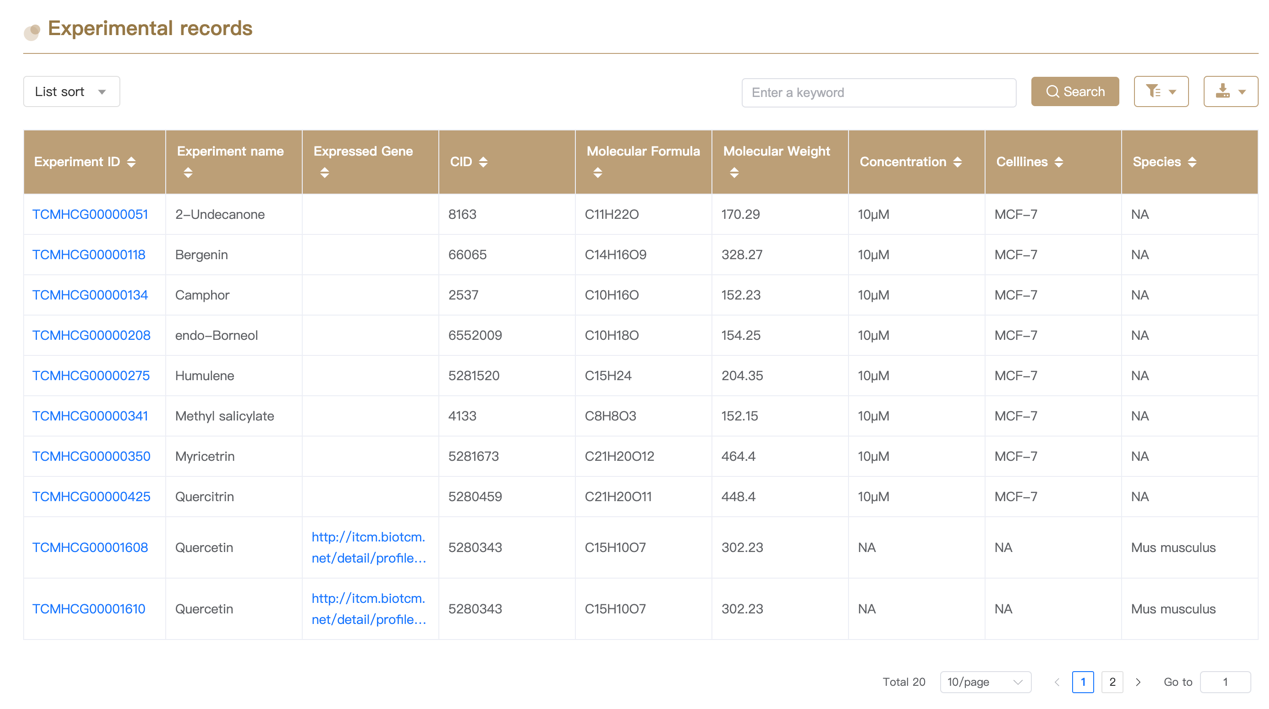


**Click to view charts of other gene expression analysis records.**


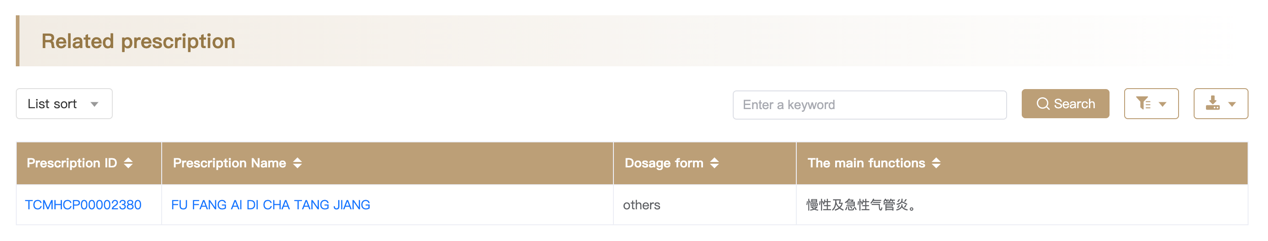


**Click to view the details of the Herb prescription related to the herb.**

## Tutorial for browsing ingredients in HerbComb

### Step 1: Users can browse the basic information of ingredients by clicking the ingredients section in the home page of HerbComb.


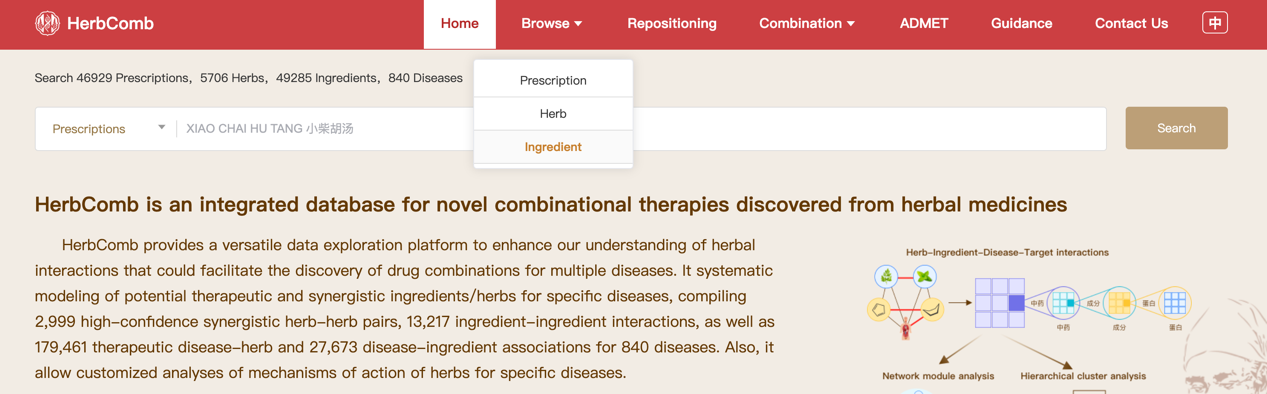


**Basic information of ingredients**


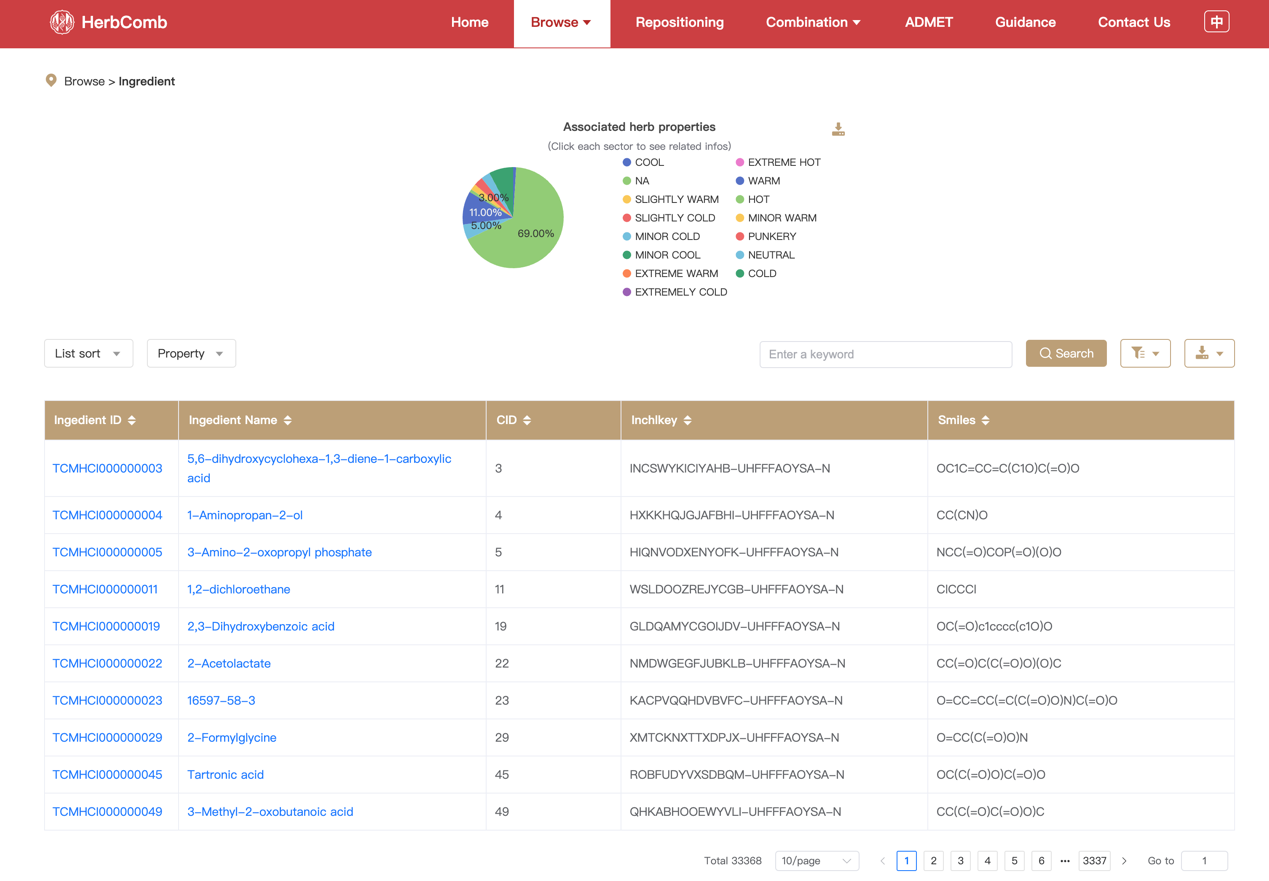


**Link to the detailed information of a ingredient.**

**Users can select ingredients according to their types.**

**Users can search a ingredient by its name，as well as  export and save the ingredients list.**

### Step 2: Users can browse the detailed information of a ingredient by clicking the ingredient name.


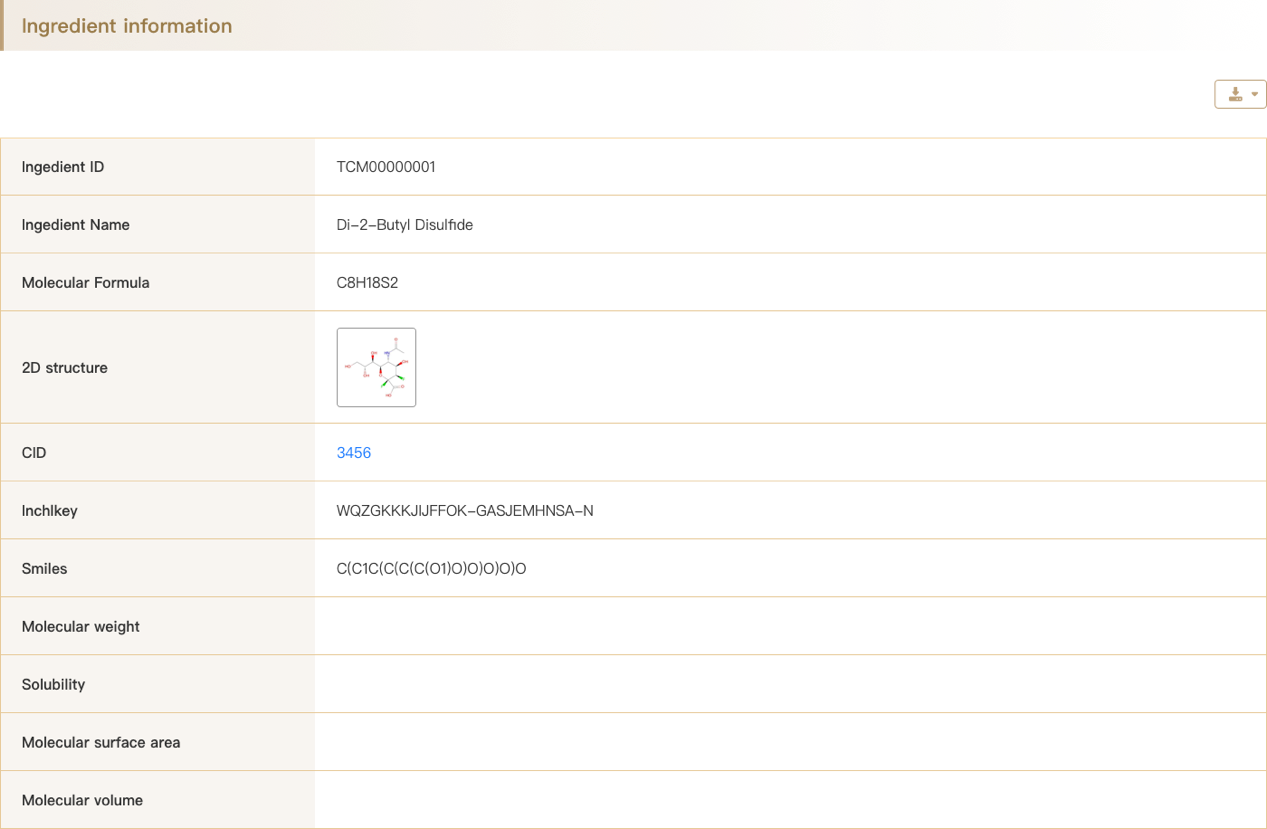


**Basic Information and Characteristics of ingredient.**

**
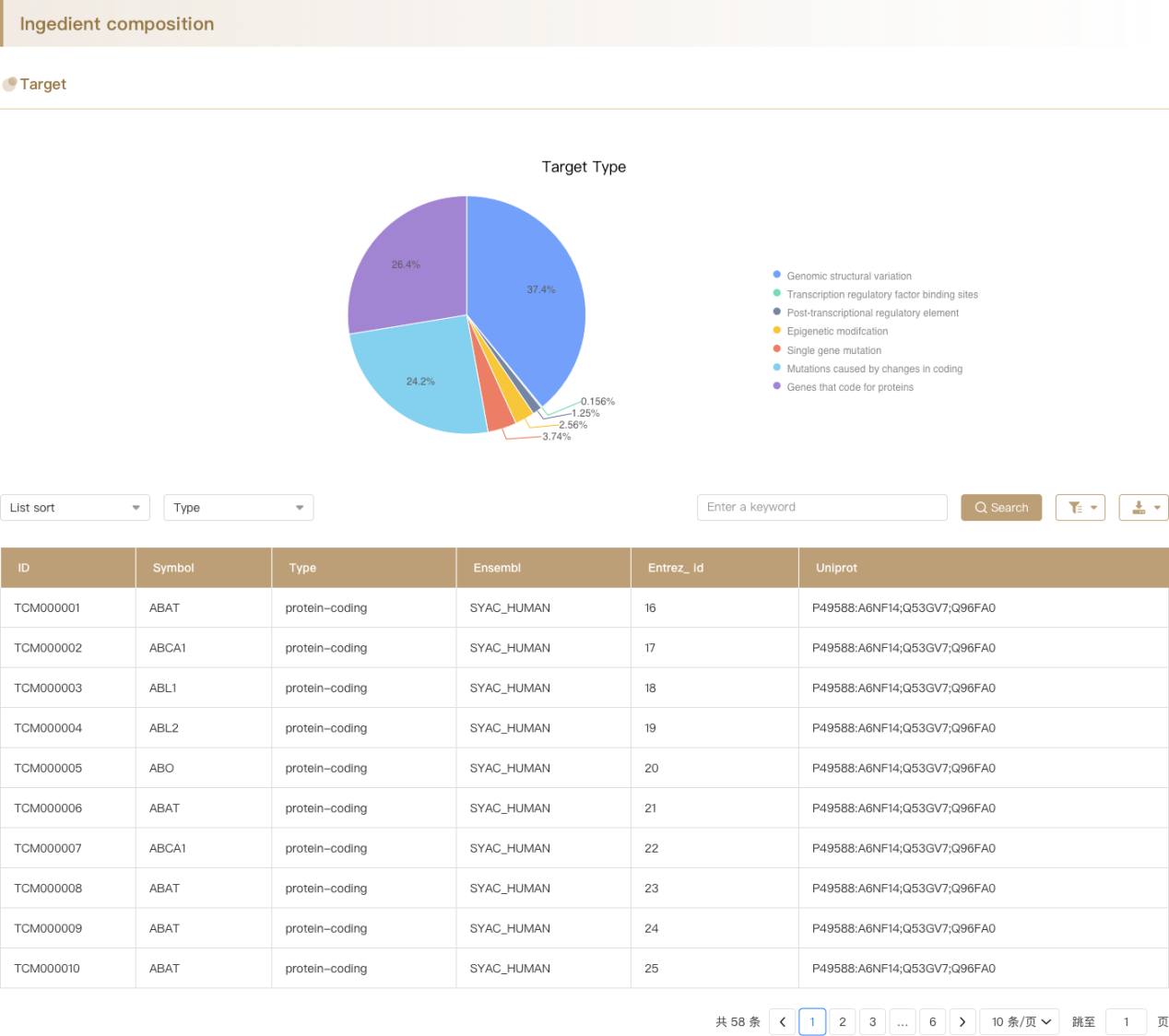
**

**Users can select ingredients according to their types.**


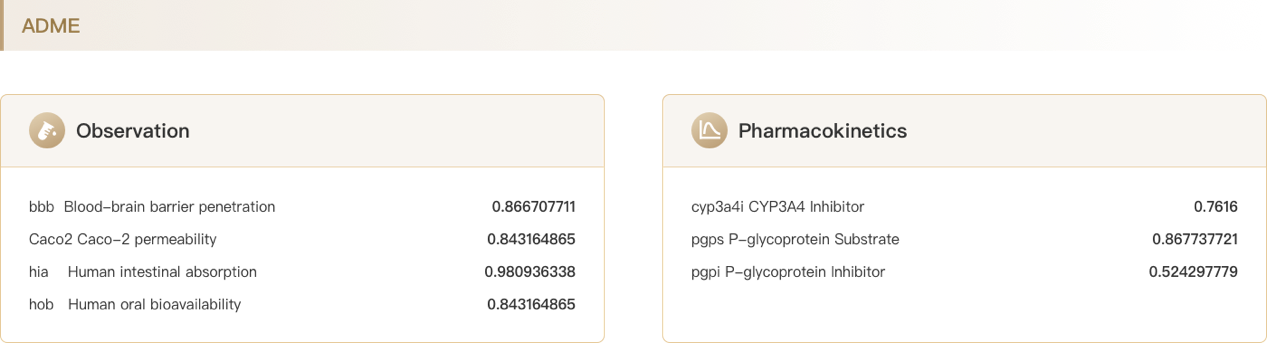


**View the details of the Observation indicators of the ingredient.**

**View the details of the Observation indicators of the ingredient.**


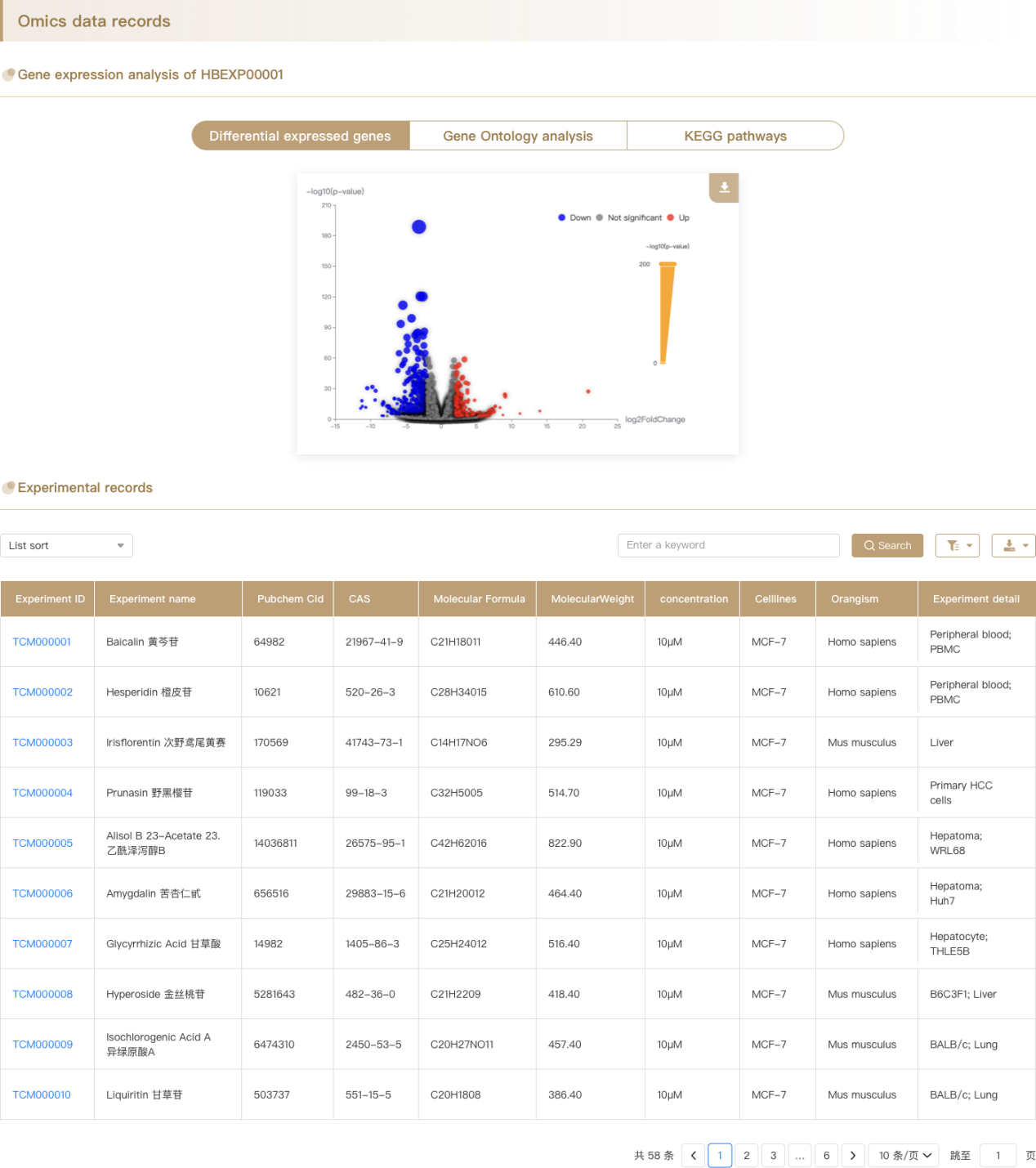


**Click to view charts of other gene expression analysis records.**

**Switch tabs to view a Gene expression analysis Information of Differential expressed genes. Gene Ontology analysis、KEGG pathways.**


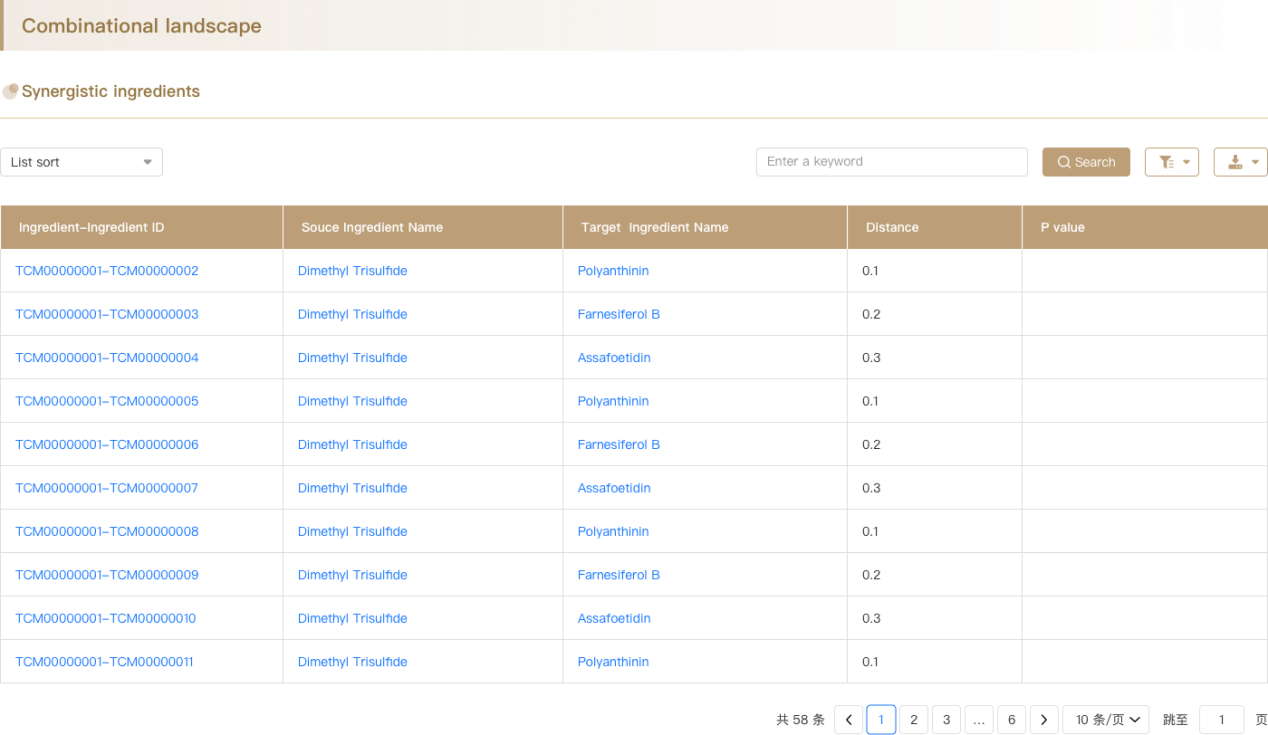


**Click to view the relevant ingredient details.**

**Click to jump to the Combinatorial Analysis 2: ingredient- ingredient page to view the details of the network distance relationship chart between the two ingredients.**


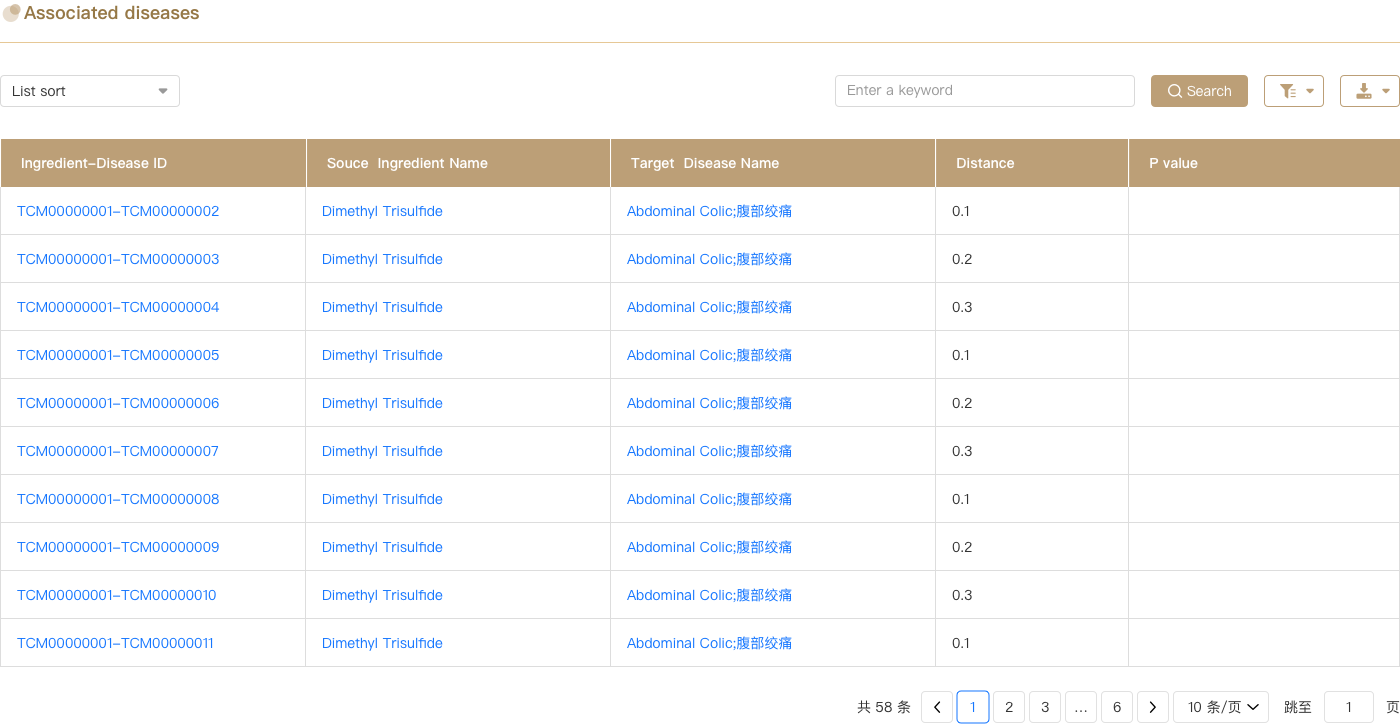


**Click to view the relevant ingredient details.**

**Click to jump to the Combinatorial Analysis 5: A Disease-ingredients page to view the details of the network distance relationship chart between a Disease and the ingredient.**


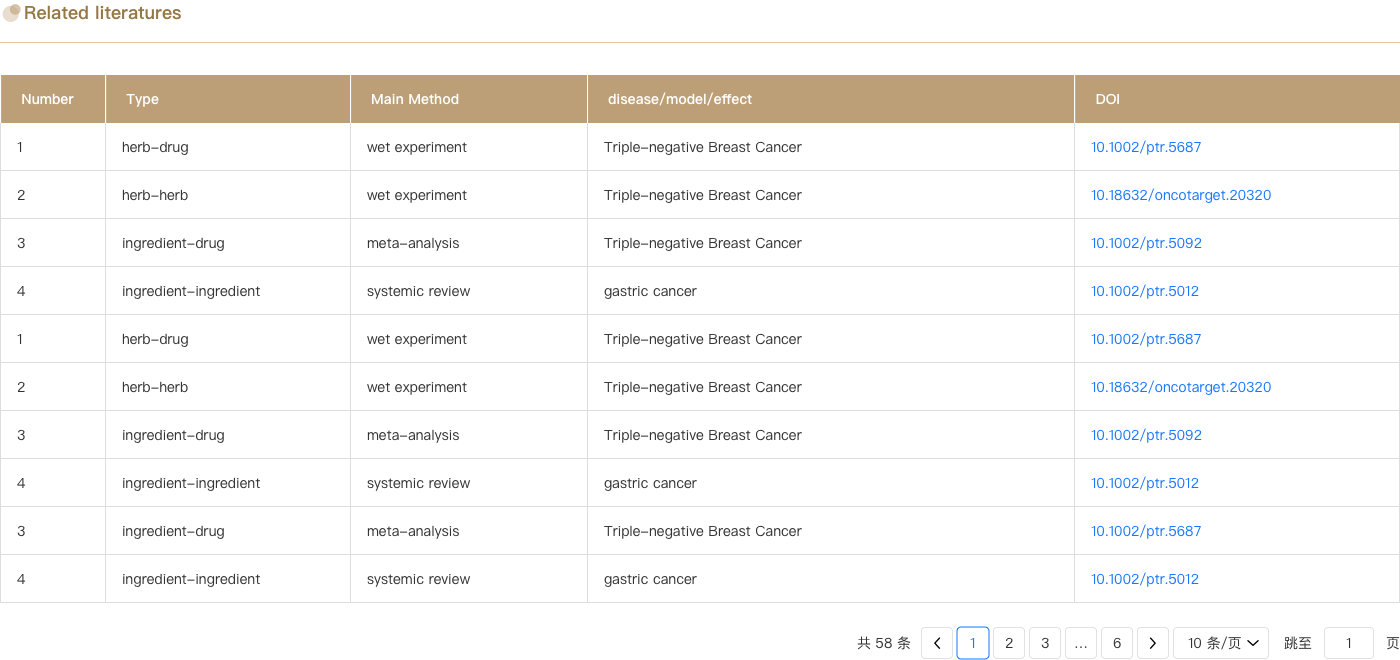


**Click to view the original text details of the Chinese and Western literature related to the ingredient.**


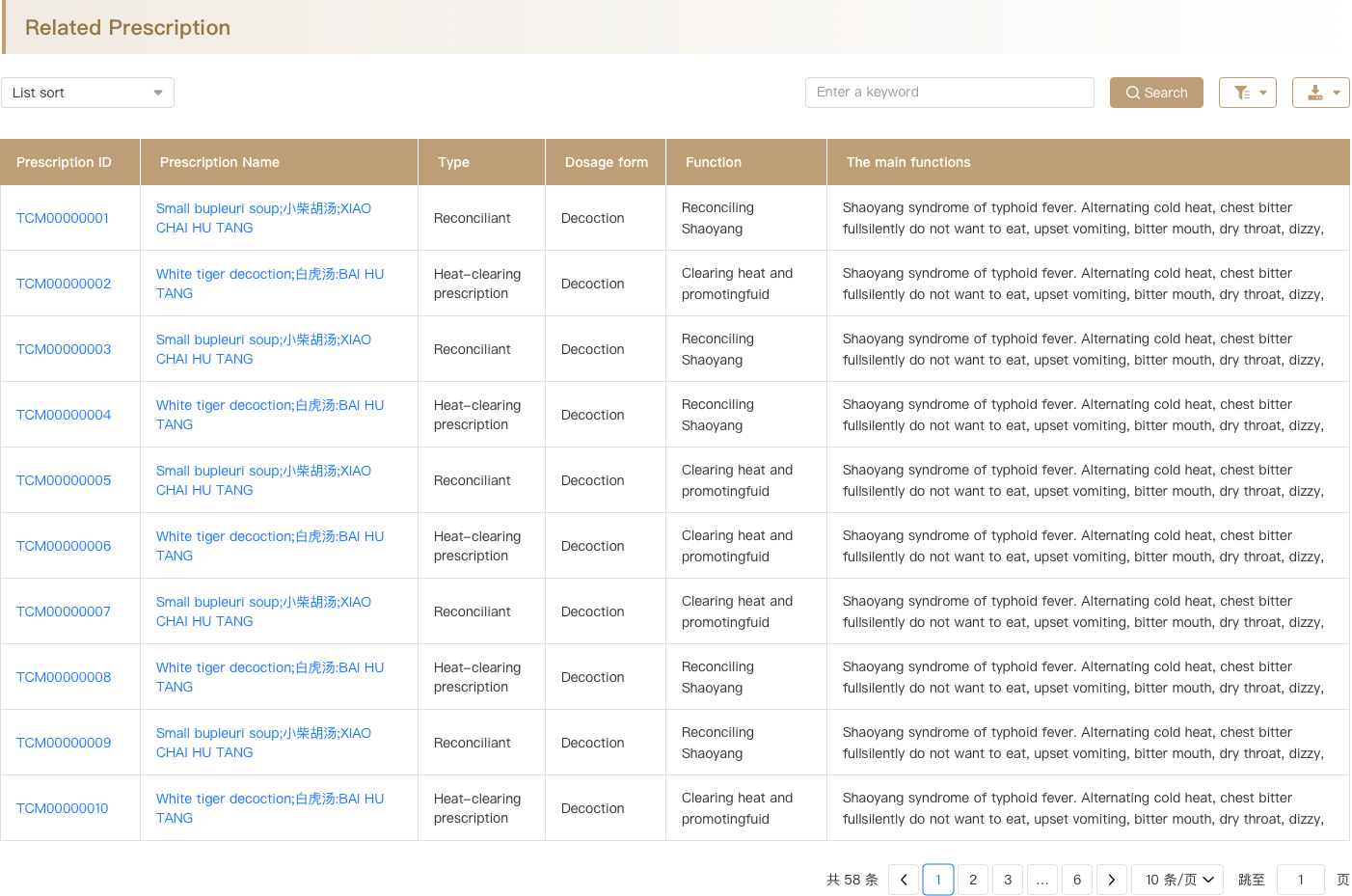


**Click to view the details of Herb prescriptions related to the ingredient.**


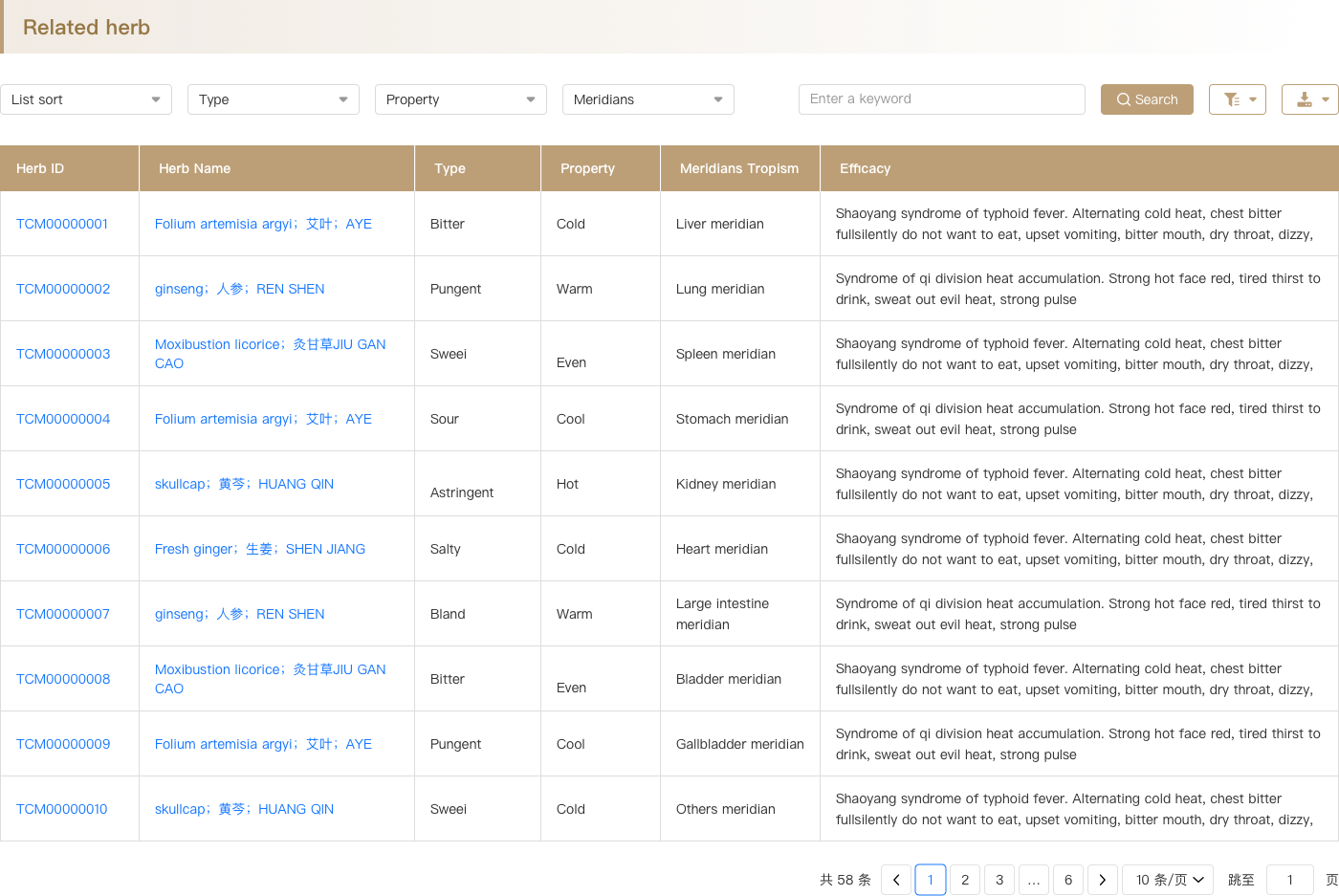


**Click to view the details of herbs related to the** **ingredient.**

## Tutorial for browsing Diseases in HerbComb

### Step 1: Users can browse the basic information of diseases and disease-related ingredients, as well as herbs and prescriptions associated with the diseases by clicking the disease section in the home page of HerbComb.


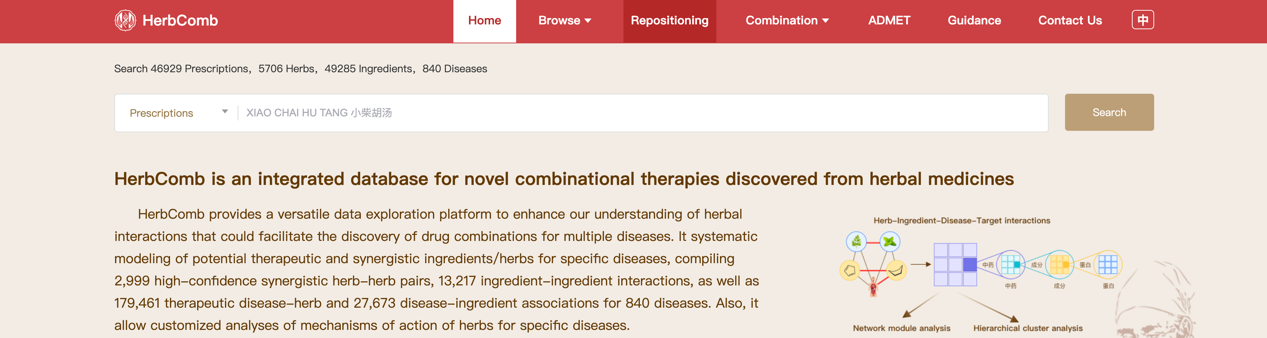


**Basic information of diseases**


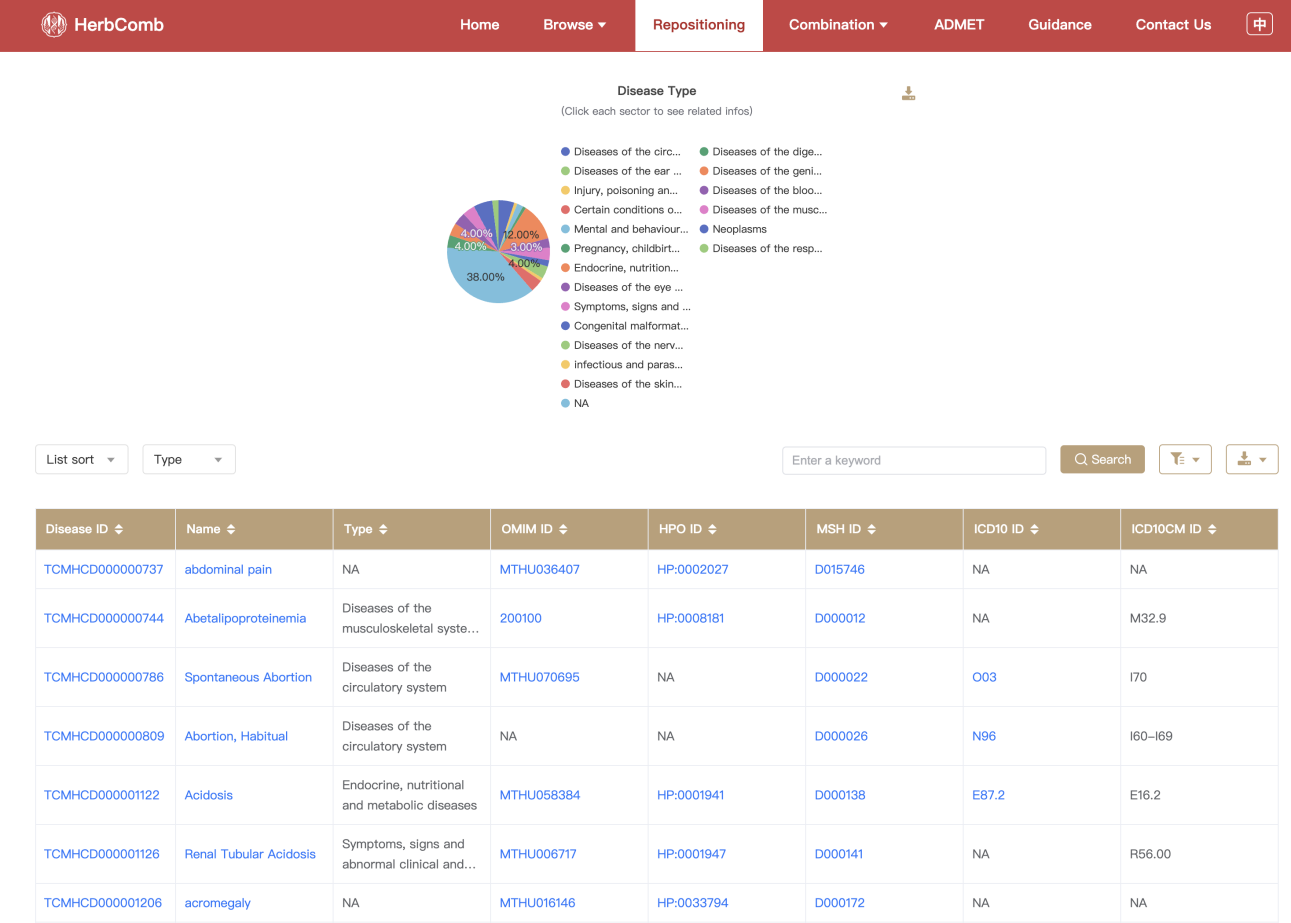


**Link to the detailed information of a disease.**

**Users can search a disease by its name，as well as  export and save the diseases list.**

**Users can select diseases according to their types.**

### Step 2: Users can browse the detailed information of the diseases, and also can link to the pages of disease-related ingredients, as well as herbs and prescriptions associated with the diseases by clicking the ingredients,herb and prescription names.


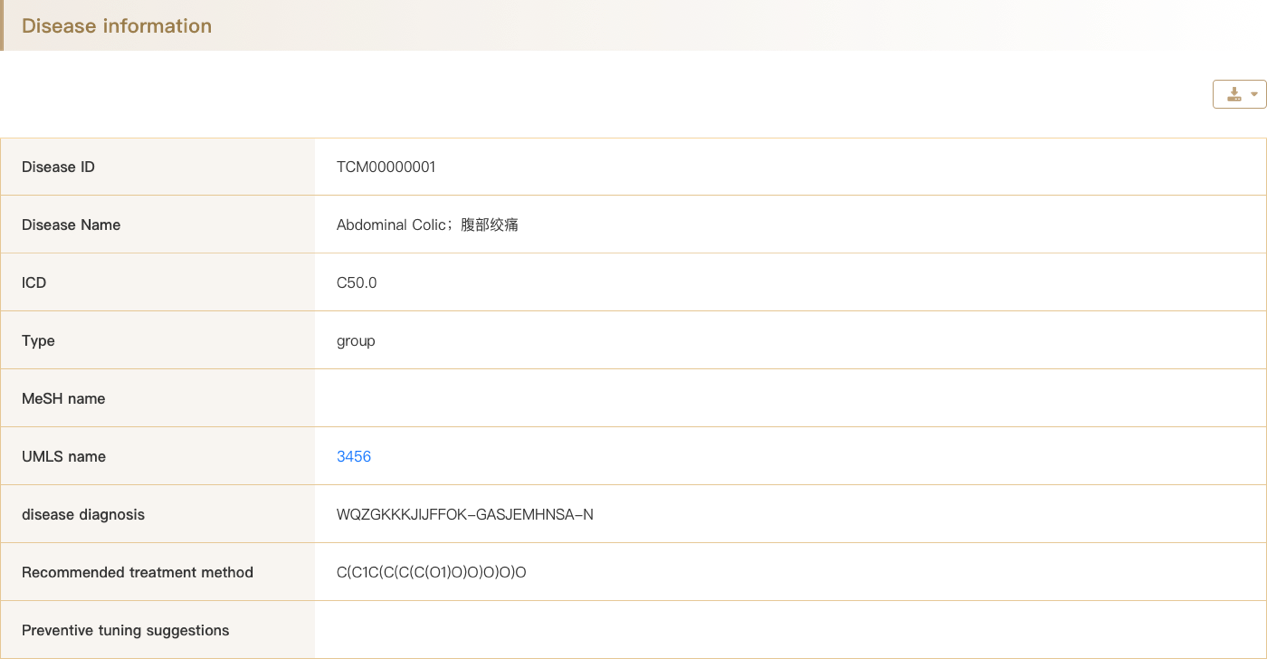


**Basic Information and Characteristics of disease.**


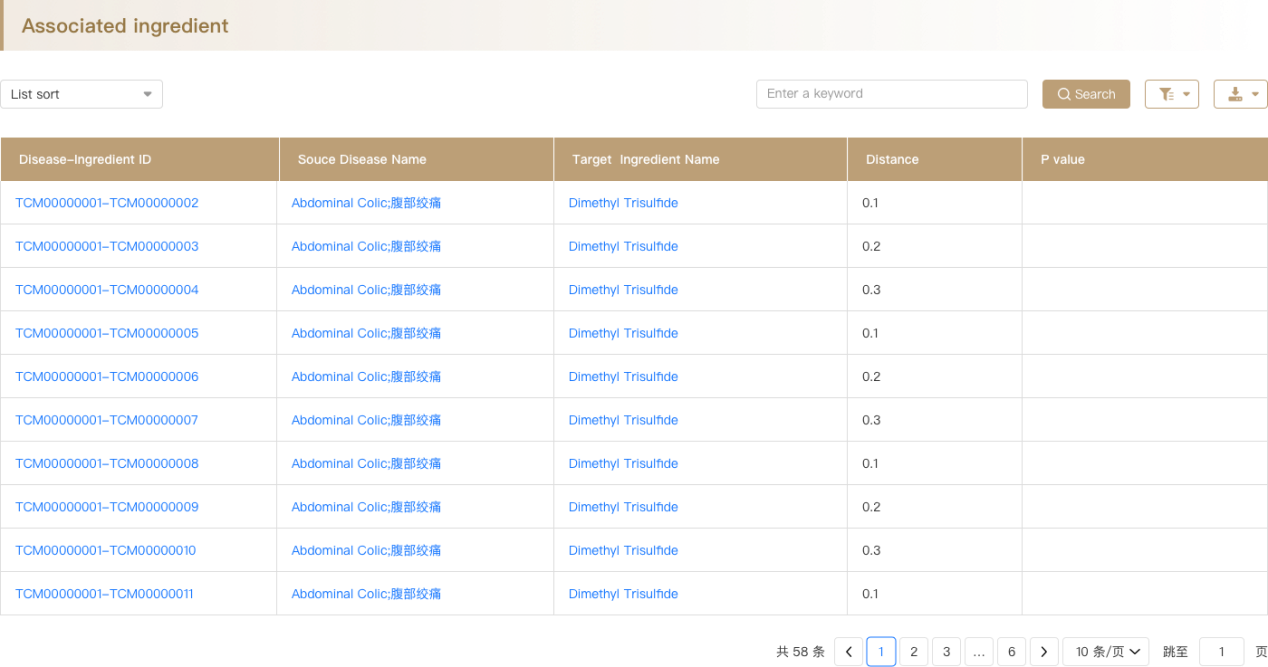


**Click to view the details of the related ingredient.**

**Click to navigate to the Combinatorial Analysis5: A Disease-ingredients’ page, to view the details of the network distance relationship chart between the disease and a ingredient.**

**
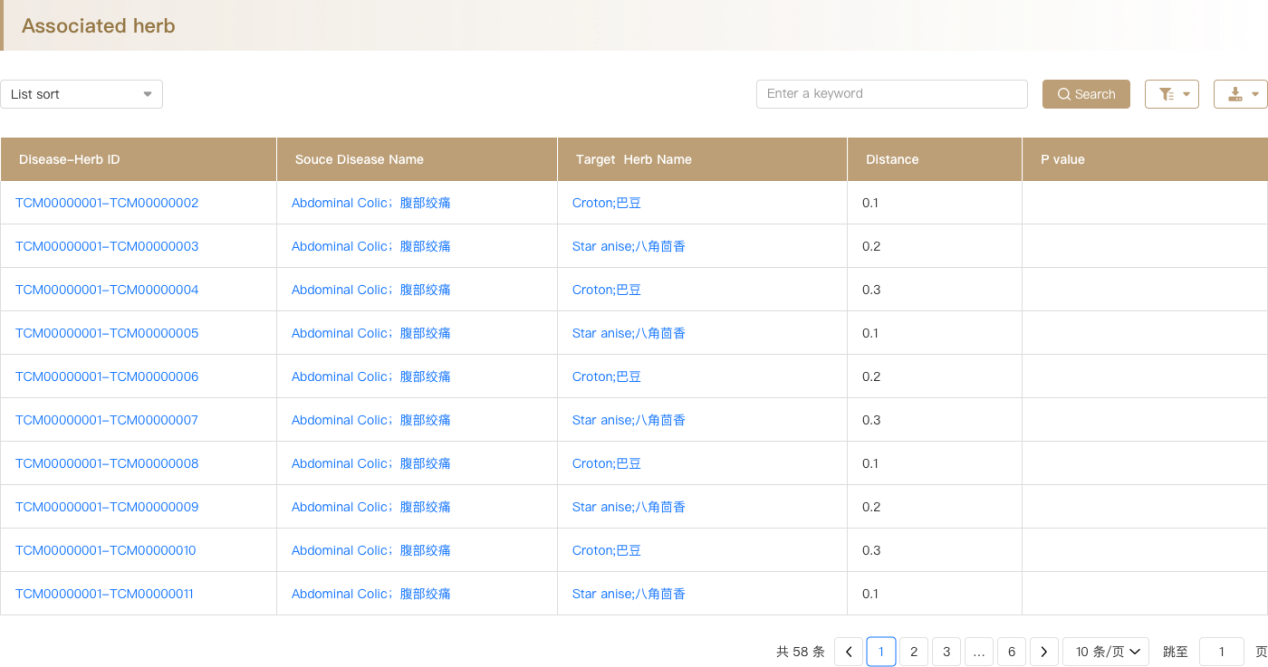
**

**Click to view the details of the related herb.**

**Click to navigate to the Combinatorial Analysis4: A Disease-herbs’ page, to view the details of the network distance relationship chart between the disease and a herb.**

**Click to view the details of the related Herb prescription.**


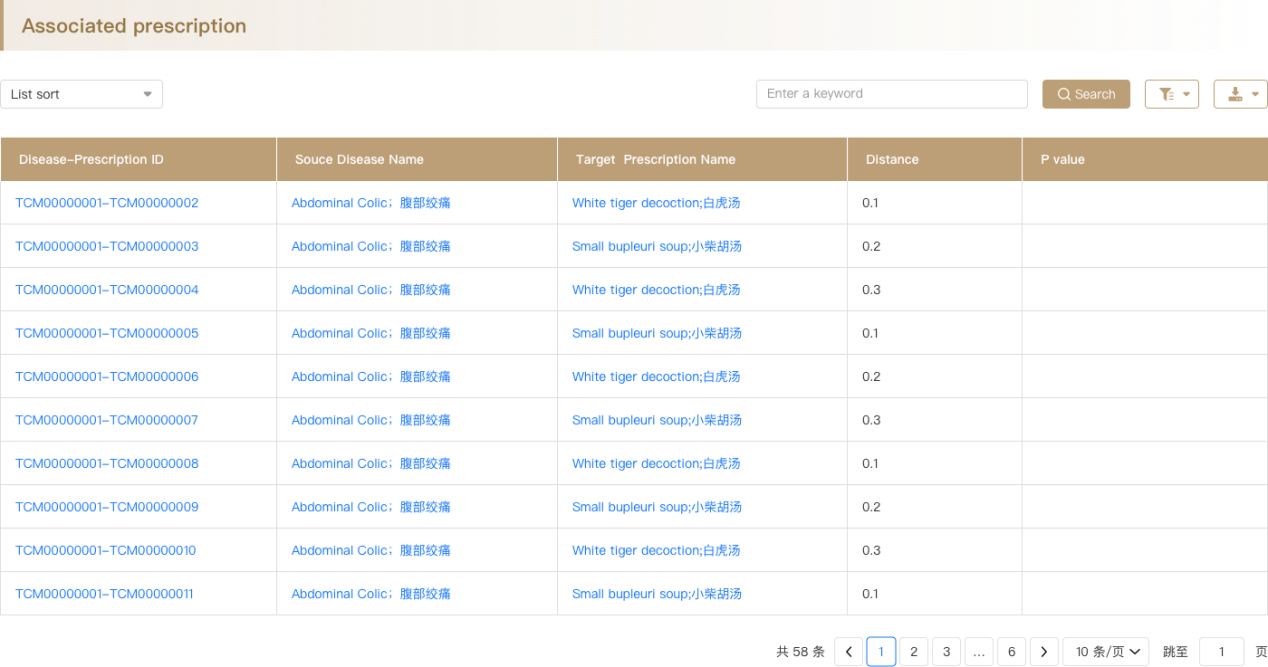


**Click to navigate to the Combinatorial Analysis3: A Disease- Herb prescription page, to view the details of the network distance relationship chart between the disease and a Herb prescription.**

## Tutorial for searching a prescription, herb, ingredient and disease in HerbComb

### Method 1: Users may initiate a comprehensive search by entering keywords into the search box on the homepage and subsequently clicking the search button.


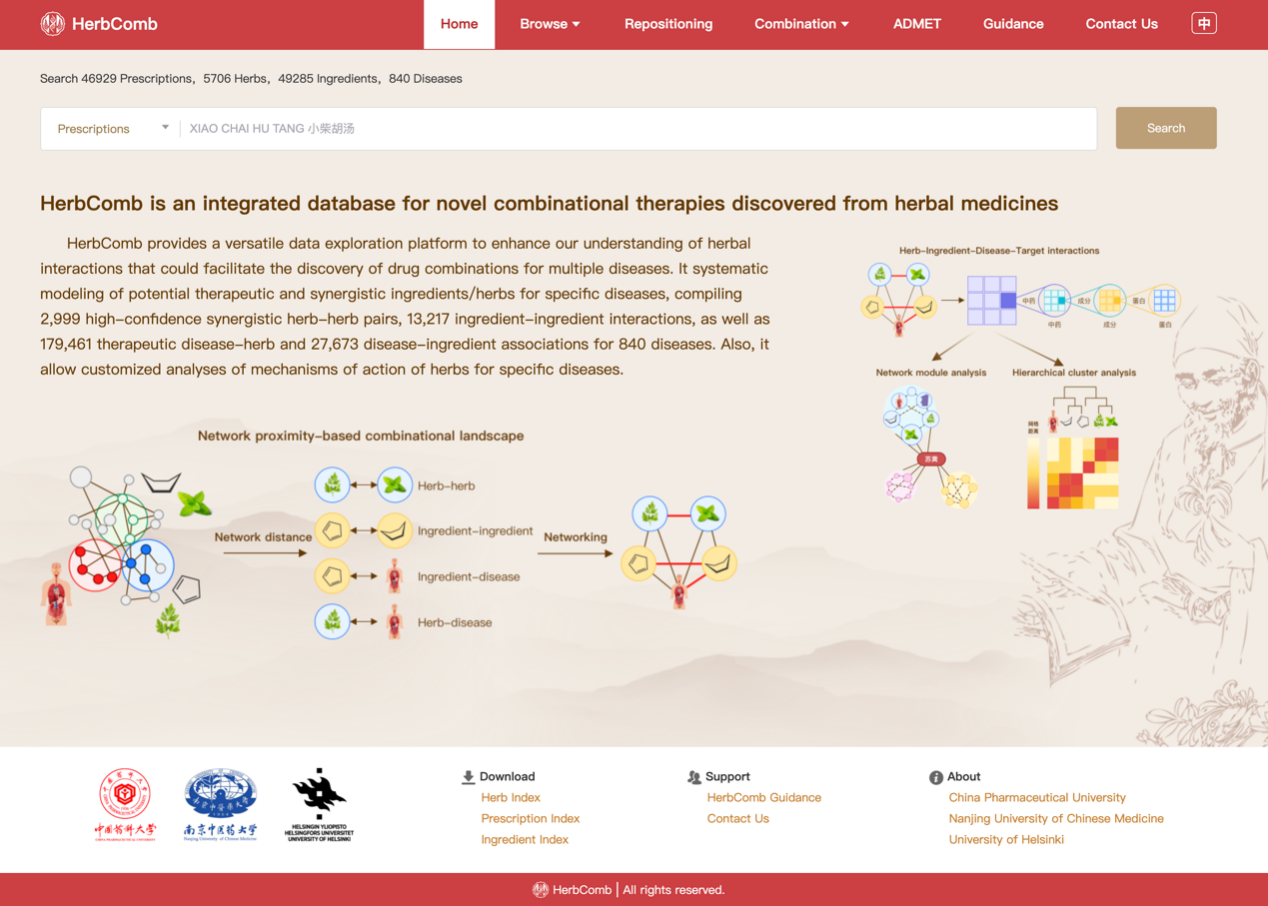


**Step 2，Click the search button, fuzzy search.**

**Step 1，Users directly enter the keywords they need to search for in the search box.**

### Method 2: Users may initially select a search category, key in relevant phrases, press the search button, and conduct their inquiry within the confines of the chosen category.


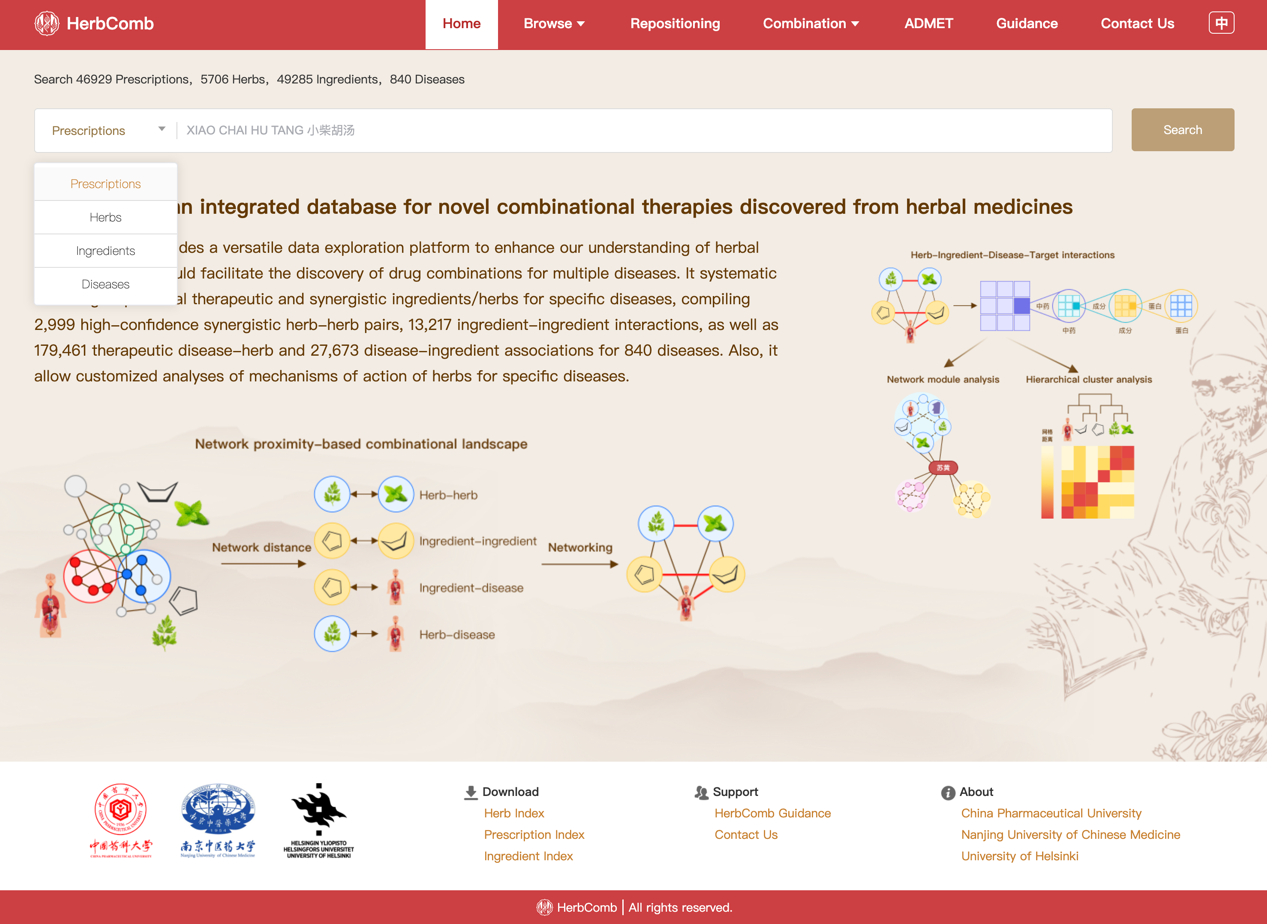


**Step 1，Users directly enter the keywords they need to search for in the search box.**

**Step 2，The user inputs the keywords into the search box.**

**Step 3，Click the search button to perform a fuzzy search within the category information.**

## Tutorial for systematic analysis on interactions among prescriptions, herbs, ingredients and a disease as users' selection

### Combinatorial Analysis1：herb-herb


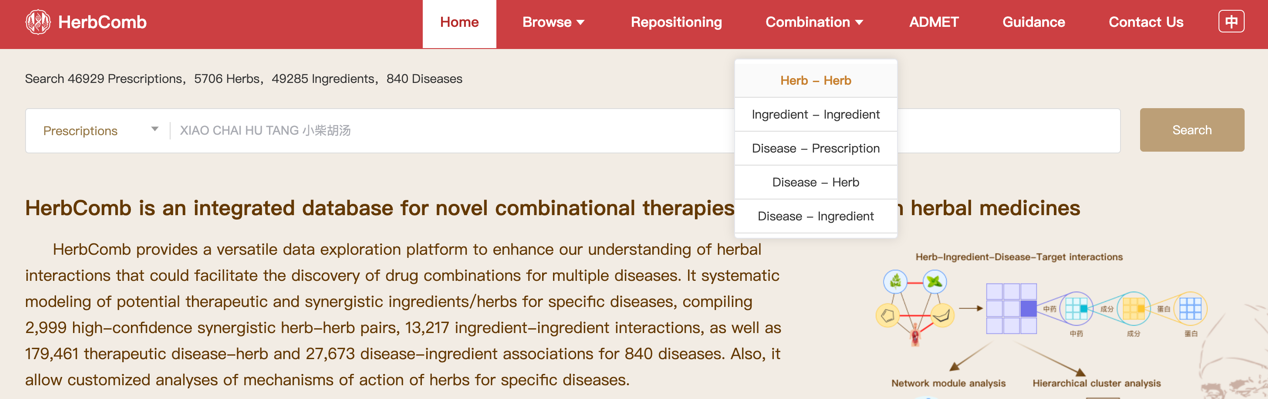


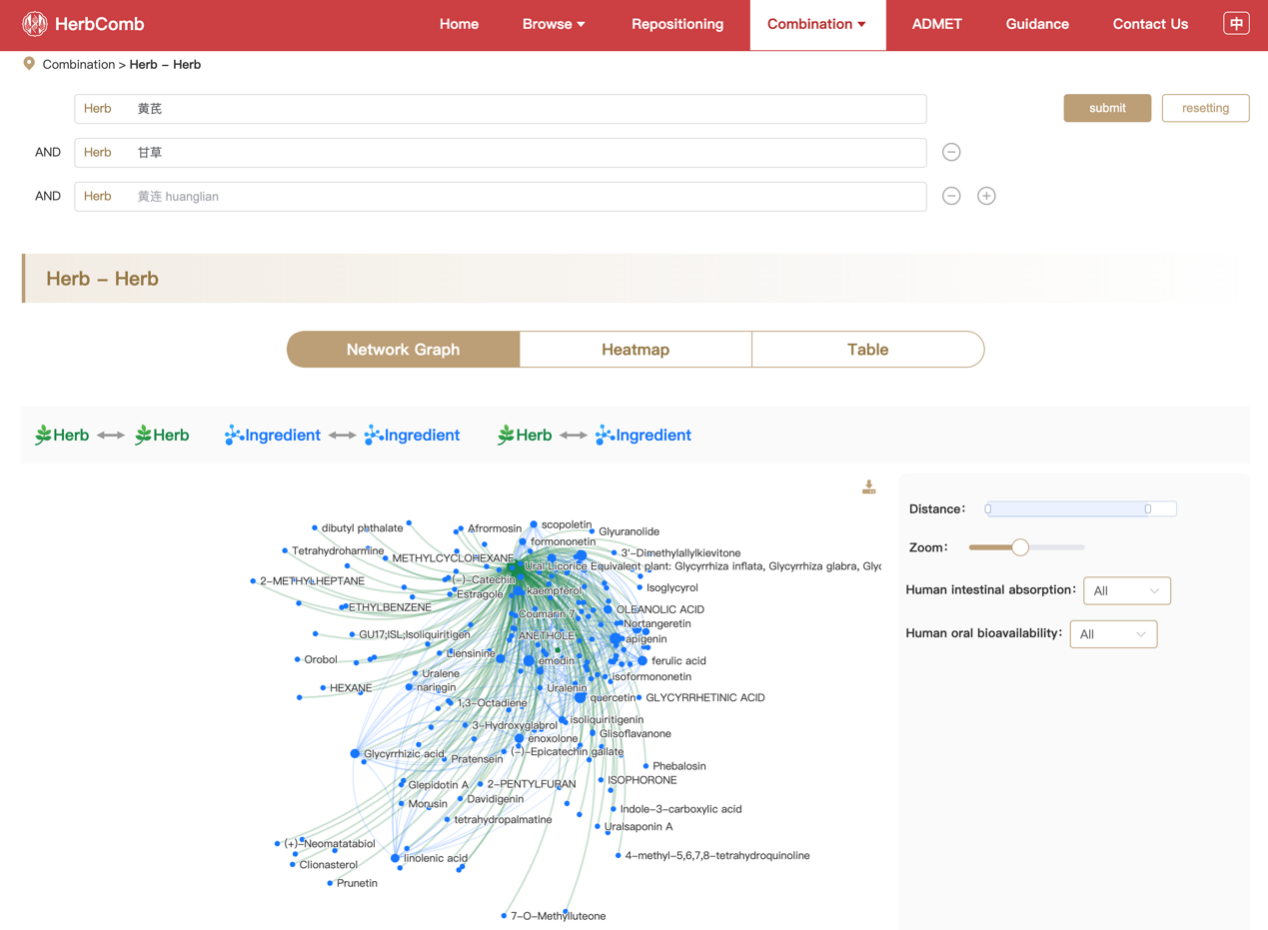


**Click on the relationship legend to view the related network distance relationships in combination.**

**Switch tabs to view the network graph, heatmap, and information list of herb-herb combinations.**

**Enter the names of the herbs required for combination analysis.**


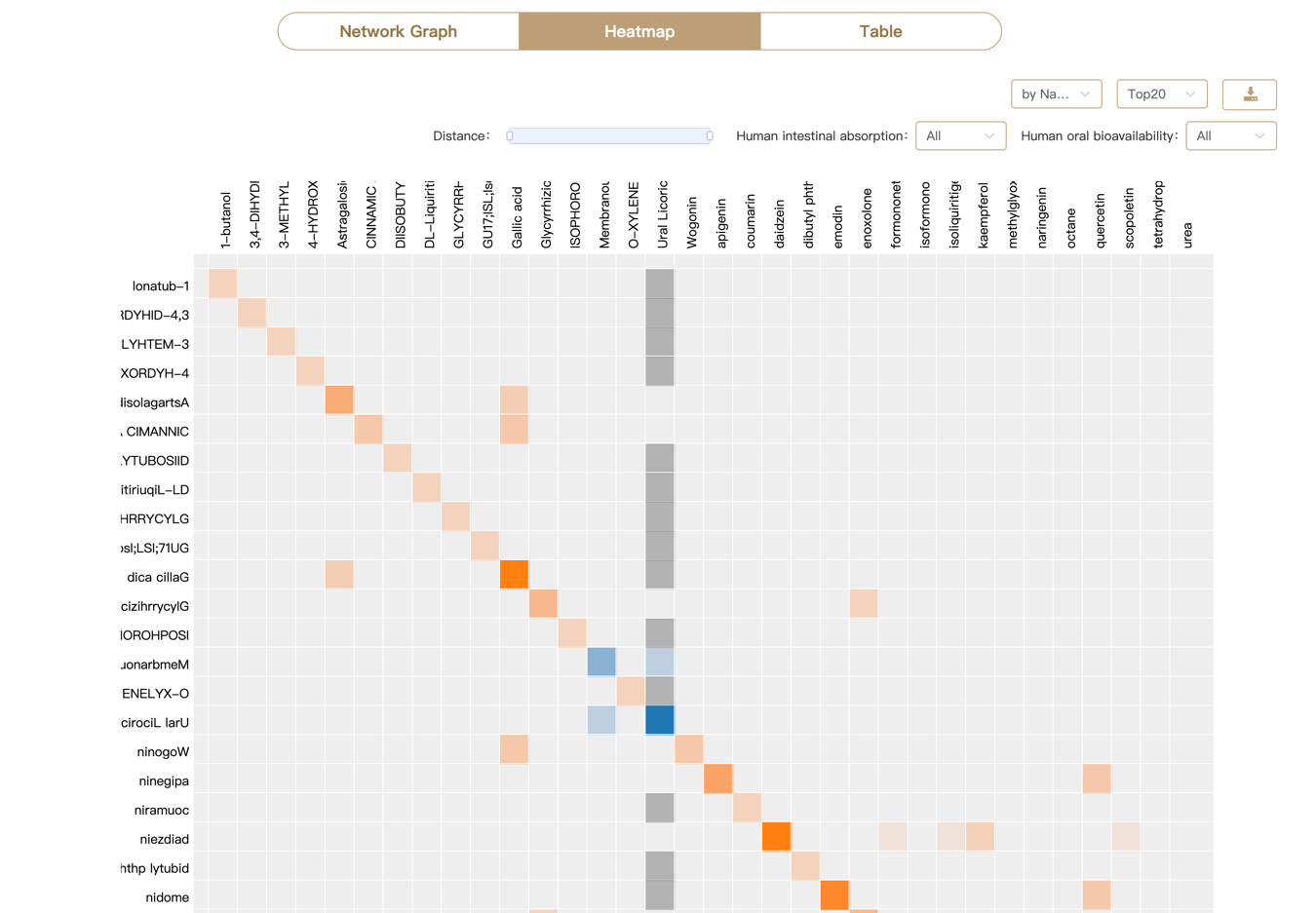


**Herb-herb combination heatmap, available for download.**


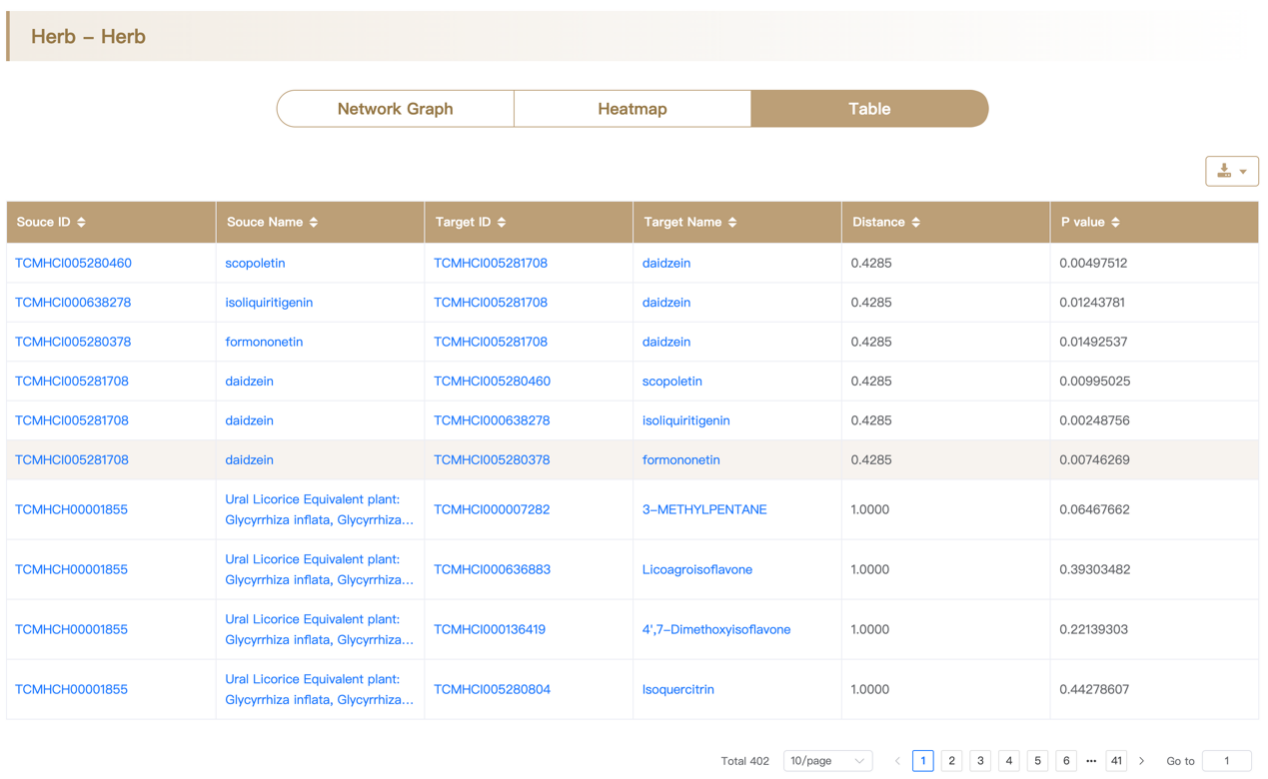


**Herb-herb combination list information, available for download.**

### Combinatorial Analysis2: Ingredient – Ingredient


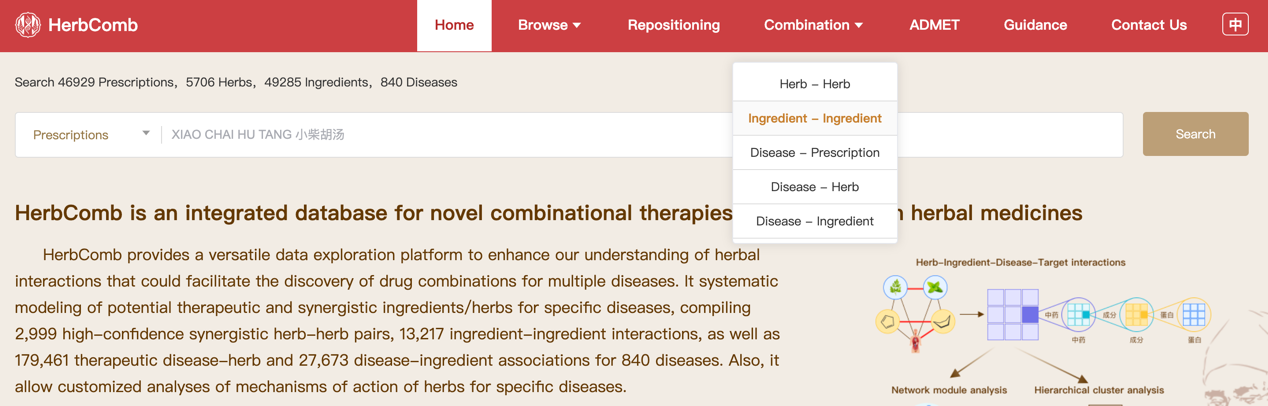


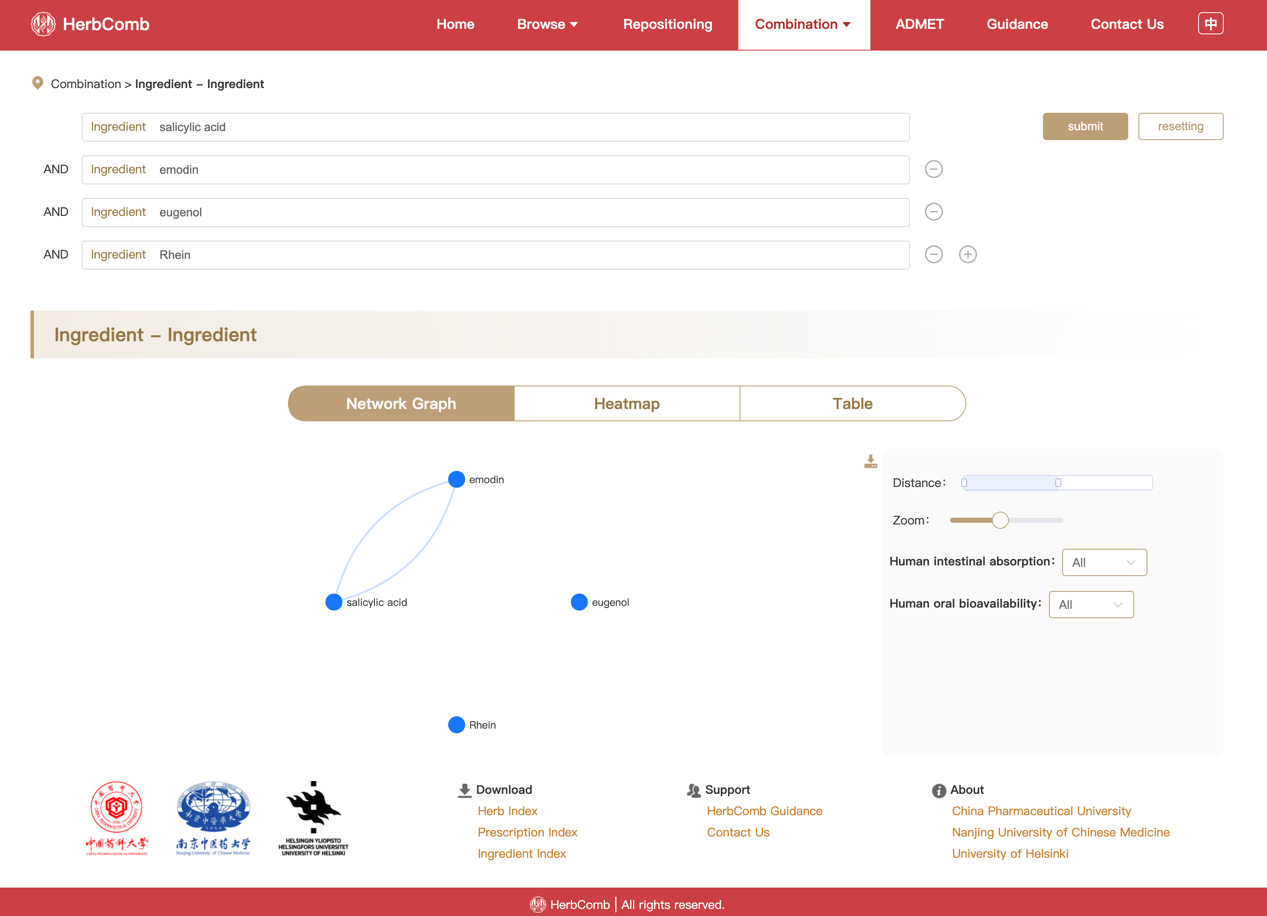


**Switch tabs to view the network diagram, heatmap, and information list for ingredient-ingredient combinations.**

**Enter the names of the ingredients to be combined for viewing.**


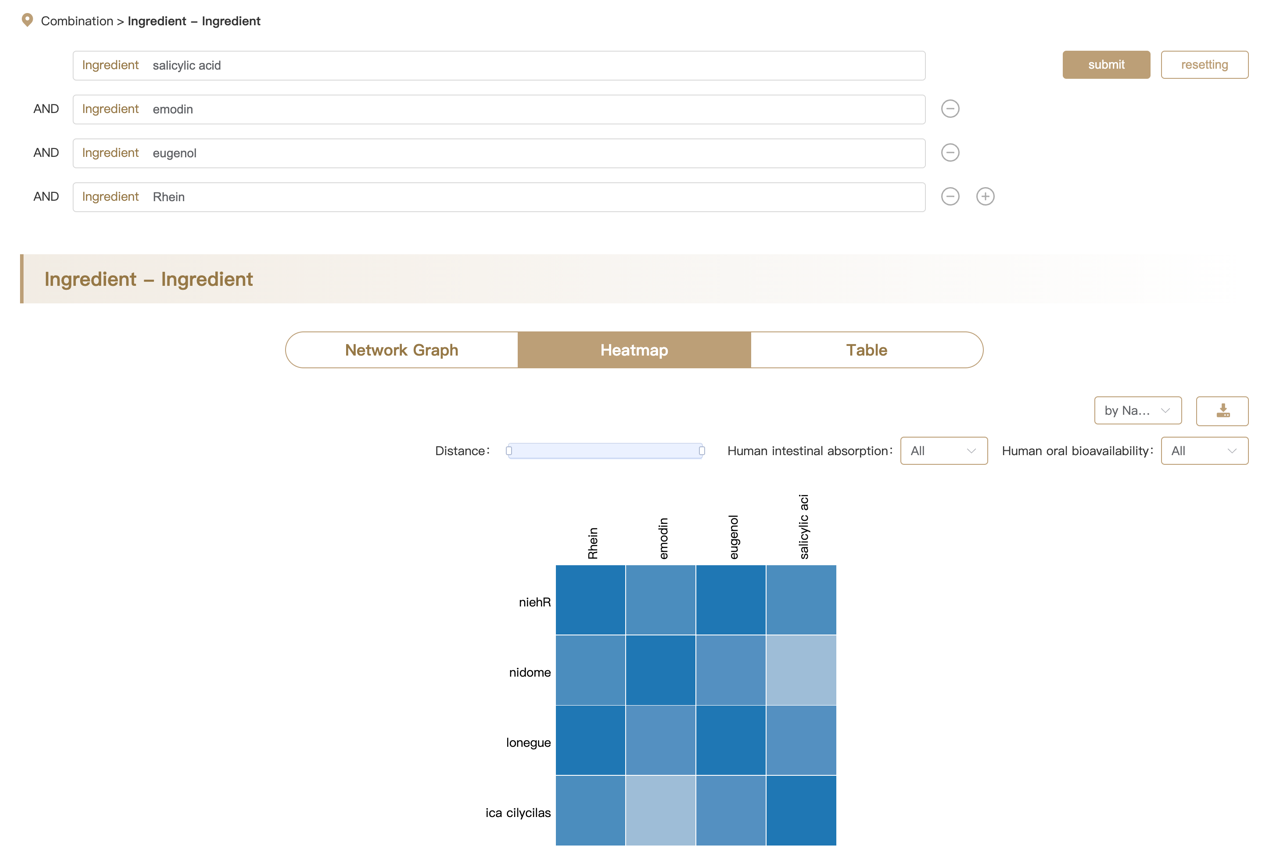


**Ingredient-ingredient combination heatmap, downloadable.**

**Ingredient-ingredient combination information list, downloadable.**


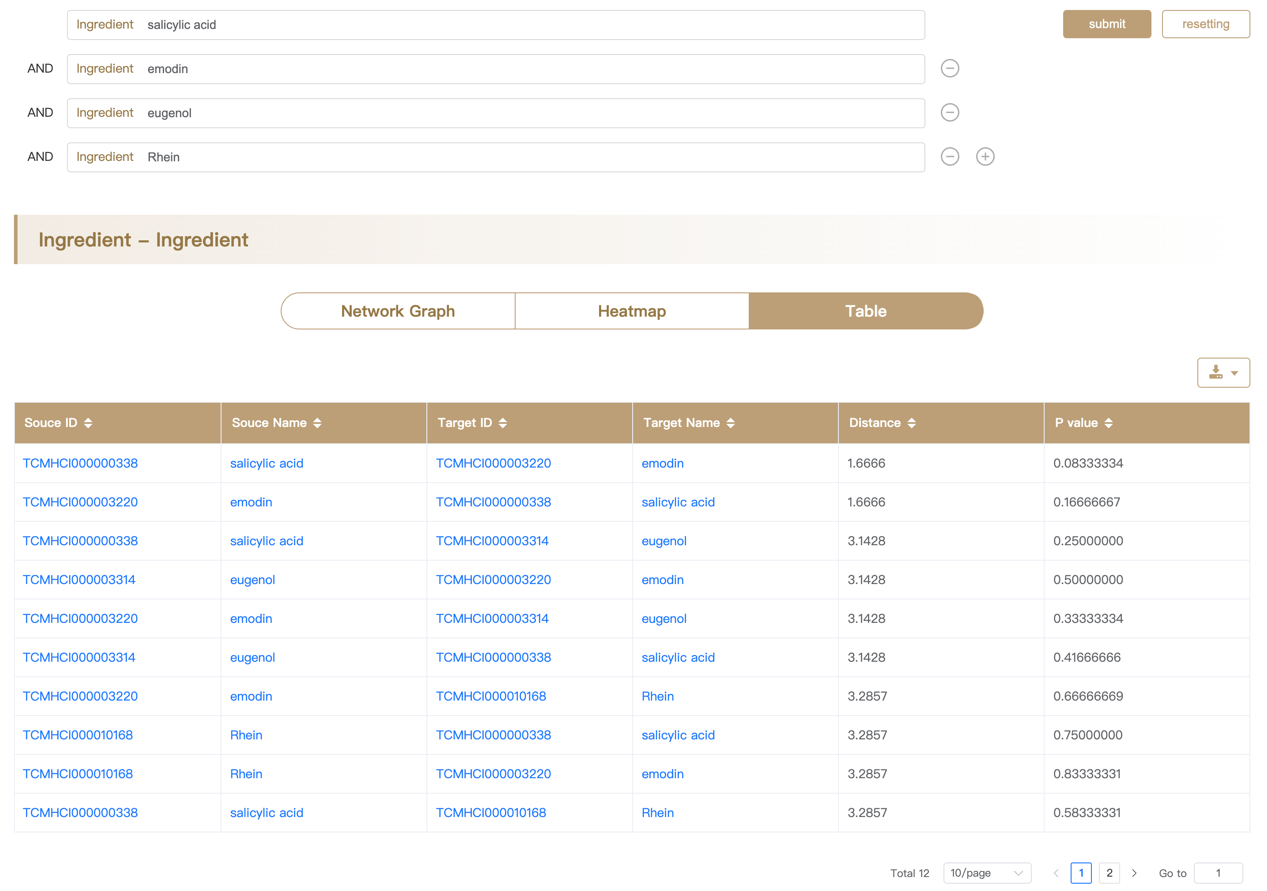


### Combinatorial Analysis3: A Prescription – Disease


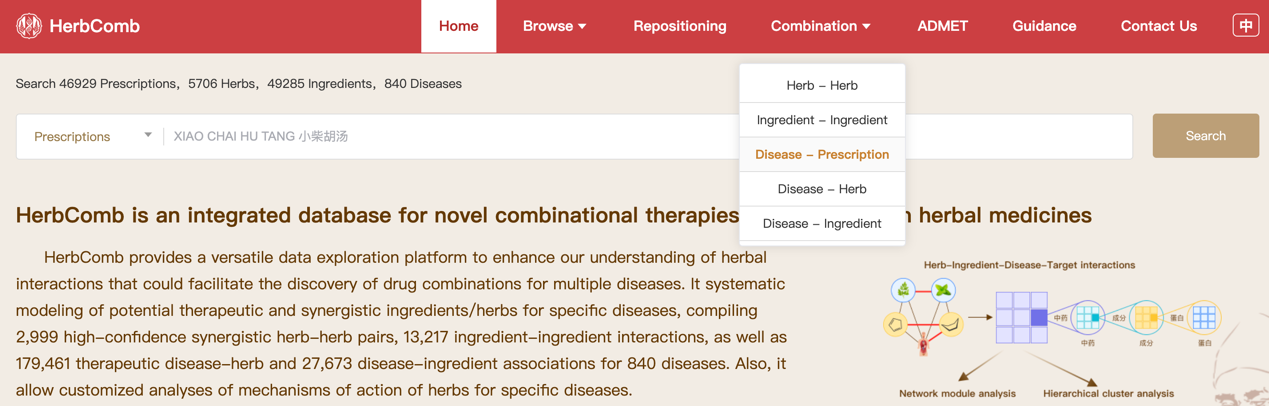


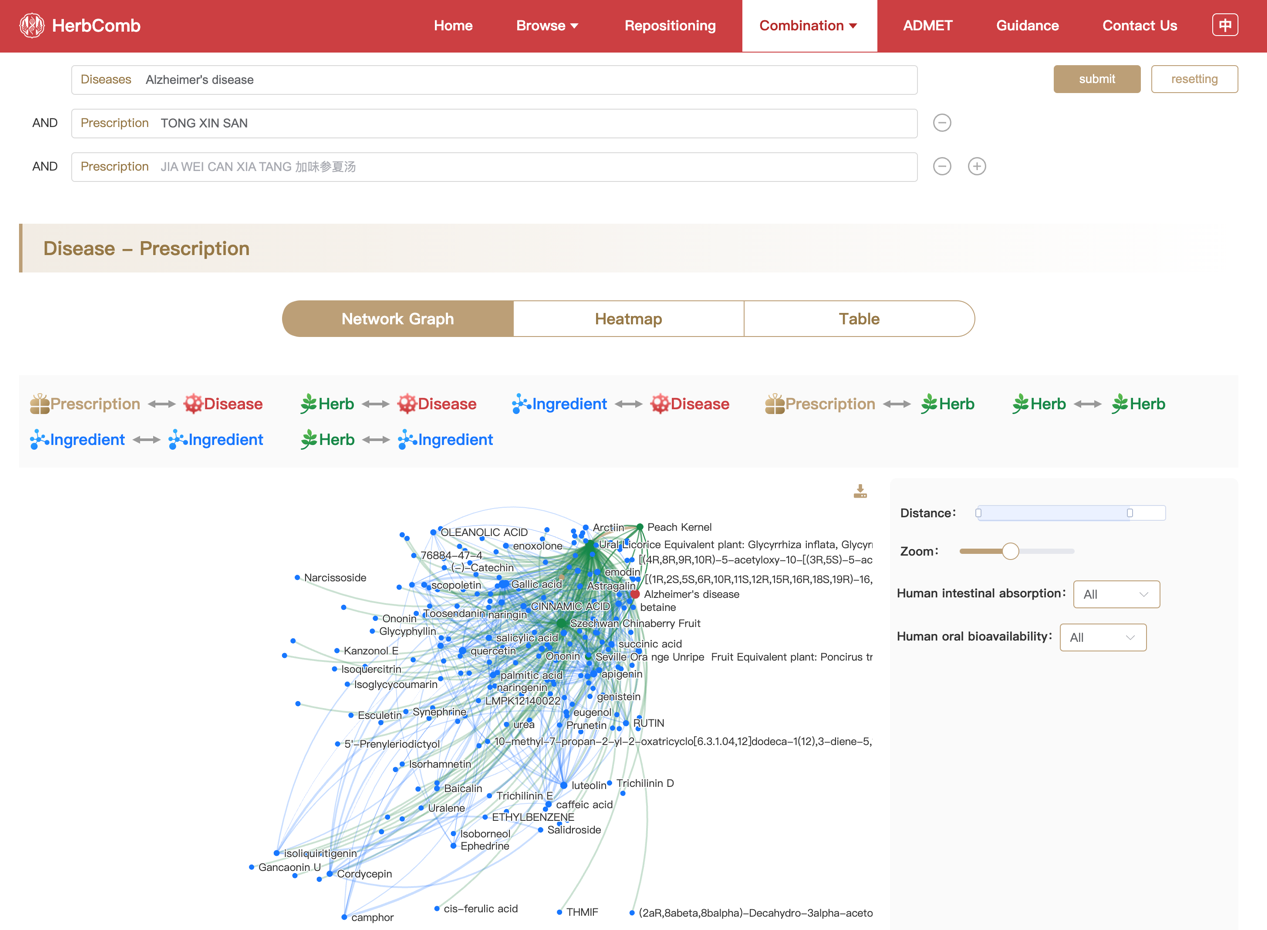


**Click on the relationship legend to view the related network distance relationships in combination.**

**Switch tabs to view the network graph, heat map, and information list of disease-Herb prescription combinations.**

**Enter the disease-Herb prescription name the user wish to see in combination.**


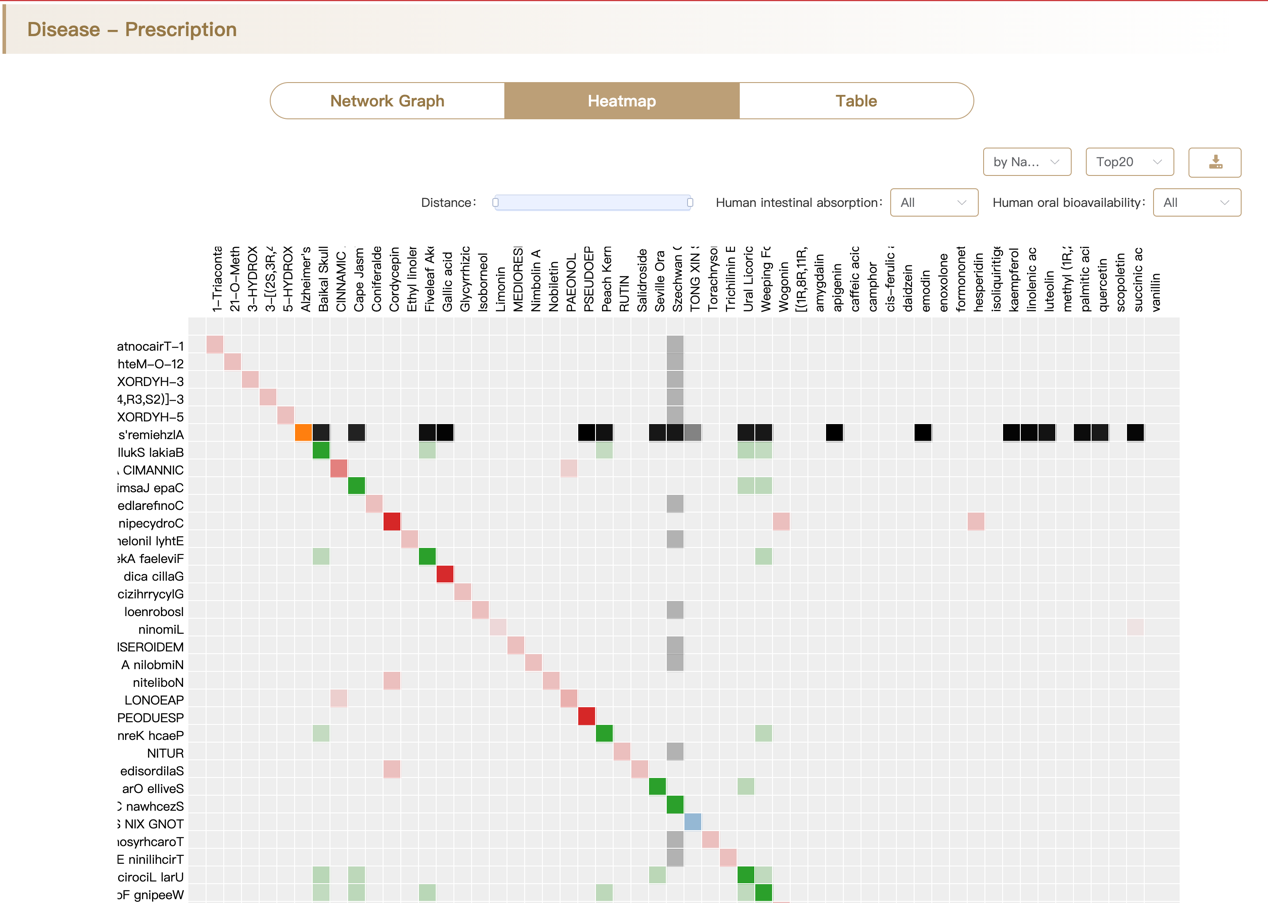


**Disease-Herb prescription combination heat map, downloadable.**


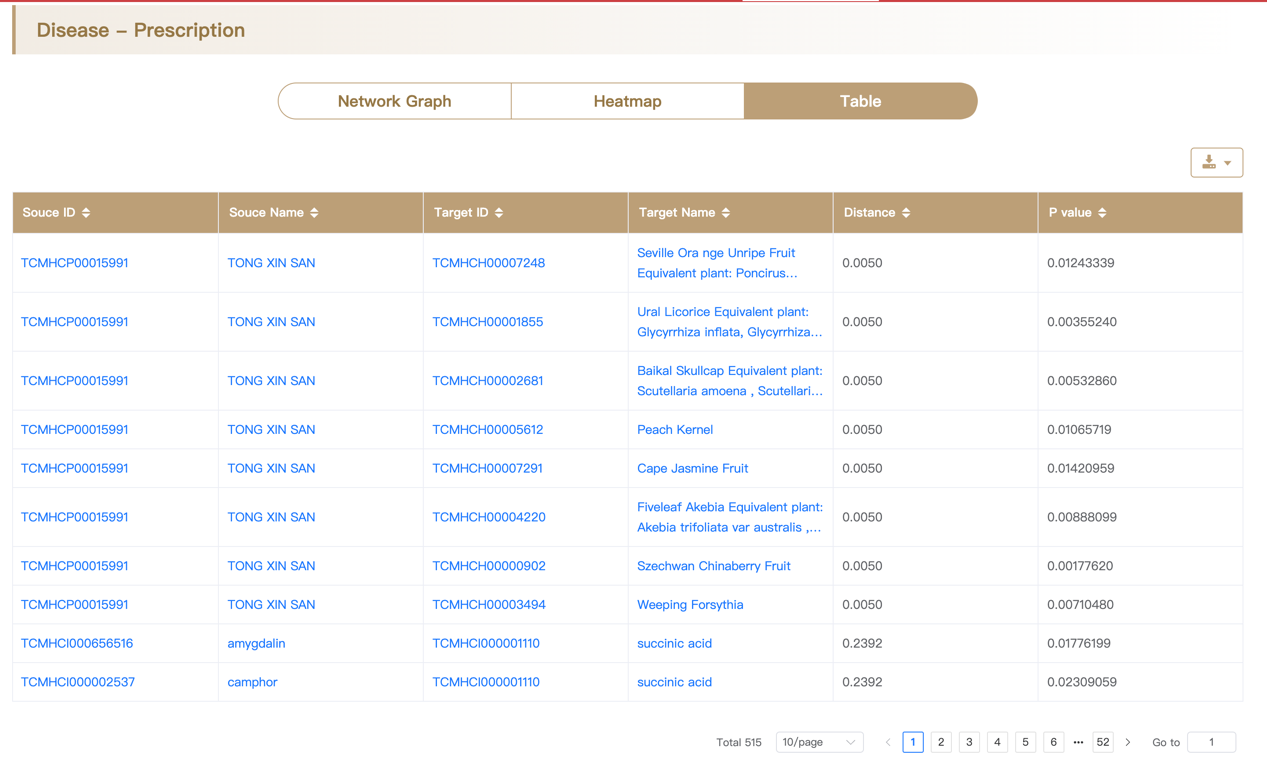


**Disease-Herb prescription combination list information, downloadable.**

### Combinatorial Analysis4: A Herbs – Disease


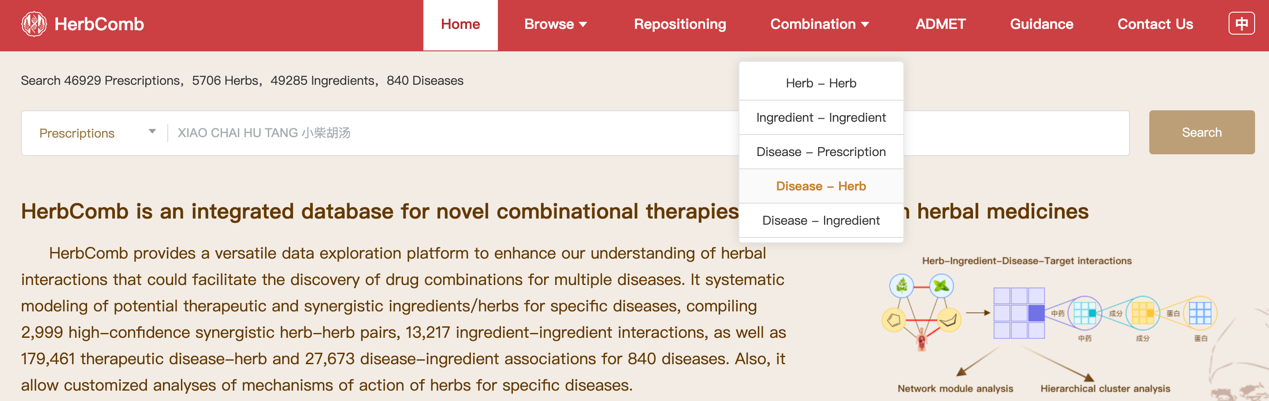


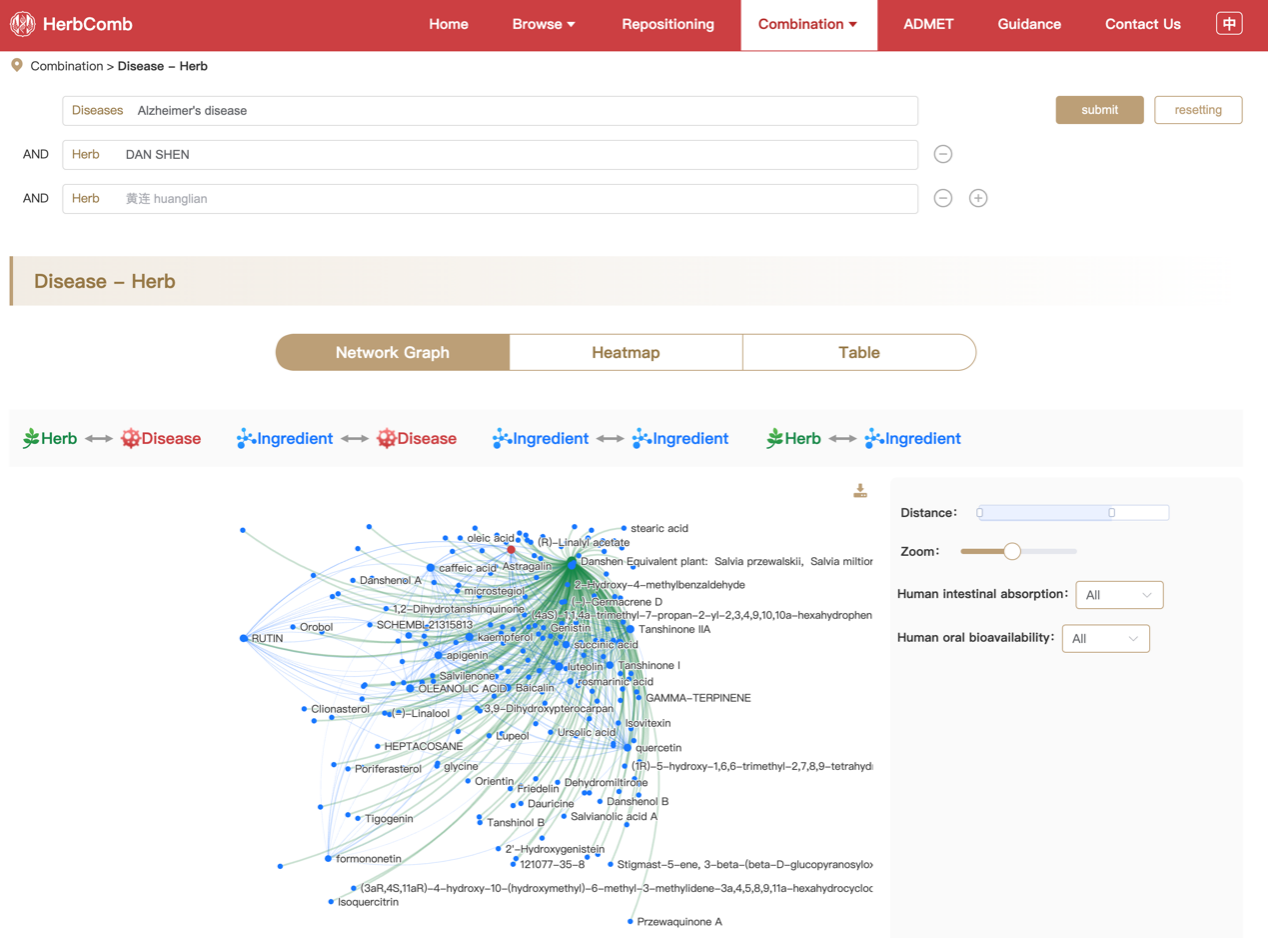


**Switch tabs to view the network graph, heatmap, and list information of disease-herb combinations.**

**Enter the disease-herb names to view in combination.**

**Click on the relationship legend to view the network distance relationships in combination.**


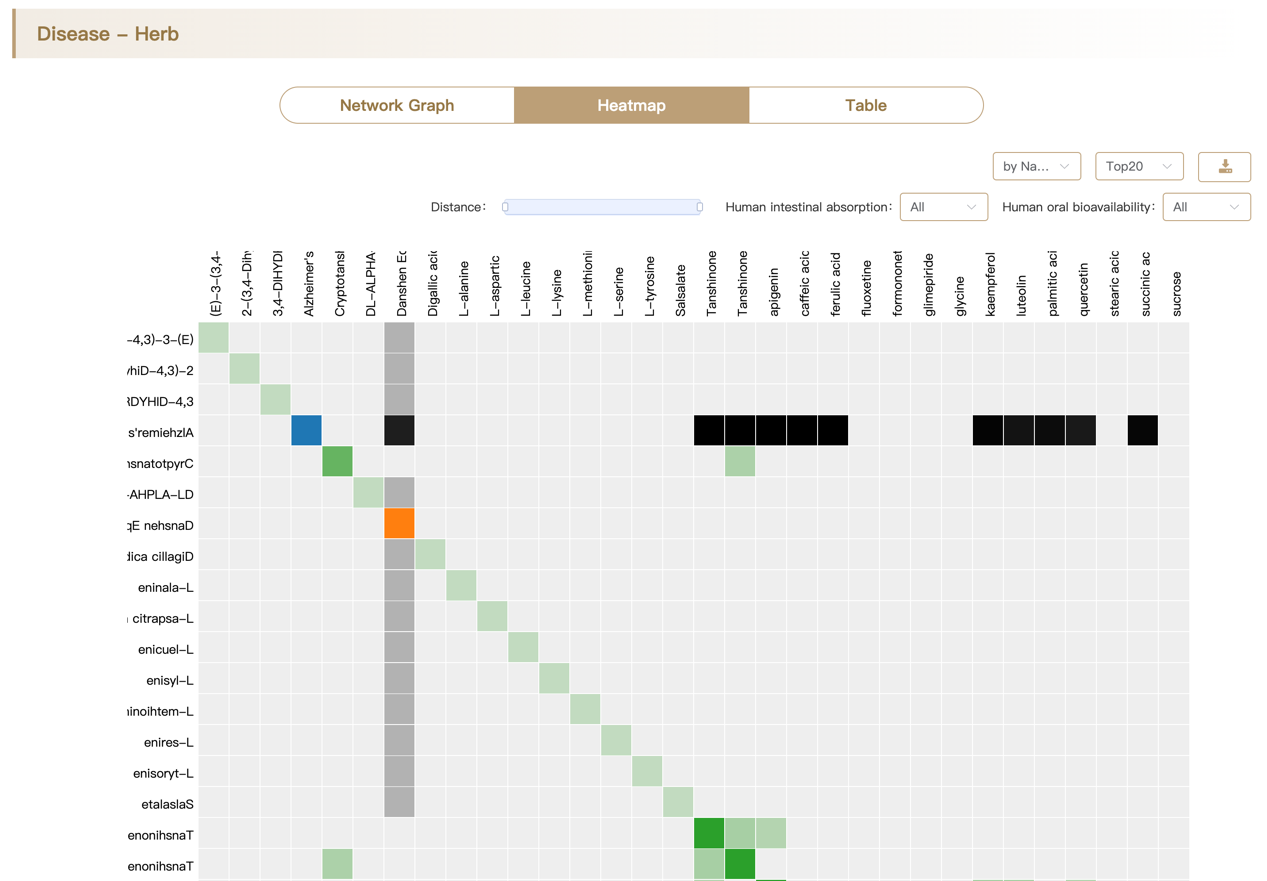


**A Disease-herbs Combination’s Heatmap, Downloadable.**


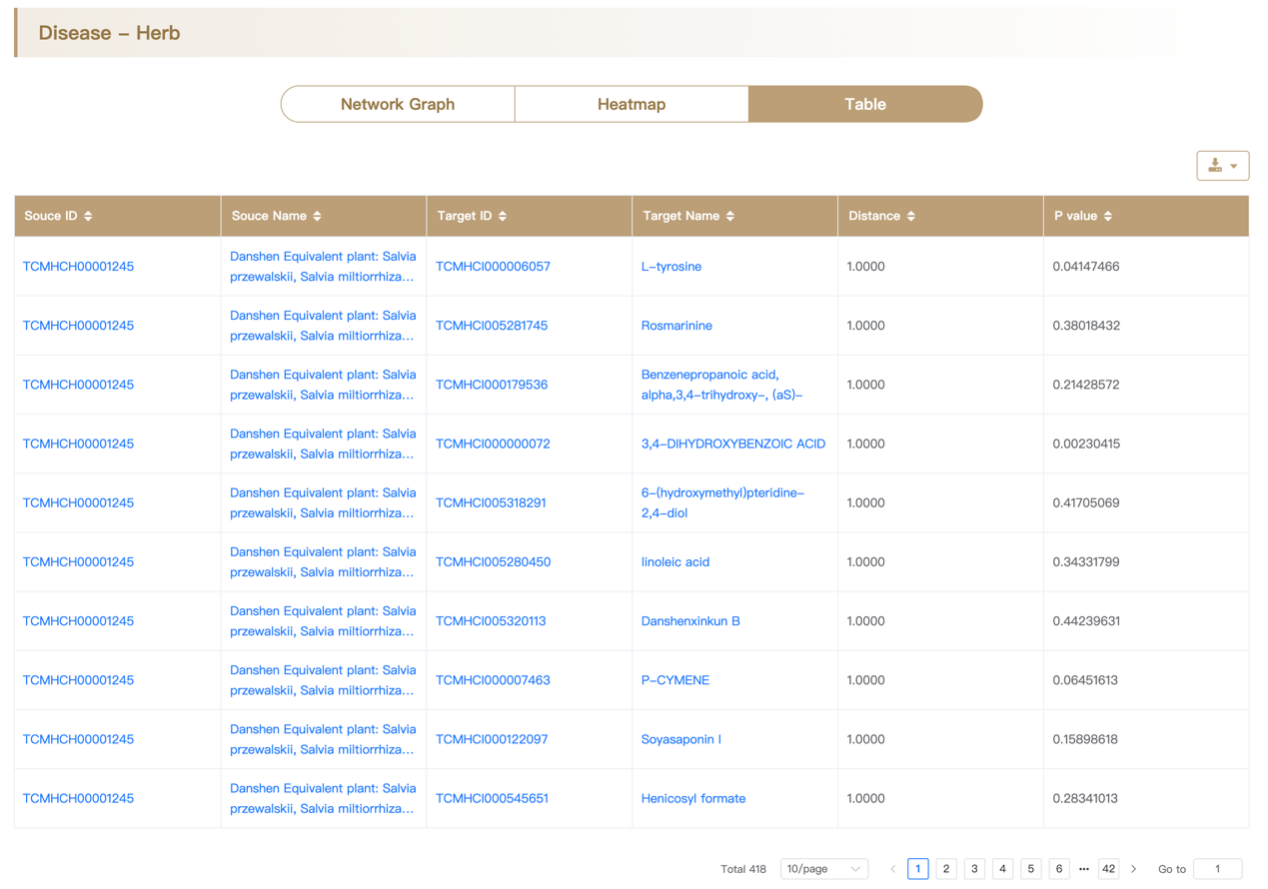


**A Disease-herbs Combination List Information, Downloadable.**

### Combinatorial Analysis5: A Disease-ingredients


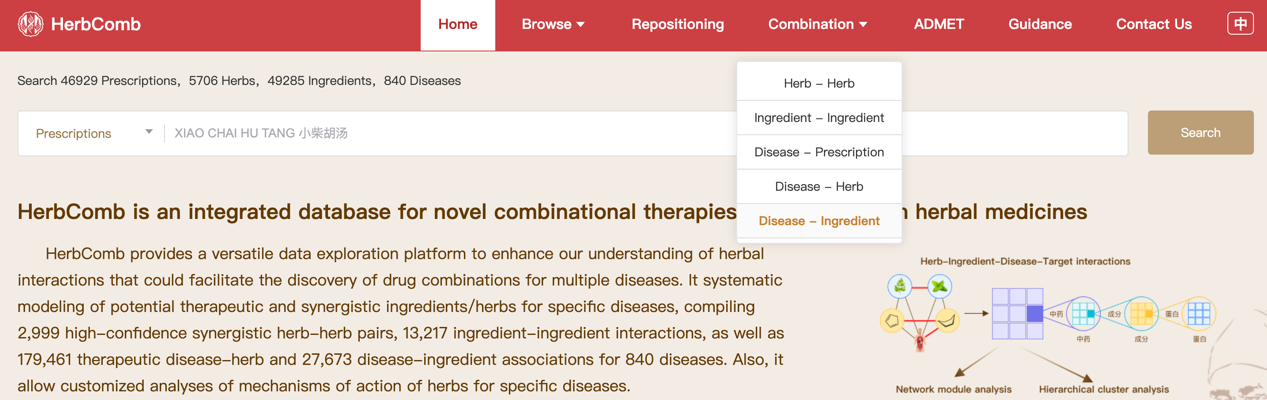


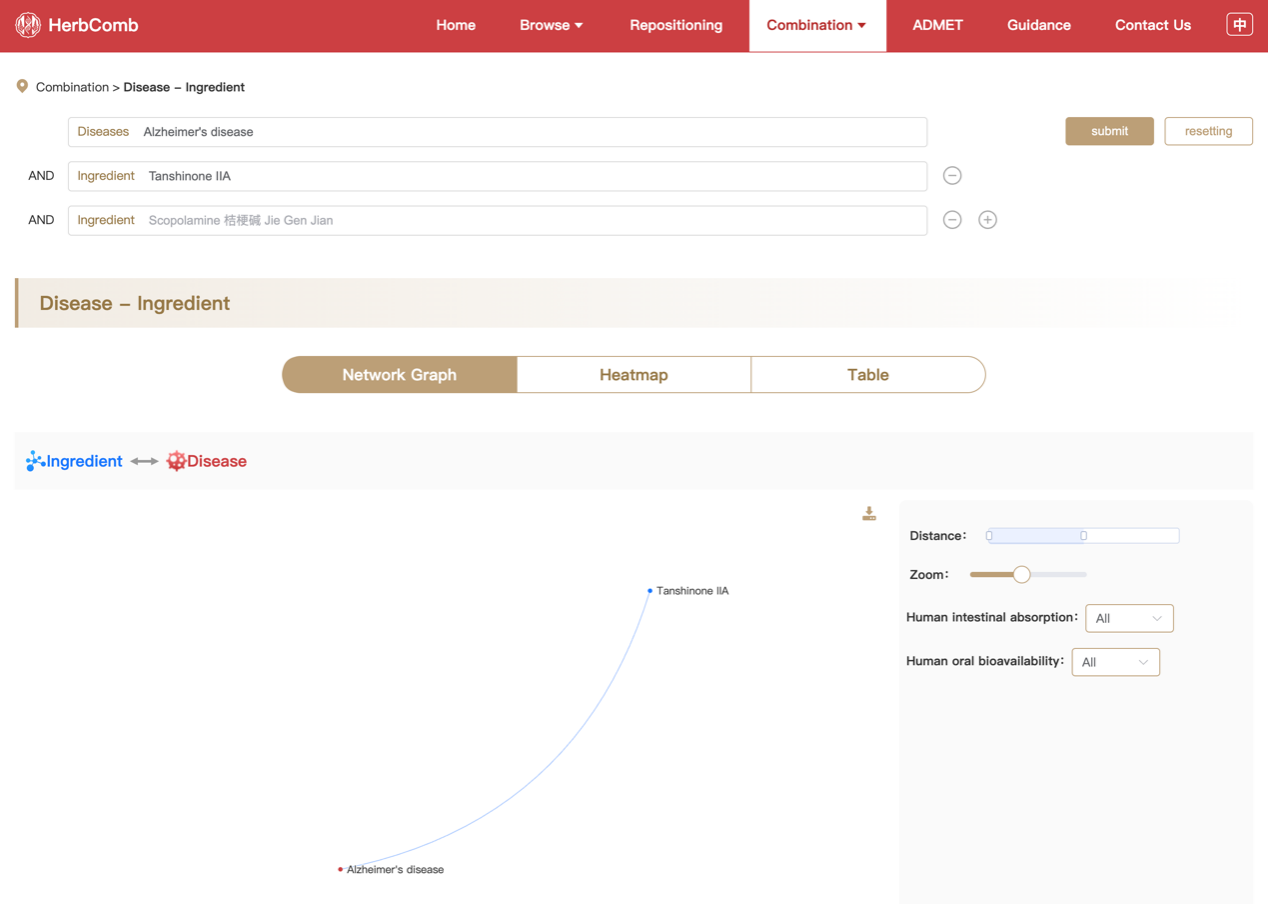


**Click on the relationship legend to combine and view the related network distance relationships.**

**Switch tabs to view the network diagram, heatmap, and information list of A Disease-Ingredients combinations.**

**Enter the names of A Disease-Ingredients to view in combination.**


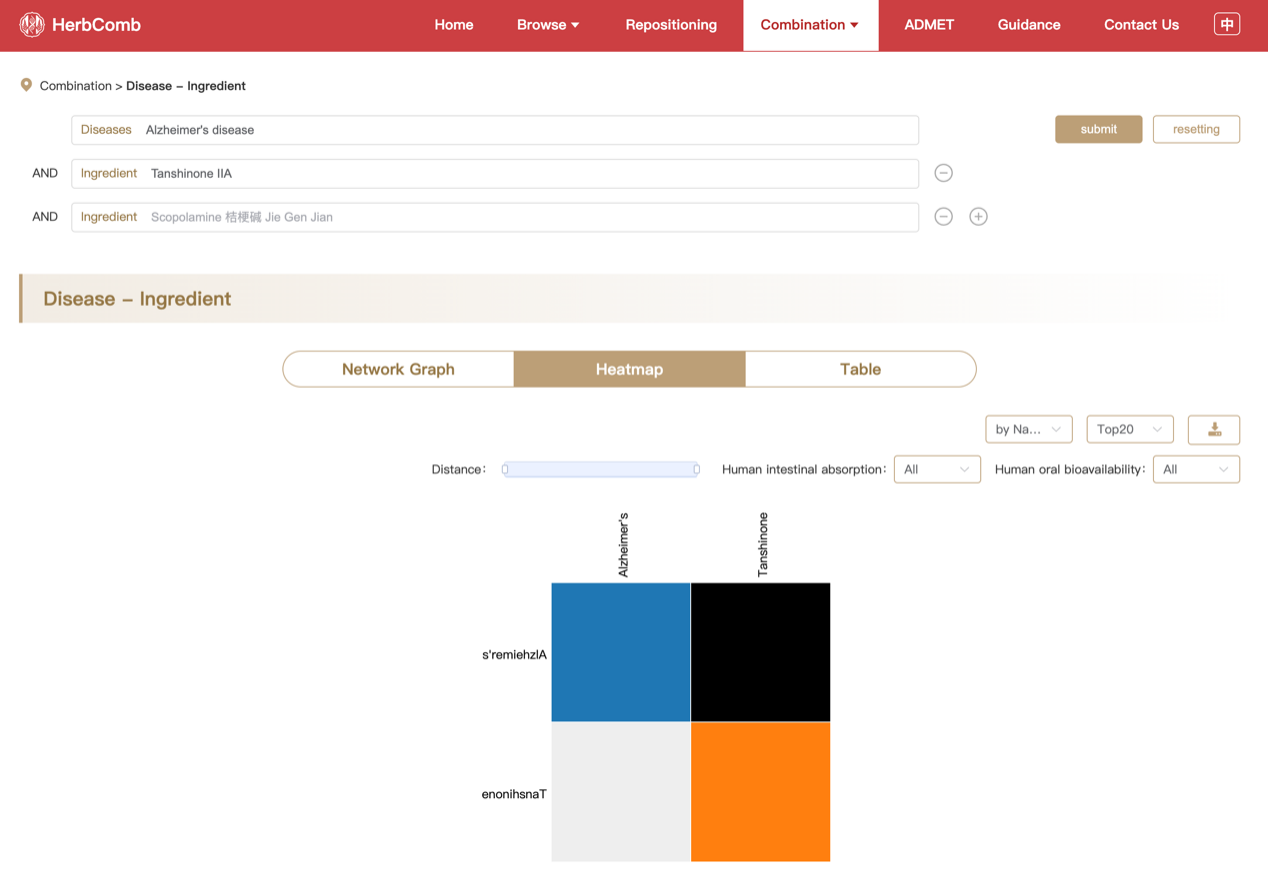


**A Disease-Ingredients Combination’s Heatmap, Downloadable.**


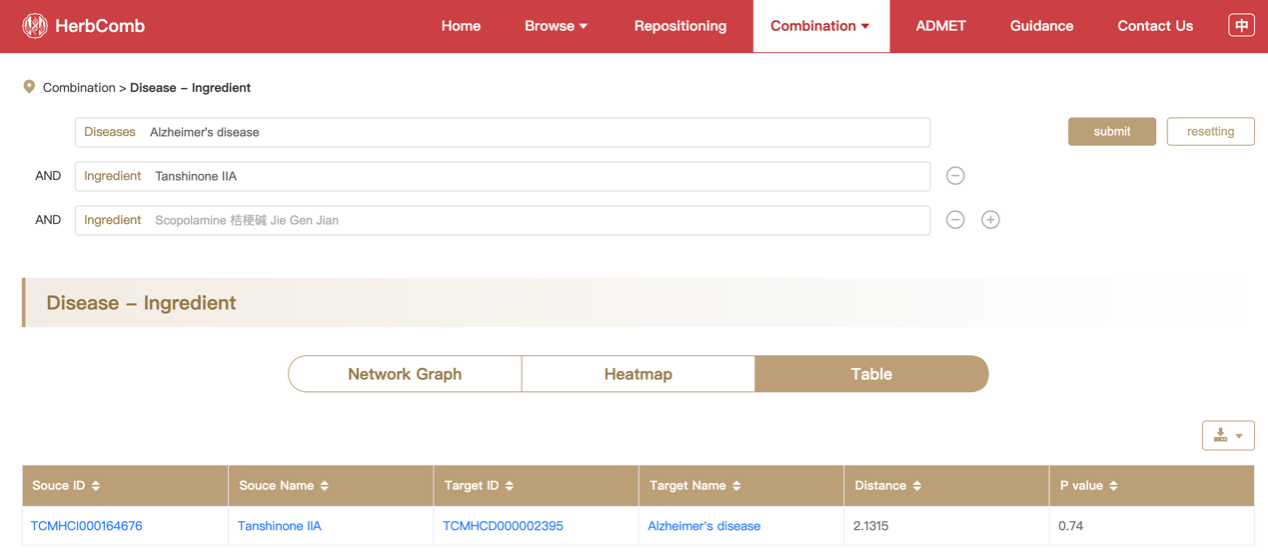


**A Disease-Ingredients Combination List Information, Downloadable.**

## Tutorial for browsing ADMET in HerbComb


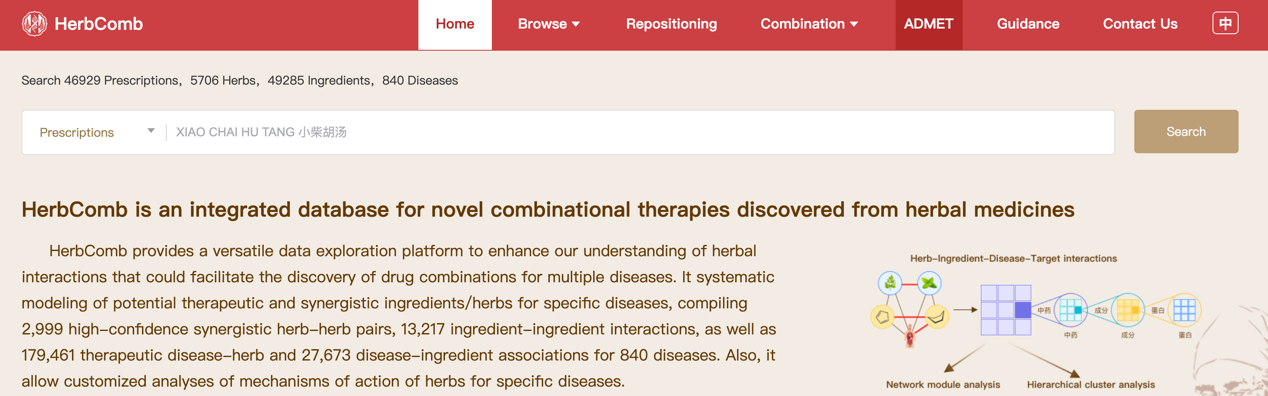


**ADMET Information for All Ingredients of HerbComb**


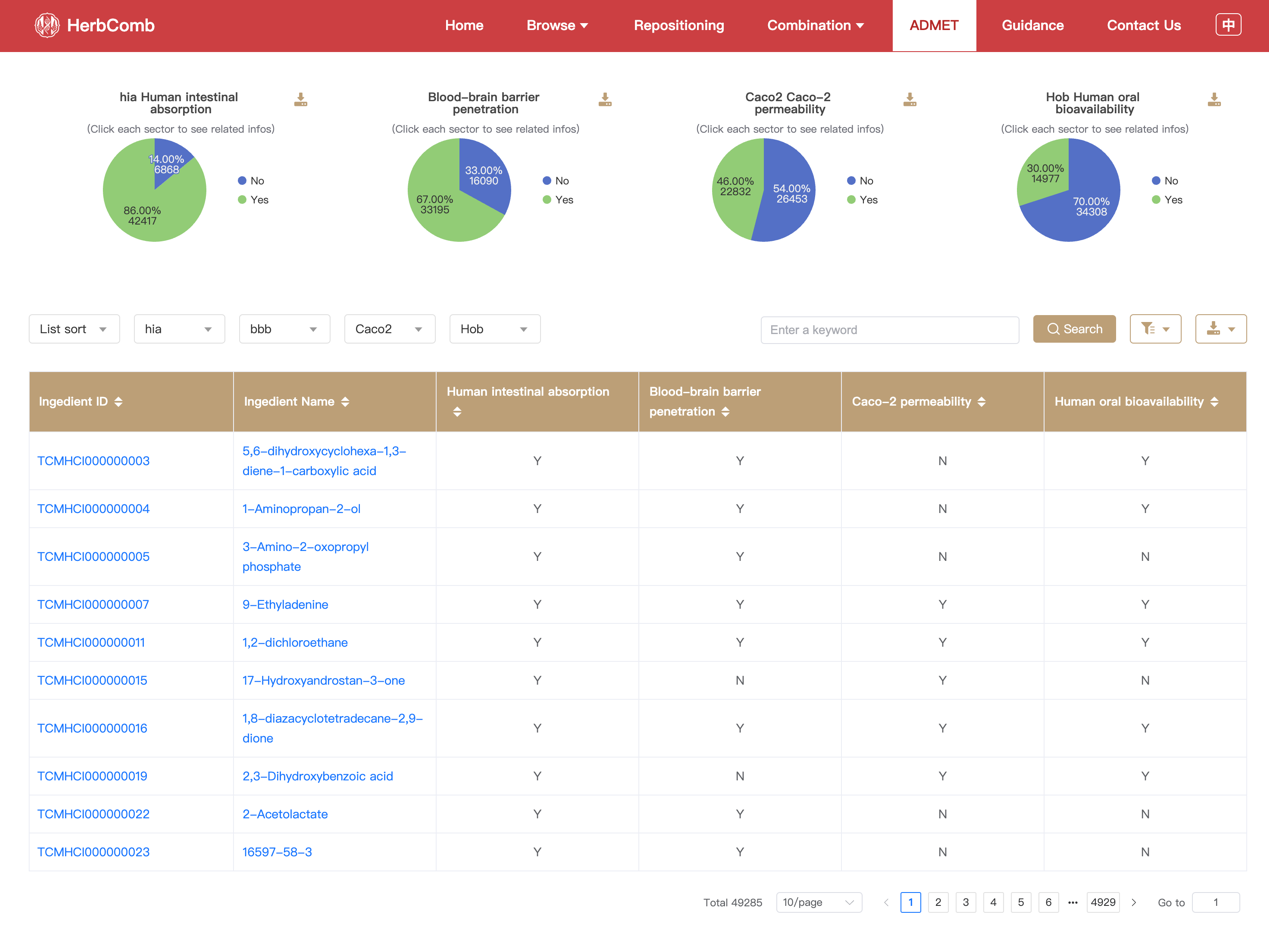


**Click to view details of an ingredient related to a traditional herb.**

**Click to peruse the details of a ingredient.**

**Users can quickly filter ingredients belonging to that category by clicking on the pie chart type.**

## Supplementary Note

# **1. Methodology**

Network proximity methods were employed to explore the combinational relationships among prescriptions further, calculating ingredient-ingredient, herb-herb, ingredient-disease, and herb-disease interactions.

**Ingredients-ingredient synergistic score:** For all potential ingredient pairs within a single formula, we applied a closest distance algorithm based on their target network to calculate their combinational distance. The closest distance algorithm is defined as follows:

$C\mathrm{losest}:\langle d_{AB}^{C}\rangle=\frac{1}{|A|+|B|}\left( \sum_{a\in A} d\left( a,b \right)+\sum_{b\in B} d\left( a,b \right) \right)$ (1)

In function 1, $A$ and $B$ are set of targets separately. $d_{(a,b)}$ is the shortest path length between target $a$ in network $A$ and target $b$ in network $B$. $|A|$and $|B|$ denote the number of nodes in networks $A$ and $B$ separately. For each node $a$ in network $A$, its shortest path length to each node in herb $B$ is calculated and the minimum one will be kept. The exact process is applied to network $B$. Finally, the minimum values of each node in both networks $A$ and $B$ are summed and averaged, indicating how closely these two networks interact. Her, $A$ represents the targets of one ingredient, and $B$ represents the targets of another ingredient. Here, smaller $d\left( X,Y \right)$ indicate strong interactions among PPI networks.

**Herb-herb interaction score:** The distance between herb-herb combinations was calculated based on their ingredient-ingredient network, with the shortest distance as the edge weight. The distance between two herbs is the average shortest distance of their center ingredients:

$Center: \left\langle d_{AB}^{cc} \right\rangle=d\left( {centre}_{A}+{centre}_{B} \right)$ (2)

${centre}_{B}={argmin}_{u\in B}\sum_{b\in B} d\left( b,u \right)$ (3)

Where $B$ is the subnetwork covering all the ingredients in one herb. $d\left( b,u \right)$ represents the shortest path of each pairwise ingredient within a herb. The central ingredient is the one with the minimum sum distance to other ingredients. The shortest algorithm for calculating the distance between two ingredients is defined as follows:

$Shortset:\langle d_{ab}^{S}\rangle=\frac{1}{|a|+|b|}\sum_{a^{,}\in a,b^{,}\in b} d\left( a^{,},b^{,} \right)$ (4)

Where $a^{,}$ and $b^{,}$ are the targets from ingredient $a$ and $b$ separately. The product of $|a|$ and $|b|$ represents the number of targets for ingredients $a$ and $b$. Finally, the shortest $\langle a,b\rangle$ is the sum of all shortest path lengths between the two target sets, averaged to provide a measure of network proximity.

**Ingredient-disease association score:** To measure the association between ingredients and disease, we applied the z-score method. This network module method evaluates the relatedness between one drug and one disease. The shortest path length between drug X and disease Y is defined as:

$d\left( X,Y \right)=\frac{1}{|Y|}\sum_{y\in Y} {min}_{x\in X}d\left( x,y \right) (5)$

$d\left( x,y \right)$ is the shortest path length between target node $x$ in drug $X$ and target node $y$ in disease $Y$. The minimum values across all nodes in disease $Y$ are summed and averaged, similar to the closest distance algorithm but in one direction from $Y$ to $X$.

**Herb-disease association score:** Similar to ingredient-disease associations, we combined all the targets of ingredients in one herb as the target set for one herb. Denoting that $C_{hA}=(C_{1},C_{2}, \ldots, Ci)$ is a set ingredient in herb $A$. $C_{hB}=(C_{1},C_{2}, \ldots, Ci)$ is a set ingredient in an herb $B$.$T_{C}=(T_{1},T_{2}, \ldots,Ti)$ is a set of targets in one compound. Then, the target for one herb can be denoted as:

$T_{H}=T_{C1}\cup T_{C\ldots}\cup T_{Ci}$ (6)

$T_{H}$is the union of targets of ingredients in this herb $X$. We extracted disease-related genes $Y$. The herb-disease proximity was also calculated.

# **2. The distribution of random pairs**

Our null distribution (comprising 304,992 randomly sampled ingredient pairs) approximates a normal distribution (**Figure 3B**). The 5% significance threshold was determined empirically from this distribution. Here, we intentionally included similar ingredient pairs in our random sampling because they reflect real-world TCM practice according to the “JUN-Chen-Zuo-Shi” theory, where similar herbs are combined to enhance therapeutic effects. Excluding them would artificially bias the null distribution against clinically relevant combinations.  However, with over 50,000 ingredients, exhaustive pairwise analysis (approximately 1.25 × 10^9 unique pairs) is computationally prohibitive. Instead, we constructed our null distribution here using all ingredient pairs that co-occur in existing formulae (n = 304,992).  This strategy enables us to capture the proper distribution of target set proximities in our database, which reflects real TCM practice while maintaining statistical validity against an appropriate null model and reducing computational requirements from 2.5 × 10¹² to 10⁵ distance calculations. The extreme 5% tail (lowest network distances) identifies pairs that are significantly closer than 95% of random expectations.

However, the network proximity approach identifies pairs with exceptionally close target interactions, a strong indicator of potential functional interplay (either synergistic or additive). However, it does not pharmacologically quantify the combined effect relative to individual effects, thereby not definitively distinguishing synergy from additivity. Furthermore, this method primarily focuses on identifying potential positive interactions (synergy/additivity) and does not specifically predict antagonistic interactions, which might involve opposing pathways not captured by simple distance minimization. Future work will incorporate antagonism detection through pathway opposition metrics.

# **3. Sensitivity analysis**

We conducted a sensitivity analysis to investigate the distribution of ingredients, diseases, and herbs, identifying hub herbs with a significantly larger number of pairs than others. As shown in **Figures S1A and S1B below, the distribution of herbs, ingredients, diseases, and their associated** pairs is normalized. On average, an herbal formula contains six herbs and 199 ingredients that can be significantly associated with certain diseases. These results suggest that herbal medicines may be valuable resources for drug discovery. Notably, we found that the top 10 hub herbs and ingredients are associated with more than ~300 and ~200 diseases. Similarly, the top 10 hub diseases are associated with ~1,000 herbs and ~200 ingredients separately (**Figure S1A**). On average, each disease is associated with 208 and 32.63 herbs and ingredients, while each herb and each ingredient are associated with 47.30 and 35.39 diseases, respectively (**Figure S1B**). Diseases (n = 840) were classified into 19 therapeutic categories. **Figure S1C-D** shows the distribution of significant herb-disease pairs and significant ingredient-disease pairs across these categories. All pairs of disease-herbs and disease-ingredients are unique, with deduplicated pairs removed. The number of herb-disease pairs per category ranges from 197 to 26,948, with diseases in the Endocrine, Nutritional, and Metabolic classification having the highest representation, at 26,948 related disease-herb pairs. These findings suggest that HerbComb is a valuable data portal for revealing the therapeutic and synergistic ingredients in the TCM formula.


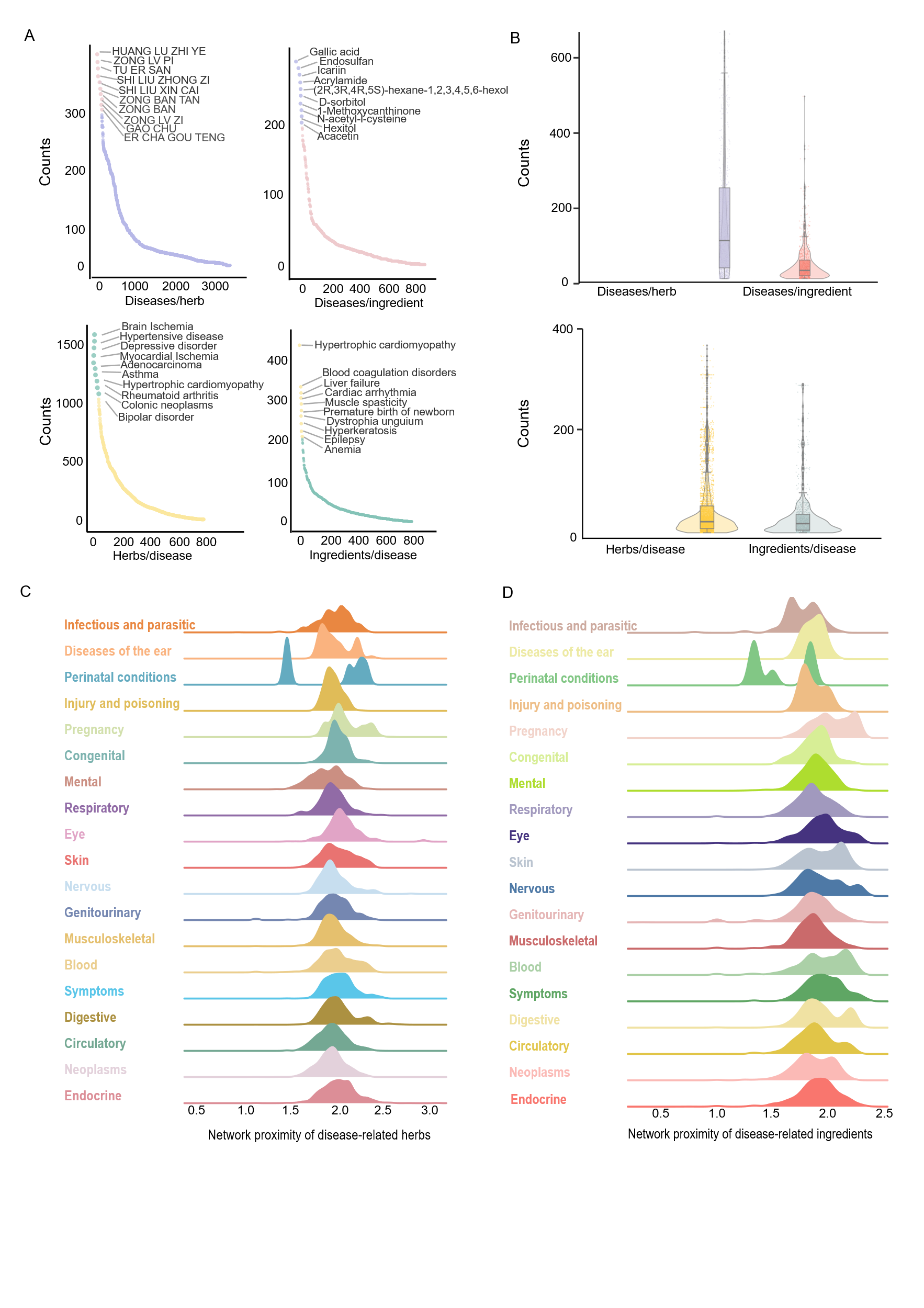


**Figure S1. Distance distribution analysis.** **(A-B)** Distribution of the number of associated diseases per herb, diseases per ingredient, herbs per disease, and ingredients per disease. **(C-D)** The density of network proximity values for significant herb-disease associations **(C)** and ingredient-disease associations **(D)** across 19 therapeutic classes of diseases.

# **4.  Stratified analysis**

As a foundational platform for the broad-scale exploration of herbal synergies, we performed a stratified analysis to investigate whether our findings are consistent across different categories of herbs, diseases, ingredients, and formulas. Firstly, we classified herbs into high- and low-frequency herbs based on their median value in the formulae. We found that higher-frequency herbs show a similar distance to those of lower-frequency herbs (**Figure S2A)**. Then, we divided the formula into two groups based on the herbs that comprise it: simple prescriptions with $N\left( \mathrm{herb}s \right)<6$ and complex prescriptions with $N\left( \mathrm{herb}s \right)\geq6$. Notably, complex prescriptions show significantly closer distances than those simple ones (**Figure S2B)**. To characterize the relationship between herb/ingredient and different disease types, we analyzed the distribution of network distances across 19 disease classifications. The bar plot alongside illustrates the number of diseases contained within each category. We observed a similar distribution of distance, regardless of the number of diseases in the classification (**Figure S2C-D**).

| 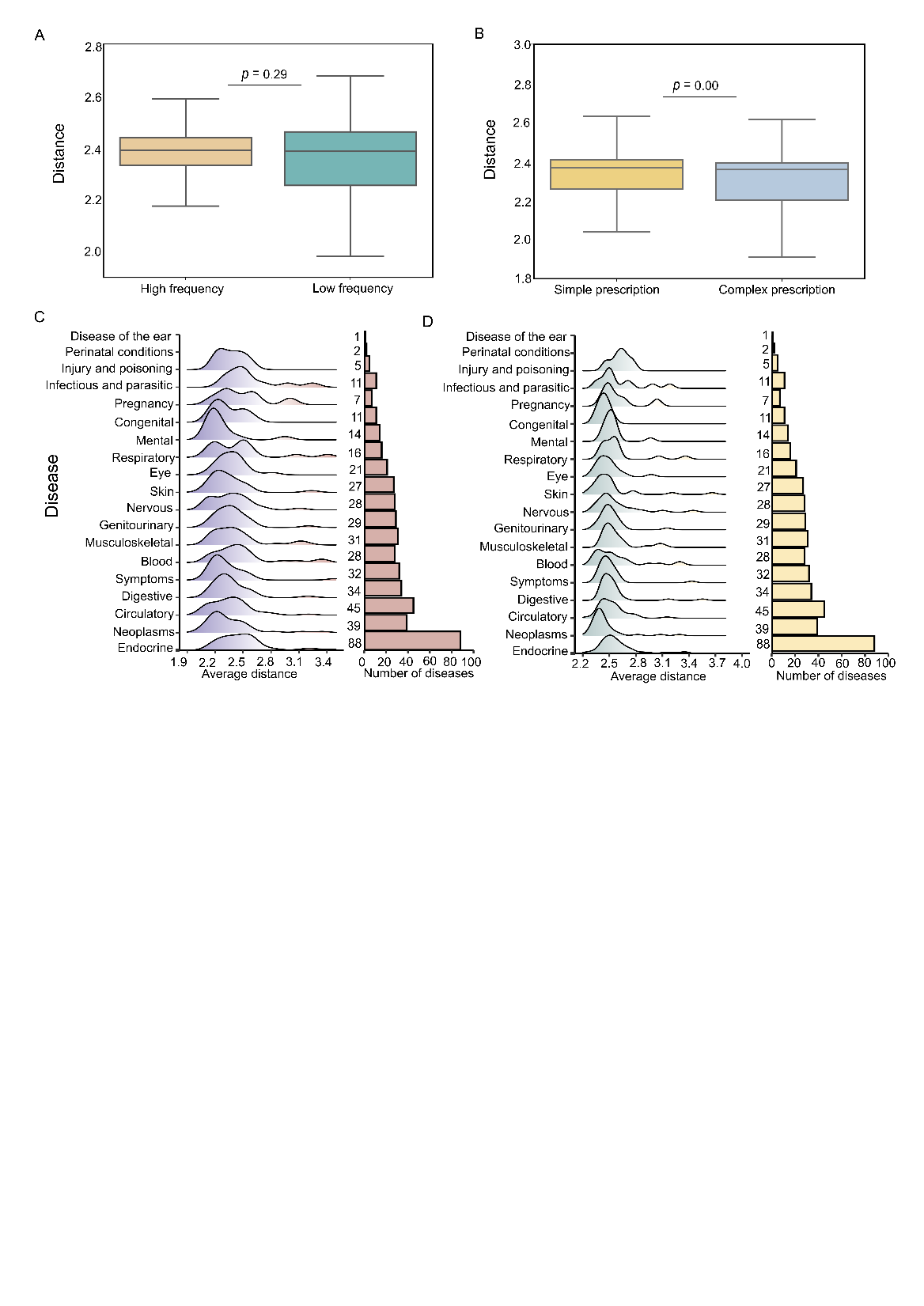 |
| --- |
| **Figure S2. Stratified analysis of distance. (A)** Distance comparison by classifying herbs into high and low frequency herbs by their median value in formulae. **(B)** Distance comparison by cutting the formula into two groups according to the herbs that comprise that formula: Simple prescription with $N\left( \mathrm{herb}s \right)<6$ and complex prescription with $N\left( \mathrm{herb}s \right)\geq6$. **(C-D)** Network distance distributions between herbs/ingredients and diseases across 19 disease classifications. Ridge plots display distance distributions, while bar plots indicate the number of diseases in each category. |

# **5. Case study**

Using Stroke as a case study, we have identified a famous herbal formula, Tongxinluo, in which we have identified Oleanolic acid and Ferulic acid as synergistic ingredients that have not been previously reported.

The TCM formula Tongxinluo Capsule (TXL) has been used clinically for the prevention and treatment of cardiovascular diseases, particularly ischemic stroke. However, existing research primarily focuses on its overall efficacy, with limited knowledge of the interactions between its ingredients. Using HerbComb, we determined the interaction distances between herbs and ingredients associated with ischemic stroke.

The combinational effects of TXL were further investigated by constructing a network to represent the interactions between ingredients and diseases (**Figure S3A**). To identify key active components and potential synergistic ingredients, the Louvain algorithm was employed for community detection, based on the assumption that ingredients clustered with the ischemic stroke node are more likely to show synergistic effects. According to ingredient-disease and ingredient-ingredient distance analysis, Oleanolic acid and Ferulic acid were ultimately selected (**Figure S3B**).

To validate the synergistic effects of Oleanolic acid and Ferulic acid, we conducted a cell viability assay on neural protection using the HT22 cell line (immortalized mouse hippocampal neuronal cells). We found that the combination of Oleanolic acid and Ferulic acid at both low and high concentrations shows significantly increased cell viability in the HT22 cell line (T-test, *P* < 0.01, **Figure S3C**). These results suggest that the HerbComb database can be an effective platform for discovering synergistic ingredients and gaining deeper insights into the mechanisms of action of herbal medicines.

| 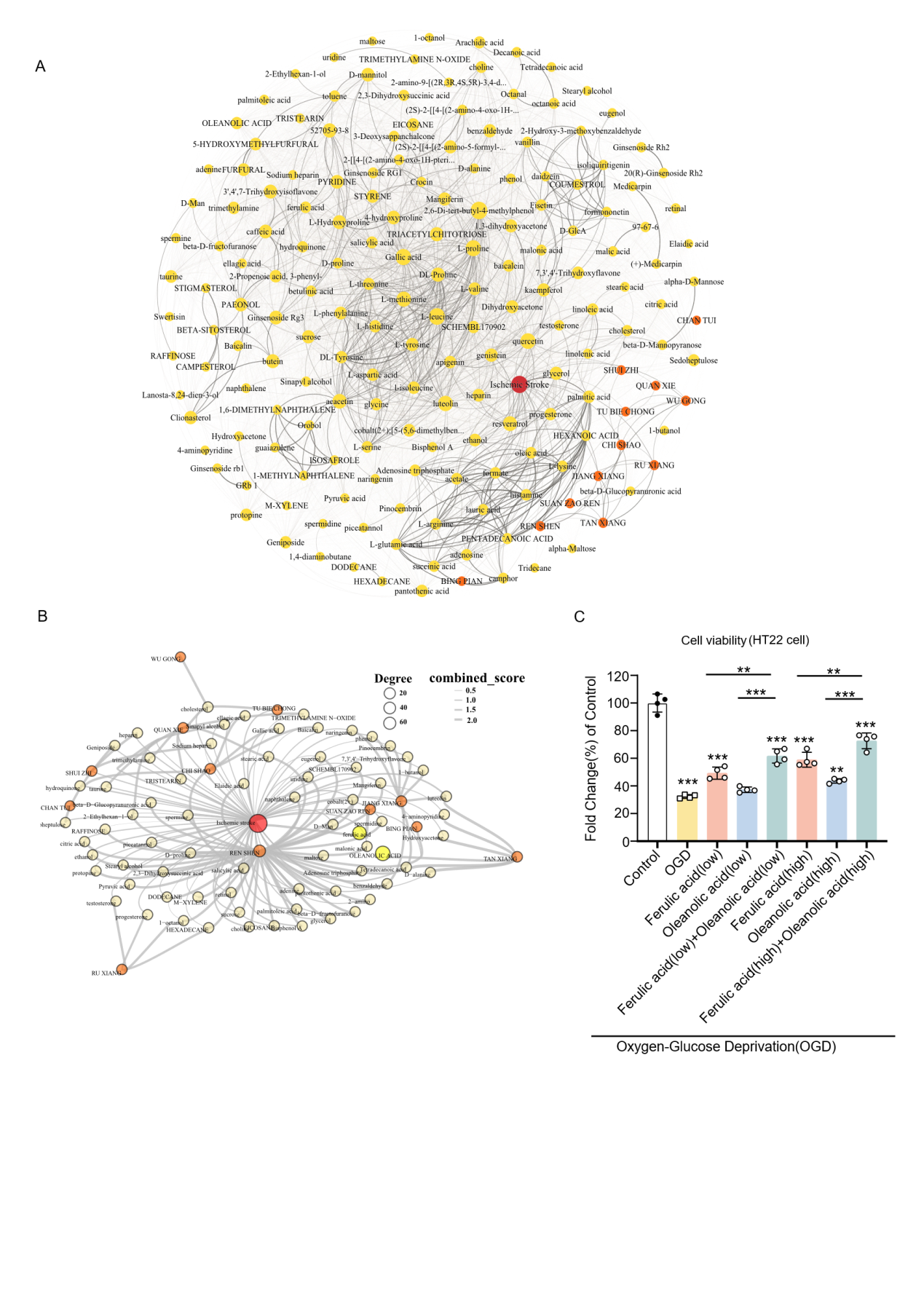 |
| --- |
| **Figure S3. The combinatorial landscape of Tongxinluo Capsule.** (A) Herb-Component-Disease Network. (B) Key disease-associated module via network community analysis. (C) The viability of HT22 cells in Oxygen and Glucose Deprivation (OGD) conditions of different concentrations of Oleanolic acid and Ferulic acid (T-test, * *P* < 0.05, *** *P* < 0.01, *** *P* < 0.001). |

# **6. Limitations and future steps**

1. The network proximity approach identifies pairs with exceptionally close target interactions, a strong indicator of potential functional interplay (either synergistic or additive). However, it does not pharmacologically quantify the combined effect relative to its components, thereby failing to distinguish synergy from additivity definitively. Furthermore, this method primarily focuses on identifying potential positive interactions (synergy or additivity). It does not specifically predict antagonistic interactions, which may involve opposing pathways not captured by simple distance minimization. Future work will incorporate antagonism detection through pathway opposition metrics.
2. While we have standardized herbs, ingredients, diseases/conditions, and target ontologies, aligning biomedical classifications remains inherently challenging. For example, certain conditions lack ICD-10-CM categorization and are labeled as 'NA.' These limitations stem from the current limitations of ontology tools and gaps in the source databases. We will continuously update our platform as ontologies evolve (e.g., ICD-10-CM updates) and receive user feedback.
3. HerbComb provides ADMET-aware screening (identifying 9 of 32 ischemic stroke-targeted compounds with favorable bioavailability and blood-brain barrier permeability in **Figure 3C**). However, these predictions serve only as initial pharmacokinetic filters. Although ADMET profiling offers valuable insights, true organism-level effects require consideration of additional biological complexities, including gut microbiome interactions (biotransformation, metabolic activation), tissue-specific distribution, and off-target signaling effects. More importantly, HerbComb is a hypothesis-generating tool. Thus, all predictions require experimental confirmation via in vitro, organoid, or in vivo studies. Additionally, traditional knowledge remains essential for contextualizing results. Future improvements will incorporate microbiome interaction predictions, dynamic PK/PD modeling, and clinical correlation data to enhance combination drug discovery.
4. More importantly, HerbComb is a hypothesis-generating tool. Thus, all predictions require experimental confirmation via in vitro, organoid, or in vivo studies. Additionally, traditional knowledge remains essential for contextualizing results.
5. While we have established a comprehensive platform for the combinatorial analysis of herbal medicines, several methodological constraints arising from data limitations warrant consideration. First, our approach inherently prioritizes well-studied herbs (e.g., *Glycyrrhiza uralensis*, *Panax ginseng*) with abundant target data, potentially overlooking under-investigated botanicals. This reflects systemic biases in existing literature and databases used for compilation. Consequently, network predictions may favor frequently studied herbs, potentially overlooking synergistic potential in understudied botanical resources. Similarly, our manually curated interaction dataset may overrepresent positive findings due to the preferential publication of synergistic results versus neutral/antagonistic outcomes. Third, high-confidence synergistic pairs often correspond to classical formulations (e.g., *Coptis chinensis* and *Scutellaria baicalensis* in Huang-Lian-Jie-Du-Tang). While validating traditional knowledge, this may limit the discovery of novel combinations that extend beyond established paradigms. Our network proximity approach partially mitigates this by evaluating biological relationships rather than co-prescription frequency.
